# Intervention on default contagion under partial information in a financial network

**DOI:** 10.1371/journal.pone.0209819

**Published:** 2019-01-15

**Authors:** Yang Xu

**Affiliations:** Industrial Engineering and Operations Research, University of California at Berkeley, Berkeley, CA, United States of America; Central South University, CHINA

## Abstract

We study the optimal interventions of a regulator (a central bank or government) on the illiquidity default contagion process in a large, heterogeneous, unsecured interbank lending market. The regulator has only partial information on the interbank connections and aims to minimize the fraction of final defaults with minimal interventions. We derive the analytical results of the asymptotic optimal intervention policy and the asymptotic magnitude of default contagion in terms of the network characteristics. We extend the results of Amini, Cont and Minca’s work to incorporate interventions and adopt the dynamics of Amini, Minca and Sulem’s model to build heterogeneous networks with degree sequences and initial equity levels drawn from arbitrary distributions. Our results generate insights that the optimal intervention policy is “monotonic” in terms of the intervention cost, the closeness to invulnerability and connectivity. The regulator should prioritize interventions on banks that are systematically important or close to invulnerability. Moreover, the regulator should keep intervening on a bank once having intervened on it. Our simulation results show a good agreement with the theoretical results.

## Introduction

### Introduction

The systemic risk in a financial network has been drawing more and more interest of regulators and researchers, especially after the Asian financial crisis in the late 1990’s and the more recent economic recession during 2007-2009. Financial institutions (hereafter, banks) are connected to form an interbank network to allow liquidity reallocation between the banks in that banks with liquidity surpluses can lend to banks with liquidity deficits. However, the interbank network may also introduce aggregate liquidity shortage and default contagion. Liquidity shock such as a run will cause some banks to default, leading to losses of their creditor banks through interbank connections, which may in turn result in losses of their creditors. In the financial crisis of 2007-2009 the interbank market dysfunctioned because the market participants perceived heightened counterparty risk and liquidity risk [1], and the severe reduction in transaction volume was a major contributing factor to the collapse of many banks. When the interbank market is stressed or freezes, the central bank as the lender of last resort has to provide extensive short term liquidity support. For example, the Federal Reserve in the US established many facilities, including the traditional discount window, the Term Auction Facility (TAF), Primary Dealer Credit Facility (PDCF), and Term Securities Lending Facility (TSLF). The central bank or government also recapitalize the banks and provide risk capital in the form of a bailout. Naturally, we ask the following questions: Should the regulator (the central bank or government) intervene if the bankruptcy of one or several banks has occurred or is imminent? What is the optimal intervention policy of the regulator based on the measurable features of the network and the banks, such as the degrees of connectivity and the levels of capitalization? How much improvement can the optimal strategy achieve regarding the fraction of market protected from defaults?

To answer these questions we study the uncollateralized interbank funding market where the majority of interbank loans are overnight. The connections are constantly changing so the regulator may not know exactly all the connections over time. After some liquidity incident, e.g. runs on a few banks, some banks default, which initiates the default contagion process. During the process, the regulator has to intervene on the defaulted banks to prevent the contagion from spreading. Due to the system panics, no banks want to lend new loans to other banks but meanwhile they are still obliged to pay back their current loans which are due in the time frame of the model. So it is reasonable to fix the in and out degrees of banks and assume all the connections do not change any more after the inception of default contagion. Theoretically, the regulator would be able to find out the connections between the banks by communicating with the banks; however, the contagion process happens so fast that the regulator may not have ample time to find out the connections between the banks. In other words, the regulator will have to intervene while all the connections between the banks are unknown. So the regulator has only partial information because in the beginning the regulator only knows the initially defaulted banks and the magnitude of connections of each bank but the connections between the banks are unknown. Every time a default occurs the regulator learns the connections of the defaulted bank (i.e. the banks that are affected by the default), represented by the connections being revealed after the default.

We set up a probability space under which the financial network is generated by a uniform matching of the in and out degrees (a configuration model). A directed link in the network represents one unit of loan. In the following we may use “bank” and “node” interchangeably. Before an external shock to the system, each node has a positive equity level (the difference of total assets and liabilities), which indicates the number of defaulted loans due to the default of its debtors a node can withstand before it defaults. In other words, it is the “distance to default”. After an external shock, some nodes in the system default initially and we set their equity levels to zero. We adopt the dynamics of the model in [2, 3]. When a node defaults, it defaults on all of its loans. We assume a zero recovery rate of the loan, i.e. the creditor receives zero value from the loan, which is the most realistic assumption for short term default as suggested in [4]. We assume there is a time span between a node’s default and the time its creditor records the loan as a loss (by writing down the loan from its balance sheet). We model this time span by independently and identically distributed exponential random variables. After the affected node records the defaulted loan, it may request the regulator for interventions. If the regulator decides to intervene by replacing the defaulted loan or by infusing one unit of equity, the equity level of the affected node will stay the same, otherwise its equity level will decrease by one. Once the equity level reaches zero, the node defaults. We assume that the once a bank has defaulted it cannot become liquid again within the time horizon of the model because it is very unlikely for a bank that has declared default to gain enough capital in the short term considered in the model.

We emphasize that one essential feature of the model is partial information. Because it takes time and effort for the regulator to find out the exact connections in the interbank market and the default contagion process may progress very rapidly, the regulator may have to intervene even before it is able to figure out the connections in order to take early actions and save the maximum amount of banks from bankruptcy. During the contagion process, the regulator only knows the default set (the set of defaulted nodes) with some out-links revealed, but meanwhile other out-links remain hidden until the affected nodes record the defaulted loans. More generally, unlike the complete information assumption in other theoretical models, the partial information setting aligns with the reality better, as pointed out by [5]: “*Interbank exposure data are never publicly available, and in many countries nonexistent even for central regulators*”.

**Fig 1:**
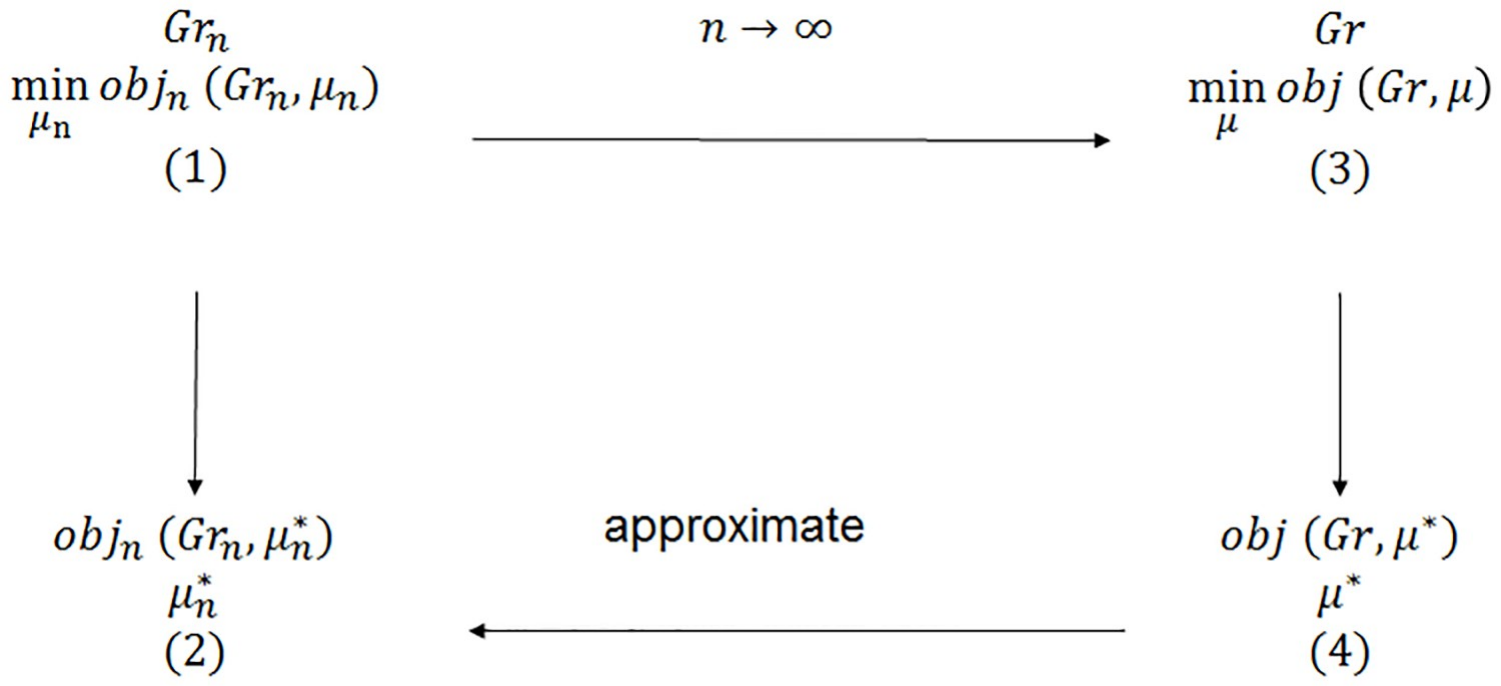
**The methodology**: Approximation of the finite network with the infinite network.

Our methodology is illustrated in Fig 1. The regulator’s goal is to minimize the number of final defaulted nodes with the minimum number of interventions. Thus we obtain a stochastic control problem $\min_{\mu_{n}}{\text{obj}}_{n}\left(Gr{}_{n},\mu_{n}\right)$ where the objective function depends on the graph *Gr*_*n*_ and the intervention sequence *μ*_*n*_, shown in (1). We aim to solve it for the optimal intervention sequence $\mu_{n}^{*}$ and thus obtain the optimal objective function value ${\text{obj}}_{n}\left(Gr_{n},\mu_{n}^{*}\right)$, shown in (2). However, solving the problem with the usual dynamic programming approach will incur intractability problem because of the fast expansion of the state space as pointed out in [3], especially for a heterogeneous network. We take an alternative approach based on the fact that under some regularity conditions, the objective function converges as *n* → ∞. We solve the asymptotic optimal control problem min_*μ*_ obj(*Gr*, *μ*) in (3) where obj, *Gr* and *μ* are the limit forms of obj_*n*_, *Gr*_*n*_ and *μ*_*n*_, respectively, and obtain the optimal intervention μ* and the objective function obj(*Gr*, μ*) in (4) which allow us to construct the optimal intervention sequence $\mu_{n}^{*}$ for a finite *n* through μ* and approximate ${\text{obj}}_{n}\left(Gr_{n},\mu_{n}^{*}\right)$ with obj(*Gr*, μ*). Our results of the numerical experiments validate the approximation for networks with sizes close to the real financial networks.

### Relations to previous literature

Our work is closely related to the current literature on the role of the central bank as the lender of last resort, including providing liquidity by a loan, recapitalizing the banks and bank bailout. These studies differ in their perspectives and focuses in their models. The influencing Diamond & Dybvig model [6] about the market panics and bank runs has two Nash equilibria: depositors withdraw only for their real expenditure needs or a bank run. [7] extend the Diamond & Dybvig model to a financial network of four banks to study the default contagion. The interbank network is formed to allocate liquidity among the banks to satisfy regional liquidity demands. In a complete market where the banks exchange deposits or in a disconnected market no contagion occurs while in an incomplete market where the banks do not exchange deposits with all other banks, high connectivity may entail contagion. The role of the central bank is thus to complete the market. [8] introduce a similar model but assume the depositors are uncertain about where they have to consume. In their model the central bank acts as a “crisis manager”: when a bank is to be liquidated, the central bank has to organize the bypass of the defaulting bank in the payment network and provide liquidity to the banks that depend on the defaulting bank. [9] consider that the fire sales of the banking assets occur when a large number of banks default and investors outside the banking sector who are inefficient users may end up purchasing the liquidated assets. To avoid the allocation inefficiency, the regulator may bail out the banks directly or provides liquidity to surviving banks to purchase defaulting banks. [10] argue that the government should bail out banks in distress because it can provide liquidity more efficiently than private investors. [11] consider three forms of regulator interventions: buying equity, purchasing assets and providing debt guarantee to alleviate debt overhang in a financial market but the regulator has limited information and resources. All the works discuss the optimal interventions based on equilibrium analysis. In contrary, our model emphasizes the aspect of the interbank market that it is a complex network and focus on how the regulator should make intervention decisions under the network dynamics. Moreover, some papers study the related problem about the banks in the interbank network bailing each other out, such as [12] and [13].

In addition to the theoretical studies, empirical studies abound. [14] analyze the data on the interbank transactions derived from the main euro area payment system and find that the European Central Bank took the role of the overnight unsecured interbank market in liquidity provision to the banks during the global financial crisis in 2008-2010. [15] analyze the daily transaction data and find that in 2008 counterparty risk plays a more important role than liquidity hoarding in reducing liquidity and increasing the cost of finance in the federal funds market in the US. By analyzing supervisory data of Germany, [16] find that regulatory interventions decrease liquidity creation while capital support does not affect it. [17] discuss the relations between liquidity regulations and the lender of last resort practice and argue that they are complementary rather than conflicting tools.

Our work is also related to the strand of literature on systemic risk and default contagion in the financial networks without considering regulator interventions. These works focus on understanding the dependence of the default contagion on various features of the financial network and the banks within it, including the degrees of connectivity, the equity levels and so on. Similarly, there are mainly two types of literature: empirical and theoretical. The empirical studies conduct statistical analyses on the interbank markets using data on interbank lending as far as they are available and provide an overview of the structural characteristics of the interbank network in different countries ([4, 18, 19]). The theoretical studies model the financial network with network models but differ in their assumptions about the network structure and approaches: some focus on “stylized” networks whose structures are hypothetical ([7, 20, 21, 22]) while others rely on simulations ([4, 23]). Among them, [24] and [25] propose random network models that allow more realistic and heterogeneous structures. [26] survey theoretical works on contagion and systemic risk in financial networks and categorize them according to different topics including network connectivity, bank heterogeneity, uncertainty in financial markets, and portfolio composition of the banks.

In regards to section Introduction, this paper is closely related to [24] and [2, 3]. [24] study the magnitude of default contagion in a heterogeneous network with given degree sequence and arbitrary distribution of weights and derive the analytical expressions of the asymptotic fractions of defaults in terms of the network characteristics. Our work incorporates interventions into a model proven to be equivalent to theirs. Thus if there are no interventions, the asymptotic fraction of final defaults will be the same as in [24]. [2] consider a stylized core-periphery financial network as an intermediary to provide liquidity to fund projects in outside economy but it may also incur contagion when the banks hoard liquidity. The regulator intervenes by providing loans to defaulting banks. [3] consider a similar core-periphery model where the regulator intervenes by injecting equity. We adopt the dynamics of their model that constructs the default set under interventions through a configuration model because the configuration model can be adapted to the contagion process [27]. But we differ from [2, 3] in two important ways. First, our model is a more general heterogeneous random network with degree sequences and initial equity levels drawn from arbitrary distributions. More importantly, [2, 3] focus on the benefits and costs of the connectivity in the presence of the regulator and draw conclusions mainly from numerical studies, while we focus on the optimal intervention policy and its relations to the network characteristics. Mathematically we have successfully addressed two major mathematical difficulties arising from considering interventions on a general complex network: Interventions introduce discontinuity into the asymptotic process thus the main supporting theorem used in [24] is no longer applicable directly; moreover, the high dimensional optimal control problem we obtain later is well known among control theorists difficult to solve, especially analytically. We give analytical formulations for the asymptotic optimal intervention policy as well as the asymptotic number of interventions and final defaulted banks. The asymptotic results provide a good approximation to real financial networks, which are heterogeneous and have several hundred to a thousand of banks thanks to the fast convergence behavior of our results.

### Contributions

The main contribution is that we have proposed a new approach to determine the optimal intervention strategy on contagions in a large and heterogeneous financial network-to be specific, we derive the asymptotic optimal intervention strategy as the size of the network tends to infinity and then show that it is a good approximation of the optimal intervention strategy for a real financial network. This new approach has the advantage of avoiding the stylized model in the equilibrium analysis or the intractability of the dynamic programming approach in previous literature and enabling us to obtain analytical results. In light of this, we derive rigorous asymptotic results of the optimal strategy for the regulator and the fraction of final defaulted banks under the dynamics of the default contagion process in a heterogeneous network with a degree sequence and initial equity levels that can be drawn from arbitrary distributions. The analytical expressions are presented in terms of measurable features of the network. For convergence of the results, we assume the network is sparse as in [24] which is supported by the empirical studies of real financial networks ([28, 29]).

The key insights of our findings of the optimal strategies are summarized in the following. We should only consider intervening on a bank when it records the loss of a defaulted loan and is very close to default. The optimal intervention policy depends strongly on the intervention cost. The smaller the intervention cost, the more interventions are implemented. Moreover, the optimal intervention policy is “monotonic” with respect to the measurable features of the network. We should not intervene on the banks with out degrees in a certain range regardless of their other features; for those banks worth interventions, the larger the sum of initial equity and accumulative interventions received, the earlier we should begin intervention on them; the time to start intervention on a node is also “monotonic” in its in and out degrees. Interestingly, once we begin intervening on a node, we keep intervening on it every time it records the loss of a defaulted loan. By comparing the fractions of final defaults under no interventions and the optimal intervention policy, we are able to quantify the improvement made by interventions in terms of the network features. This gives guidance for the maximum impact the regulator can have to offset the effects of default contagion.

The paper is organized as follows. We set up the model and introduce the stochastic control problem (SCP) in section Model description and dynamics. In section The asymptotic control problem we formulate the asymptotic control problem Eq (9) that gives the limit for the objective function of the SCP as the size of the network goes to infinity and present the necessary conditions for the optimal intervention policy, which lead to the main theorems. In section Numerical experiments we show the results of the numerical experiments to validate the approximation of Eq (4) by Eq (9). We present in Appendix A: Proofs all the proofs and in Appendix B: Wormald’s theorem and Appendix C: Extended pontryagin maximum principle the two theorems used in the proofs as well as a list of notations in Appendix D: Preliminary list of notations.

## Model description and dynamics

### Basic setup

We consider default contagion in an unsecured interbank lending market under short term illiquidity risk based on the model of [2]. Due to the system panics, no banks want to lend new loans to other banks but meanwhile they are still obliged to pay back their current loans which are due in the time frame of the model, so we fix the in and out degrees of banks in the network, denoted as (*d*^−^(*v*), *d*^+^(*v*))_*v*∈[*n*]_, where [*n*] = {1, …, *n*} the set of nodes. Let *m* = ∑_*v*∈[*n*]_ *d*^−^(*v*) = ∑_*v*∈[*n*]_ *d*^+^(*v*).

Then we model the financial network with prescribed degree sequence (*d*^−^(*v*), *d*^+^(*v*))_*v*∈[*n*]_ as an unweighted directed network $\left(\left[n\right],E_{n}\right)$, where $E_{n}$ denotes the set of links. A directed link $\left(v,w\right)\in E_{n}$ represents *v* borrows a unit of loan from *w*, i.e. *v* is obliged to repay *w* one unit of loan. We allow multiple loans to exist between two nodes and also self links representing the internal loans between different departments of the bank.

Now we set up a probability space $\left(G_{n,m},P\right)$ where *G*_*n*,*m*_ is the set of networks on *n* nodes with at most *m* directed links. So the random financial network with *m* directed links lives in this probability space and under P the law of the random link set $E_{n}$ is determined as follows. We start with *n* unconnected nodes and assign node *v* *d*^−^(*v*) in-half-links and *d*^+^(*v*) out-half-links. An in-half-link represents an offer of a loan and an out-half-link a demand for a loan. Then the *m* in-half-links and *m* out-half-links are matched uniformly so that the borrowers and lenders are determined. The resulting random network is called the *configuration model*.

The uniform matching of the in and out-half-links allows us to construct the random network sequentially: at every step we can choose any out half-link by any rule and choose the in-half-link uniformly over all unconnected in-half-links to form a directed link. This is because conditional on any subset of connected links, the unconnected links also follow the uniform distribution. Moreover, the conditional law of unconnected links only depends on the number of connected links, not the matching history. Additionally we can restrict the matching to choosing only the out-half-links from the defaulted nodes so that we can model the development of the default set with their revealed out-links.

**Remark 1**. As a result of the uniform connection of in and out-half-links, a node gets selected with the probability proportional to the number of its unconnected in-half-links. The rationale is that when the regulator searches for the lender of a defaulting loan, a bank that lends out more loans to other banks is more likely to be the lender and be affected by the defaulting loan.

Then we endow a node *v* ∈ [*n*] with its initial equity level $e\left(v\right)\in N_{0}≔\{0,1,2,\ldots \}$ which represents the number of defaulted loans *v* can tolerate until *v* defaults, so it is the “distance to default”. Next after the system receives some external shock, some nodes default and the system begins to evolve. Define time 0 as the time right after the shock. Let ${\left(G_{k}\right)}_{0\leq k\leq m}$ be the filtration for the probability space $\left(G_{n,m},P\right)$ which models the arrival of new information, i.e. the revealed link at each step. Because this implies that the the remaining equity of the selected node will decrease by one, ${\left(G_{k}\right)}_{0\leq k\leq m}$ also models the default contagion at the same time. Note in the following the network with the set of revealed links evolves in the space *G*_*n*,*m*_ as the result of the contagion process.

#### Initial condition

**Fig 2:**
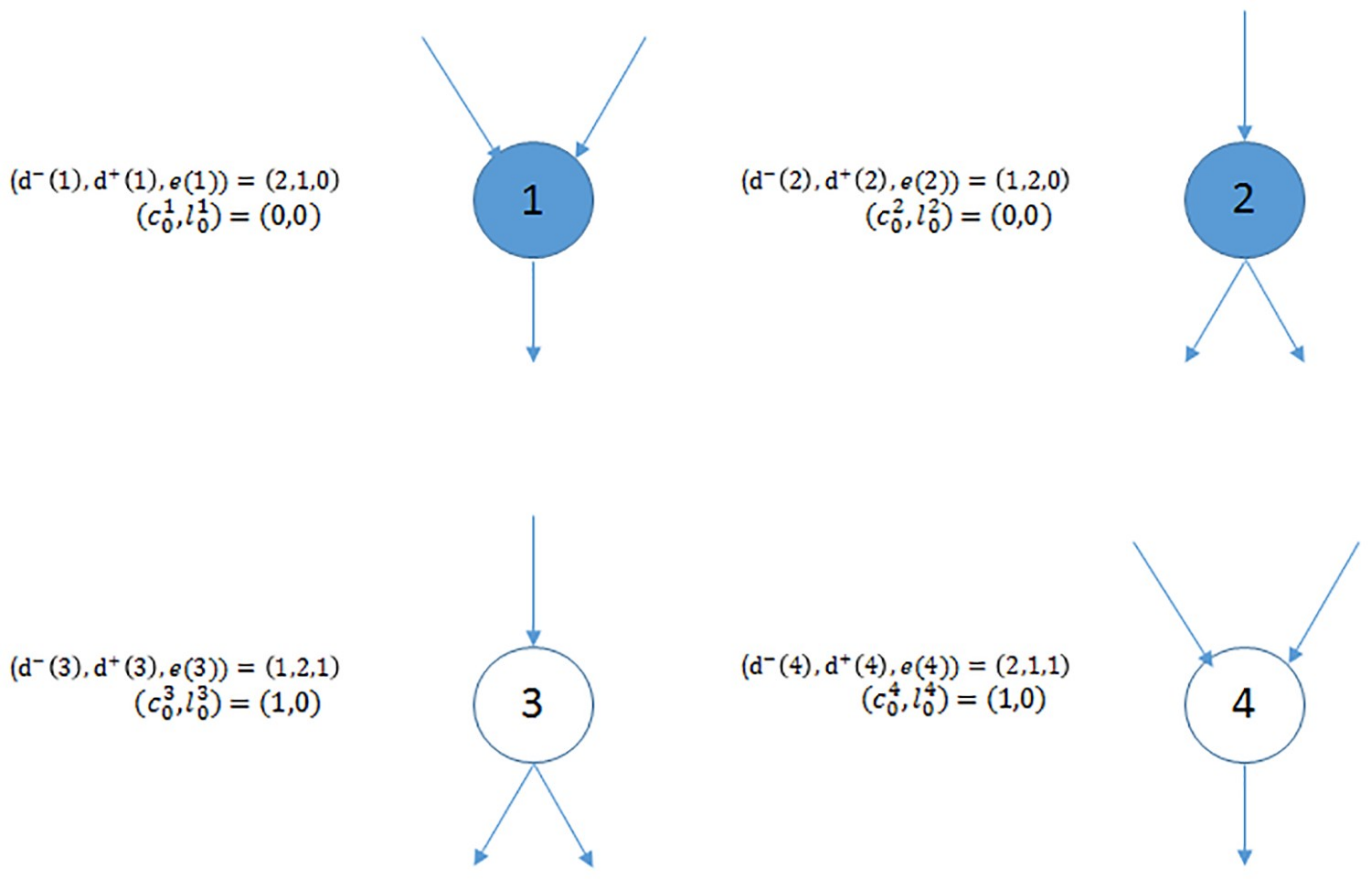
Financial network before default contagion occurs. (*d*^−^(*v*), *d*^+^(*v*)) are the degrees and *e*(*v*) is the initial equity of node *v*. The nodes in the initial default set $D_{0}=\left\{1,2\right\}$ are marked in blue.

Define the set of initially defaulted nodes $D_{0}≔\{v\in [n]:\;e(v)\leq 0\}$ and the *σ*-algebra representing the information available initially $G_{0}≔\sigma ({\left(d^{-}\left(v\right),d^{+}\left(v\right),e\left(v\right)\right)}_{v\in [n]})$. Given the degree pairs for all nodes (*d*^−^(*v*), *d*^+^(*v*))_*v*∈[*n*]_, envision for a node *v* ∈ [*n*], there are *d*^−^(*v*) in-half-links each representing a loan another node is obliged to pay *v* and *d*^+^(*v*) out-half-links each representing a loan *v* is obliged to pay another node. Let $c_{k}^{v}$ be the sum of initial equity and accumulative number of interventions on node *v* and $l_{k}^{v}$ be the number of revealed in-links of node *v* at step *k*, so $c_{0}^{v}=e\left(v\right)$, $l_{0}^{v}=0$. An example of the initial condition of a four node network is illustrated in Fig 2 with $D_{0}=\left\{1,2\right\}$.

#### Dynamics

We adopt the dynamics from [2]. At the *k*th step for *k* ∈ [1, *m*], if the out-links of nodes in $D_{k-1}$ have not all been revealed, then the new link is revealed following the rule: an out-half-link of any node in $D_{k-1}$ is picked by any rule and then connected uniformly to another unconnected in-half-link. Let (*V*_*k*_, *W*_*k*_) be a pair of random variables denoting the link from node *V*_*k*_ to node *W*_*k*_ being *revealed* at step *k*. We call *W*_*k*_ is *selected* at step *k*. Assume (*V*_*k*_, *W*_*k*_) = (*v*, *w*), then the uniformity in connecting the half-links leads to the probability of *w* being selected conditional on $G_{k-1}$ as

$$\begin{array}{c} \;P(W_{k}=w|G_{k-1})\\ =\frac{\text{number}\;\text{of}\;w’\text{s}\;\text{unrevealed}\;\text{in-links}\;\text{at}\;k-1}{\text{total}\;\text{number}\;\text{of}\;\text{unrevealed}\;\text{in-links}\;\text{at}\;k-1}\\ =\frac{d^{-}\left(w\right)-l_{k-1}^{w}}{m-(k-1)}. \end{array}$$ (1)

So a node is selected with the probability proportional to the number of its unrevealed (unconnected) in-half-links. After a directed link (*v*, *w*) is revealed, then proceed with the following steps:

- Update $G_{k}=\sigma (G_{k-1}\cup \{(v,w)\})$.
- Update the number of revealed out-links: $l_{k}^{w}=l_{k-1}^{w}+1$ and $l_{k}^{\eta }=l_{k-1}^{\eta }$ for *η* ≠ *w*.
- Determine the intervention *μ*_*k*_ ∈ {0, 1} $G_{k}$ measurable at step *k* for the selected node *w*. Note that $c_{k}^{\eta }\leq l_{k}^{\eta }$ indicates that the node *η* has defaulted by step *k*. Because we do not intervene on defaulted node, *μ*_*k*_ = 0 if $c_{k-1}^{w}\leq l_{k-1}^{w}$.
- Update $c_{k}^{w}=c_{k-1}^{w}+\mu_{k}^{w}$, otherwise $c_{k}^{\eta }=c_{k-1}^{\eta }$ for *η* ≠ *w*.
- Update the default set $D_{k}$. If $c_{k}^{w}\leq l_{k}^{w}$ and $w\notin D_{k-1}$, then $D_{k}=D_{k-1}\cup \left\{w\right\}$, otherwise $D_{k}=D_{k-1}$.

**Fig 3:**
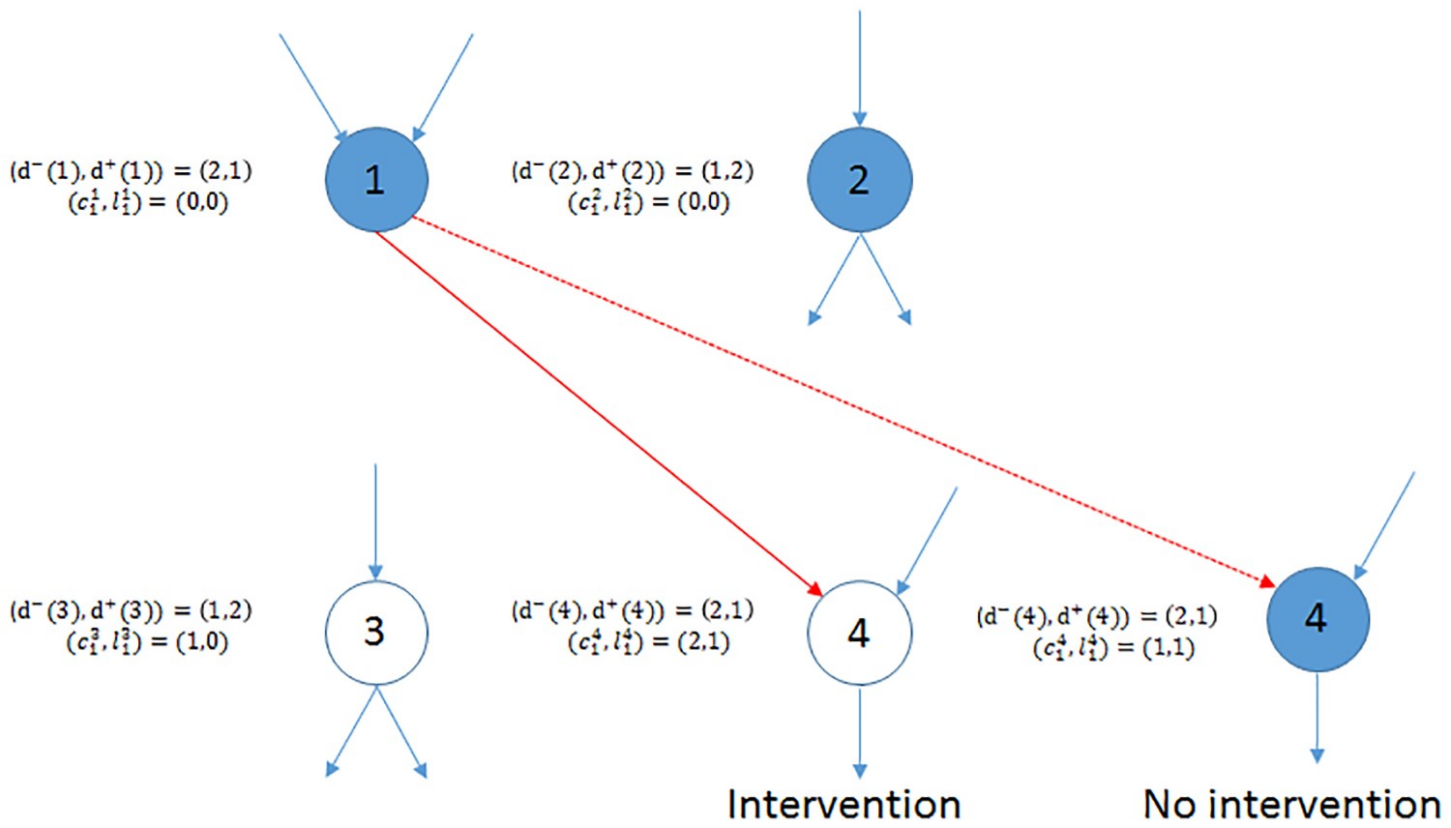
Dynamics at step one. Node 4 is selected with probability $\frac{2}{6}$ and a link between 1 and 4 is revealed. If one intervention is applied, node 4 remains liquid, otherwise its equity level will decrease by one and node 4 defaults.

If all out-links from $D_{k}$ have been revealed, the process ends and let the process end time be *T*_*n*_ = *k*, otherwise repeat the process. Define $D_{T_{n}}$ as the number of defaulted nodes by the process end time *T*_*n*_. In Fig 3 we show the first step of the dynamics for the network in Fig 2. The link (1, 4) is revealed (connected) and node 4 is selected with probability $\frac{2}{6}$. Because the node 4 is liquid, the regulator needs to decide whether to intervene. Node 4 remains liquid if it receives one intervention, or it will default and be included in the default set.

**Remark 2**. The notion of partial information is reflected by the fact that there are always unrevealed links before the process ends and the regulator cannot make decisions depending on the knowledge of it. The regulator can only be certain that if the remaining equity is nonpositive $\left(c_{k}^{\eta }\leq l_{k}^{\eta }\right)$ then the node *η* has defaulted at step *k*. On the other hand, every out-half-link from the default set represents a defaulted loan which is possible to impact every node in the network at a later time. So all the current liquid vulnerable nodes are subject to default at a later time and the regulator should take this into account when making intervention decisions at each step.

Let *R*_*k*_ be the accumulative number of interventions by step *k* and $D_{k}=\left|D_{k}\right|$ be the number of defaults at step *k*. The regulator aims to minimize the number of defaulted nodes by *T*_*n*_ with the minimum amount of interventions, so we define the objective function as a linear combination of the (scaled) number of interventions and defaults by the end of the process *T*_*n*_ as

$$\begin{array}{c} J_{n}=E\left(K\frac{R_{T_{n}}}{n}+\frac{D_{T_{n}}}{n}|G_{0}\right), \end{array}$$ (2)

where *K* > 0 is the relative “cost” of an intervention. Further by the definition of $c_{T_{n}}^{v}$ and noting that a node defaults at last if $c_{T_{n}}^{v}\leq l_{T_{n}}^{v}$, i.e. the number of defaulted loans exceeds the total of the initial equity level and the number of interventions received by *T*_*n*_, we can express $R_{T_{n}}$ and $D_{T_{n}}$ as

$$\begin{array}{cc} R_{T_{n}}= & \sum_{v\in [n]}\left(c_{T_{n}}^{v}-e\left(v\right)\right),\\ D_{T_{n}}= & \sum_{v\in [n]}1_{\left(c_{T_{n}}^{v}\leq l_{T_{n}}^{v}\right)}. \end{array}$$ (3)

Now we define the stochastic optimal control problem as

$$\begin{array}{c} \min_{\mu \in U}J_{n}, \end{array}$$ (4)

where *μ* = (*μ*_*k*_)_1≤*k* ≤ *m*_, *μ*_*k*_ ∈ {0, 1} and U contains all ${\left(G_{k}\right)}_{0\leq k\leq m}$ adapted process *μ*.

## The asymptotic control problem

### Assumptions and definitions

We assume that a bank cannot become liquid again once it has defaulted, thus we cannot save defaulted banks. This assumption is reasonable in the setting of default contagion in a stressed network and a short time window. Nor do we intervene on invulnerable nodes, because they never default but intervening on them will only prevent us from saving the banks that are very close to default especially when the interventions are costly.

In the model description we only intervene on the node that is selected at each step. Now we show that even if the regulator intervenes on multiple nodes and applies more than one unit of credit every time, it will not be better.

**Proposition 1**. For the stochastic control problem Eq (4), we only need to consider intervening on a node that, when selected, has only one unit of equity remaining.

We see proposition 1 implies that it is never optimal to intervene on a node if it is not selected or has more than one unit of equity remaining when selected. Let (*i*, *j*, *c*, *l*) be the state of a node, meaning it has the in and out degree (*i*, *j*), sum of the initial equity and the number of interventions *c* and *l* revealed in-links. Note that by definition *l* ≤ *i*. We characterize nodes with states because nodes with the same state have the same probability of being selected at each step and are statistically the same in influencing other nodes. Note in particular:

1. *c* = 0 denotes that the node has defaulted initially.
2. *c* − *l* denotes the remaining equity or “distance to default”, i.e. the number of times of being selected before a node defaults without interventions. Thus *c* ≤ *l* means the node has defaulted.
3. Because *l* ≤ *i* by definition, *i* < *c* implies that a node is invulnerable, i.e. even all loans lent out to the counterparties are written down from the balance sheet, the node still has positive remaining equity. On the contrary, 0 < *c* ≤ *i* denotes the node has the possibility to default, i.e. vulnerable.
4. In the beginning of the contagion process, all nodes have *l* = 0, i.e. are in states of the form (*i*, *j*, *c*, 0).

Then we define the state of the system at each step. Note that the number of nodes that have defaulted initially (*c* = 0) or invulnerable (*i* < *c*) in the beginning will not change throughout the process, so we only need to keep track of the nodes that are initially vulnerable (0 < *c* ≤ *i*) and currently liquid and if needed, we can always calculate the number of defaulted nodes at any time in the process. Further note that the possible states throughout the process for nodes that are vulnerable in the beginning and liquid at a later step are

$$\begin{array}{c} \Gamma ≔\{(i,j,c,l):0\leq i,0\leq j,0\leq l<c\leq i\;\text{or}\;c=i+1,l=i\}. \end{array}$$ (5)

Note particularly the state (*i*, *j*, *i* + 1, *i*) is the result that a node in state (*i*, *j*, *i*, *i* − 1) is selected and receives one intervention and thus becomes invulnerable.

**Definition 1**. (State variable *S*_*k*_) Let $S_{k}^{i,j,c,l}$ denote the number of nodes that are vulnerable initially and are in state (*i*, *j*, *c*, *l*) at step *k*, for *k* = 0, …, *m* and $S_{k}{≔(S_{k}^{i,j,c,l})}_{(i,j,c,l)\in \Gamma }$ be the state of the system. Note in the following we may use *α* to represent (*i*, *j*, *c*, *l*)∈Γ and write $S_{k}^{\alpha }$ instead of $S_{k}^{i,j,c,l}$ to simplify the notation.

Recall *m* = *m*(*n*) is the number of the total in (or out) degree of the network, which is also the maximum steps of the process. Throughout this paper we follow the convention that the superscript (usually a multi-index) denotes the state and the subscript denotes the time (discrete or continuous), e.g. $s_{\tau }^{i,j,c,l}$, $u_{\tau }^{i,j,c,c-1}$, $s_{t}^{i,j,c,l}$, $w_{t}^{i,j,c,l}$ and $u_{t}^{i,j,c,c-1}$ in the following. Then we define the empirical probability of in, out degrees and initial equity levels.

**Definition 2**. (Empirical probability) Define the empirical probability of the triplet (in degree, out degree, initial equity level) as

$$\begin{array}{c} P_{n}\left(i,j,c\right)=\frac{1}{n}|\{v\in \left[n\right]|d^{-}\left(v\right)=i,d^{+}\left(v\right)=j,e\left(v\right)=c\}|. \end{array}$$ (6)

Note that $\sum_{c\geq 0}P_{n}\left(i,j,c\right)=\frac{1}{n}|\{v\in \left[n\right]|d^{-}\left(v\right)=i,d^{+}\left(v\right)=j\}|$ represents the empirical probability of the in and out degree pair (*i*, *j*).

Previously we use *W*_*k*_ to denote the selected node at step *k*. Now with a little abuse of notation, let *W*_*k*_ denote the state of the selected node at step *k*, *k* = 1, …, *m*, so *W*_*k*_ ∈ Γ^+^ ≔ {(*i*, *j*, *c*, *l*):0 ≤ *i*, 0 ≤ *j*, 0 ≤ *c*, 0 ≤ *l* ≤ *i*}. We consider a Markovian control policy $G_{n}=\left(g_{1}^{\left(n\right)}\left(S_{0},W_{1}\right),\ldots ,g_{m}^{\left(n\right)}\left(S_{m-1},W_{m}\right)\right)$ where $g_{k+1}^{\left(n\right)}:N_{0}^{\left|\Gamma \right|}\times \Gamma^{+}\to \left\{0,1\right\}$ specifies the intervention at step *k* + 1 on the selected node which has state *W*_*k*+1_ given the state *S*_*k*_ and the superscript (*n*) shows the dependence on *n*.

Letting *P*_*n*_ = (*P*_*n*_(*i*, *j*, *c*))_*i*,*j*,0≤*c*≤*i*_, we rewrite the terms *J*_*n*_ as $J_{G_{n}}\left(P_{n}\right)$, $R_{T_{n}}$ as $R_{T_{n}}\left(G_{n},P_{n}\right)$ and $D_{T_{n}}$ as $D_{T_{n}}\left(G_{n},P_{n}\right)$ in Eq 4 based on *G*_*n*_ and *P*_*n*_, so

$$\begin{array}{cc} R_{T_{n}}\left(G_{n},P_{n}\right)= & \sum_{k=1}^{T_{n}}g_{k}^{\left(n\right)}\left(S_{k-1},W_{k}\right),\\ D_{T_{n}}\left(G_{n},P_{n}\right)= & n\sum_{i,j}P_{n}\left(i,j,0\right)+n\sum_{i,j,1\leq c\leq i}P_{n}\left(i,j,c\right)-\sum_{(i,j,c,l)\in \Gamma }S_{T_{n}}^{i,j,c,l}\\ = & n\sum_{i,j,0\leq c\leq i}P_{n}\left(i,j,c\right)-\sum_{(i,j,c,l)\in \Gamma }S_{T_{n}}^{i,j,c,l}. \end{array}$$ (7)

Note that the first equality for $D_{T_{n}}\left(G_{n},P_{n}\right)$ holds because the nodes that default at the end of the process consist of two parts: the nodes that have defaulted initially i.e. *n*∑_*i*,*j*_ *P*_*n*_(*i*, *j*, 0) and those nodes that are vulnerable initially and default during the process i.e. $n\sum_{i,j,1\leq c\leq i}P_{n}\left(i,j,c\right)-\sum_{(i,j,c,l)\in \Gamma }S_{T_{n}}^{i,j,c,l}$.

**Assumption 1**. Consider a sequence $\left(\left[n\right],E_{n}\right)$ of random networks, indexed by the size of the network *n*. For each n∈N, (*d*^−^(*v*))_*v*∈[*n*]_, (*d*^+^(*v*))_*v*∈[*n*]_ are sequences of nonnegative integers with ∑_*v*∈[*n*]_ *d*^−^(*v*) = ∑_*v*∈[*n*]_ *d*^+^(*v*) and such that for some probability distribution *p* on $N_{0}^{3}$ independent of *n* with λ ≔ ∑_*i*,*j*,*c*_ *ip*(*i*, *j*, *c*) = ∑_*i*,*j*,*c*_ *jp*(*i*, *j*, *c*)<∞, the following holds

1. *P*_*n*_(*i*, *j*, *c*) → *p*(*i*, *j*, *c*) ∀ *i*, *j*, *c* ≥ 0 as *n* → ∞.
2. ∑_*v*∈[*n*]_[(*d*^−^(*v*))^2^+ (*d*^+^(*v*))^2^] = *O*(*n*).

Note that the second assumption implies by uniform integrability that $\frac{m(n)}{n}\to \lambda$ as *n* → ∞ and recall that *m*(*n*) ≔ ∑_*v*∈[*n*]_ *d*^−^(*v*) = ∑_*v*∈[*n*]_ *d*^+^(*v*). Since *k* ≤ *m*(*n*), for large *n* it holds that $\frac{k}{n}\leq \frac{m(n)}{n}\leq \lambda +1$. Assumption 1 essentially implies the network is sparse which is justified in many empirical study literature on the structure of financial networks [24].

**Remark 3**. We previously defined that the vector *P*_*n*_ only includes *P*_*n*_(*i*, *j*, *c*) in the range 0 ≤ *c* ≤ *i*, i.e. the fractions of initially defaulted and vulnerable nodes. Accordingly define *p* ≔ (*p*(*i*, *j*, *c*))_*i*,*j*,0≤*c*≤*i*_, i.e. the vector *p* only includes *p*(*i*, *j*, *c*) in the range 0 ≤ *c* ≤ *i*.

Next we present our assumptions on the control functions $g_{k}^{\left(n\right)}$.

**Assumption 2**. Define Φ ≔ {(*i*, *j*, *c*, *c* − 1): 0 ≤ *i*, 0 ≤ *j*, 1 ≤ *c* ≤ *i*}. Let $G_{n}=\left(g_{1}^{\left(n\right)},\ldots ,g_{m}^{\left(n\right)}\right)$ be the a control policy (a sequence of control functions) for the contagion process on a network of size *n* where *n* is large enough such that $\frac{m(n)}{n}\leq \lambda +1$. Assume that

$$\begin{array}{c} g_{k+1}^{\left(n\right)}\left(s,w\right)=\{\begin{array}{cc} u_{\frac{k}{n}}^{i,j,c,c-1} & \text{if}\;w=(i,j,c,c-1)\in \Phi \\ 0 & \text{otherwise}, \end{array} \end{array}$$ (8)

for 0 ≤ *k* ≤ *m* − 1. $u_{\tau }^{i,j,c,c-1}=u^{i,j,c,c-1}\left(\tau \right)$ where *u*^*i*, *j*, *c*, *c*−1^: [0, λ + 1] → {0, 1} is a piecewise constant function on [0, λ + 1], i.e. there is a partition of the interval into a finite set of intervals such that *u*^*i*, *j*, *c*, *c*−1^ is constant 0 or 1 on each interval. Let *u* = (*u*^*β*^)_*β*∈Φ_ and Π contain all piecewise constant vector function *u* on [0, λ + 1].

Note that Φ includes possible states having the distance to default equal to one and Φ ⊂ #x0393;. Further $g_{k+1}^{\left(n\right)}\left(s,w\right)=0$ for *w* ∉ Φ follows from proposition 1. In the following we may use *β* to represent (*i*, *j*, *c*, *c* − 1) ∈ Φ and write $u_{\tau }^{\beta }$ instead of $u_{\tau }^{i,j,c,c-1}$ to simplify the notation.

**Remark 4**. By this assumption the function *u* is independent of the state but only a function of time. This implies that the control function $g_{k+1}^{\left(n\right)}\left(s,w\right)$ depends on the scaled time $\frac{k}{n}$ and the state of the currently selected node *w* but not on the state *s*. We will show it suffices to consider such control policy *G*_*n*_ later after proposition 3 because given a function *u*, we can predict a deterministic process to which the scaled stochastic contagion process converges in probability at any time as the size of the network *n* → ∞. Moreover, this type of control policies is the one that can be solved in the optimal control problem Eq (39) we will introduce later.

In summary, assumption 1 assumes the convergence of the empirical probabilities of the in and out degrees and the initial equity. On the other hand, proposition 3 indicates that the control functions depend on the scaled time and the state of the currently selected node. These two assumptions allow us to define the following asymptotic control problem by ensuring that the limits in the objective function are well defined.

**Definition 3**. For a sequence of networks with *P*_*n*_ and *G*_*n*_ satisfying assumption 1 and assumption 2, respectively, define the asymptotic control problem as

$$\begin{array}{cc} & \min_{u\in \Pi }\lim_{n\to \infty }J_{G_{n}}\left(P_{n}\right)\\ = & \min_{u\in \Pi }\lim_{n\to \infty }KE\frac{R_{T_{n}}\left(G_{n},P_{n}\right)}{n}+E\frac{D_{T_{n}}\left(G_{n},P_{n}\right)}{n}. \end{array}$$ (9)

In the following we will show the limits in Eq (9) are well defined by applying Wormald’s theorem [30].

### Dynamics of the default contagion process with interventions

Recall that *R*_*k*_ is the accumulative number of interventions up to step *k*, so

$$\begin{array}{cc} R_{0} & =0,\\ R_{k} & =\sum_{\ell =1}^{k}g_{\ell }\left(S_{\ell -1},W_{\ell }\right)\\ & =\sum_{\ell =1}^{k}\sum_{\beta \in \Phi }1_{\left(W_{\ell }=\beta \right)}u_{\frac{\ell -1}{n}}^{\beta }. \end{array}$$ (10)

**Fig 4:**
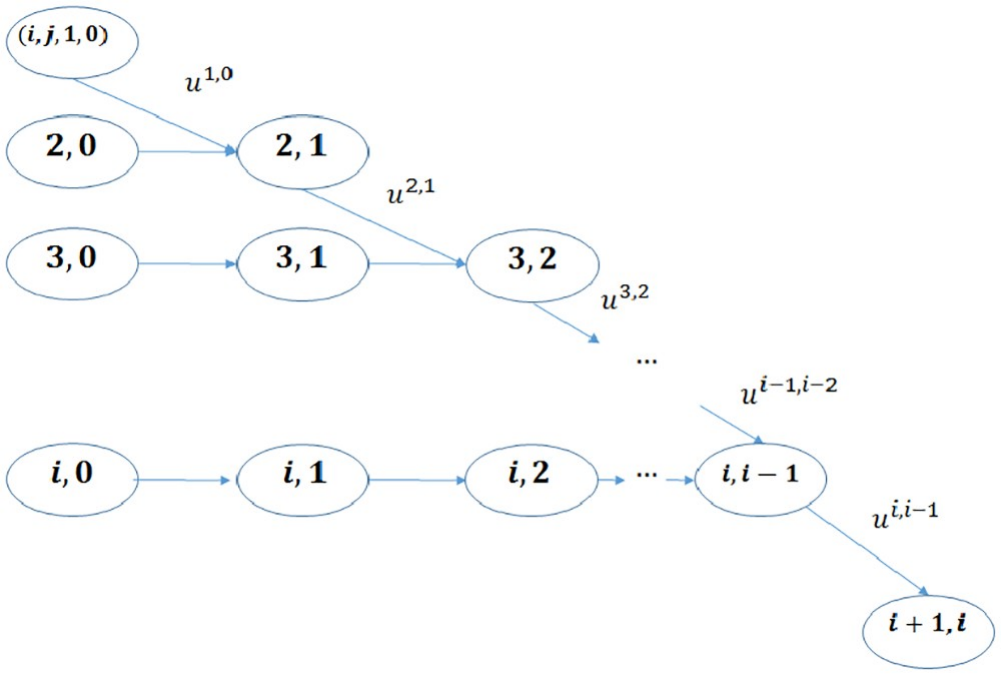
The state space for the same (*i*, *j*) pair, 0 ≤ *i*, 0 ≤ *j* and their transition relations.

We shall show that (*S*_*k*_, *R*_*k*_)_*k*=0, …, *m*_ is a controlled Markov chain given a control policy *G*_*n*_. In Fig 4 we illustrate for the same (*i*, *j*) pair the state space as well as their transition relations between the states.

To describe the transition probabilities, assume the state of the selected node at step *k* + 1 is *W*_*k*+1_ = (*i*, *j*, *c*, *l*), for *k* = 0, …, *m* − 1, there are three possibilities:

The selected node has defaulted, i.e. *c* ≤ *l* or the node is invulnerable, i.e. *c* > *i*, then *S*_*k*+1_ = *S*_*k*_, *R*_*k*+1_ = *R*_*k*_.

1. The selected node is vulnerable but has the “distance to default” greater than one, i.e. *c* − *l* ≥ 2, then the node *w* is selected with probability $\frac{\left(i-l\right)S_{k}^{i,j,c,l}}{m-k}$ and
  $$\begin{array}{cc} S_{k+1}^{i,j,c,l}= & S_{k}^{i,j,c,l}-1,\\ S_{k+1}^{i,j,c,l+1}= & S_{k}^{i,j,c,l+1}+1,\\ R_{k+1}= & R_{k}, \end{array}$$ (11)
  while other entries of *S*_*k*+1_ are the same as *S*_*k*_.
2. The selected node has the “distance to default” of one, i.e. *c* − *l* = 1, then the node is selected with probability $\frac{\left(i-c+1\right)S_{k}^{i,j,c,c-1}}{m-k}$ and by assumption 2,
  $$\begin{array}{cc} S_{k+1}^{i,j,c,c-1}= & S_{k}^{i,j,c,c-1}-1,\\ S_{k+1}^{i,j,c+1,c}= & S_{k}^{i,j,c+1,c}+g_{k+1}^{\left(n\right)}\left(S_{k},\left(i,j,c,c-1\right)\right)\\ = & S_{k}^{i,j,c+1,c}+u_{\frac{k}{n}}^{i,j,c,c-1},\\ R_{k+1}= & R_{k}+u_{\frac{k}{n}}^{i,j,c,c-1}, \end{array}$$ (12)
  while other entries of *S*_*k*+1_ are the same as *S*_*k*_.

Let ${\left(F_{k}\right)}_{k=0,\ldots ,m}$ be the natural filtration of *S*_*k*_, $\Delta S_{k}^{\alpha }=S_{k+1}^{\alpha }-S_{k}^{\alpha }$, *α* ∈ Γ and Δ*R*_*k*_ = *R*_*k*+1_ − *R*_*k*_, it follows that

$$\begin{array}{cc} E[\Delta S{}_{k}^{i,j,c,0}|F_{k}]= & -\frac{iS_{k}^{i,j,c,0}}{m-k}\;\text{for}\;1\leq c\leq i,\\ E[\Delta S_{k}^{i,j,c,l}|F_{k}]= & \frac{\left(i-l+1\right)S_{k}^{i,j,c,l-1}}{m-k}-\frac{\left(i-l\right)S_{k}^{i,j,c,l}}{m-k}\\ & \;\text{for}\;3\leq c\leq i,1\leq l\leq c-2,\\ E[\Delta S_{k}^{i,j,c,c-1}|F_{k}]= & \frac{\left(i-c+2\right)S_{k}^{i,j,c-1,c-2}}{m-k}u_{\frac{k}{n}}^{i,j,c-1,c-2}\\ & +\frac{\left(i-c+2\right)S_{k}^{i,j,c,c-2}}{m-k}-\frac{\left(i-c+1\right)S_{k}^{i,j,c,c-1}}{m-k}\\ & \;\text{for}\;2\leq c\leq i,\\ E[\Delta S_{k}^{i,j,i+1,i}|F_{k}]= & \frac{S_{k}^{i,j,i,i-1}}{m-k}u_{\frac{k}{n}}^{i,j,i,i-1},\\ E[\Delta R_{k}|F_{k}]= & \sum_{(i,j,c,c-1)\in \Phi }\frac{\left(i-c+1\right)S_{k}^{i,j,c,c-1}}{m-k}u_{\frac{k}{n}}^{i,j,c,c-1}. \end{array}$$ (13)

### Convergence of the default contagion process with interventions

Based on the dynamics of the contagion process under interventions described in the previous section, we will show that the state variable *S*_*k*_, the accumulative interventions *R*_*k*_, the number of defaults *D*_*k*_ and the number of unrevealed out-links from the default set $D_{k}^{-}$ (defined later) after being scaled by *n* all converge to a deterministic process which depends on the solution of the system of ODEs we will present now. Then we are able to show that the stochastic control problem Eq (4) converges to the asymptotic control problem Eq (9).

**Definition 4**. (ODEs of *s*_*τ*_) Given a set of piecewise constant function *u* = (*u*^*β*^)_*β*∈Φ_ on [0, λ], i.e. *u* ∈ *Π*, define the system of ordinary differential equations (ODEs) of $s_{\tau }={\left(s_{\tau }^{\alpha }\right)}_{\alpha \in \Gamma }$ as

$$\begin{array}{cc} \frac{ds_{\tau }^{i,j,c,0}}{d\tau }= & -\frac{is_{\tau }^{i,j,c,0}}{\lambda -\tau }\;\text{for}\;1\leq c\leq i,\\ \frac{ds_{\tau }^{i,j,c,l}}{d\tau }= & \frac{\left(i-l+1\right)s_{\tau }^{i,j,c,l-1}}{\lambda -\tau }-\frac{\left(i-l\right)s_{\tau }^{i,j,c,l}}{\lambda -\tau }\\ & \;\text{for}\;3\leq c\leq i,1\leq l\leq c-2,\\ \frac{ds_{\tau }^{i,j,c,c-1}}{d\tau }= & \frac{\left(i-c+2\right)s_{\tau }^{i,j,c-1,c-2}}{\lambda -\tau }u_{\tau }^{i,j,c-1,c-2}+\frac{\left(i-c+2\right)s_{\tau }^{i,j,c,c-2}}{\lambda -\tau }\\ & -\frac{\left(i-c+1\right)s_{\tau }^{i,j,c,c-1}}{\lambda -\tau }\;\text{for}\;2\leq c\leq i,\\ \frac{ds_{\tau }^{i,j,i+1,i}}{d\tau }= & \frac{s_{\tau }^{i,j,i,i-1}}{\lambda -\tau }u_{\tau }^{i,j,i,i-1}. \end{array}$$ (14)

The ODEs can be expressed in the form $\frac{ds_{\tau }}{d\tau }=h\left(\tau ,s_{\tau };u_{\tau }\right)$ where *h* = (*h*^*α*^)_*α*∈#x0393;_.

For what is needed below we analyze the solutions of the ODEs in definition 4 for a subinterval of [0, λ] on which *u*_*τ*_ is a constant vector function.

**Proposition 2**. Let $s_{\tau }={\left(s_{\tau }^{\alpha }\right)}_{\alpha \in \Gamma }$ satisfy the system of ordinary differential equations in definition 4 with the initial conditions $s_{\tau_{1}}=s_{1}≔{\left(s_{1}^{\alpha }\right)}_{\alpha \in \Gamma }$ and assume *u*_*τ*_ is a constant vector function *u*_*τ*_ = *b* ≔ (*b*^*β*^)_*β*∈Φ_ in the interval [*τ*_1_, *τ*_2_) ⊆ [0, λ) where *b*^*β*^ ∈ {0, 1} is a constant, then the solution *s*_*τ*_ on [*τ*_1_, *τ*_2_) is

$$\begin{array}{ll} s_{\tau }^{i,j,c,l}= & {\left(\frac{\lambda -\tau }{\lambda -\tau_{1}}\right)}^{i-l}\sum_{r=0}^{l}s_{1}^{i,j,c,r}\left(\begin{array}{c} i-r\\ l-r \end{array}\right){\left(1-\frac{\lambda -\tau }{\lambda -\tau_{1}}\right)}^{l-r}\\ & \text{for}\;2\leq c\leq i,0\leq l\leq c-2, \end{array}$$ (15)

$$\begin{array}{ll} s_{\tau }^{i,j,c,c-1}= & {\left(\frac{\lambda -\tau }{\lambda -\tau_{1}}\right)}^{i-c+1}\sum_{r=0}^{c-1}\sum_{q=r+1}^{c}\prod_{k=q}^{c-1}b^{i,j,k,k-1}s_{1}^{i,j,q,r}\left(\begin{array}{c} i-r\\ c-1-r \end{array}\right){\left(1-\frac{\lambda -\tau }{\lambda -\tau_{1}}\right)}^{c-1-r}\\ & \text{for}\;1\leq c\leq i, \end{array}$$ (16)

$$s_{\tau }^{i,j,i+1,i}=s_{1}^{i,j,i+1,i}+\sum_{r=0}^{i-1}\sum_{q=r+1}^{i}\prod_{k=q}^{i}b^{i,j,k,k-1}s_{1}^{i,j,q,r}{\left(1-\frac{\lambda -\tau }{\lambda -\tau_{1}}\right)}^{i-r},$$ (17)

where $\prod_{k=c}^{c-1}b^{i,j,k,k-1}≔1$. As a direct result, if we take the initial condition $s_{\tau_{1}}^{i,j,c,l}=p\left(i,j,c\right)1_{\left(l=0\right)}$ for (*i*, *j*, *c*, *l*)∈Γ at *τ*_1_ = 0, it follows that

sτi,j,c,l=p(i,j,c)(il)(1-τλ)i-l(τλ)lfor2≤c≤i,0≤l≤c-2. (18)

**Remark 5**. We discuss some properties of *s*_*τ*_. Observe that the ODEs are “separable” in that $s_{\tau }^{i,j,c,l}$ only depends on the entries of *s*_*τ*_ and *p* with the same (*i*, *j*). Fix an (*i*, *j*) pair, define Γ_*i*,*j*_ ≔ {(*c*, *l*):0 ≤ *l* < *c* ≤ *i* or *c* = *i* + 1, *l* = *i*}. If *u*_*τ*_ is a constant vector of 1’s on [*τ*_1_, *τ*_2_) ⊆ [0, λ), then we can show after some algebra that for any *τ* ∈ [*τ*_1_, *τ*_2_),

$$\begin{array}{c} \sum_{\left(c,l\right)\in \Gamma_{i,j}}s_{\tau }^{i,j,c,l}=\sum_{\left(c,l\right)\in \Gamma_{i,j}}s_{\tau_{1}}^{i,j,c,l}. \end{array}$$ (19)

If there exists some *c*_0_ such that 1 ≤ *c*_0_ ≤ *i*, $u_{\tau }^{i,j,c_{0},c_{0}-1}=0$, then $\sum_{\left(c,l\right)\in \Gamma_{i,j}}s_{\tau }^{i,j,c,l}<\sum_{\left(c,l\right)\in \Gamma_{i,j}}s_{\tau_{1}}^{i,j,c,l}$. Since the initial condition is $s_{0}^{i,j,c,l}=p\left(i,j,c\right)1_{\left(l=0\right)}$ for 1 ≤ *c* ≤ *i*, it follows that

$$\begin{array}{c} \sum_{\left(c,l\right)\in \Gamma_{i,j}}s_{\tau }^{i,j,c,l}\leq \sum_{1\leq c\leq i}p\left(i,j,c\right). \end{array}$$ (20)

In the following part our goal is to approximate $\frac{R_{k}}{n}$ and $\frac{D_{k}}{n}$ as *n* → ∞ given a function *u*. However, the number of variables depends on *n*, so we need to bound the terms associated with large in or out degrees. Fix *ϵ* > 0 and by assumption 1 we have that

$$\begin{array}{c} \lambda =\sum_{i,j,c}ip\left(i,j,c\right)=\sum_{i,j,c}jp\left(i,j,c\right)<\infty , \end{array}$$ (21)

then there exists an integer *M*^*ϵ*^ such that

$$\begin{array}{c} \sum_{i\geq M^{\epsilon }}\sum_{j,c}ip\left(i,j,c\right)+\sum_{j\geq M^{\epsilon }}\sum_{i,c}jp\left(i,j,c\right)<\epsilon , \end{array}$$ (22)

so letting *i* ∨ *j* = max{*i*, *j*}, we have

$$\begin{array}{cc} & \sum_{i\vee j\geq M^{\epsilon },c}jp\left(i,j,c\right)\\ = & \sum_{i\geq M^{\epsilon }}\sum_{j<M^{\epsilon }}\sum_{c}jp\left(i,j,c\right)+\sum_{i\geq M^{\epsilon }}\sum_{j\geq M^{\epsilon }}\sum_{c}jp\left(i,j,c\right)+\sum_{i<M^{\epsilon }}\sum_{j\geq M^{\epsilon }}\sum_{c}jp\left(i,j,c\right)\\ \leq & \sum_{i\geq M^{\epsilon }}\sum_{j<M^{\epsilon }}\sum_{c}ip\left(i,j,c\right)+\sum_{i\geq M^{\epsilon }}\sum_{j\geq M^{\epsilon }}\sum_{c}jp\left(i,j,c\right)+\sum_{i<M^{\epsilon }}\sum_{j\geq M^{\epsilon }}\sum_{c}jp\left(i,j,c\right)\\ < & \epsilon . \end{array}$$ (23)

We can prove similarly that there exists an integer *L*^*ϵ*^ such that ∑_*i*∨*j*≥*L*^*ϵ*^,*c*_ *ip*(*i*, *j*, *c*) < *ϵ*, but without loss of generality we write *M*^*ϵ*^ instead of *L*^*ϵ*^ in what follows. Moreover, by assumption 1, as *n* → ∞,

$$\begin{array}{c} \sum_{i,j,c}iP_{n}\left(i,j,c\right)=\sum_{i,j,c}jP_{n}\left(i,j,c\right)\to \lambda <\infty , \end{array}$$ (24)

so for *n* large enough, we can show that

$$\begin{array}{cc} \sum_{i\vee j\geq M^{\epsilon },c}jP_{n}\left(i,j,c\right) & <\epsilon ,\\ \sum_{i\vee j\geq M^{\epsilon },c}iP_{n}\left(i,j,c\right) & <\epsilon . \end{array}$$ (25)

So we define the integer *M*^*ϵ*^ formally.

**Definition 5**. Given any *ϵ* > 0, define *M*^*ϵ*^ as the integer such that

$$\begin{array}{cc} \sum_{i\vee j\geq M^{\epsilon },c}ip\left(i,j,c\right) & <\epsilon ,\\ \sum_{i\vee j\geq M^{\epsilon },c}jp\left(i,j,c\right) & <\epsilon . \end{array}$$ (26)

Accordingly, define

$$\begin{array}{cc} \Gamma^{\epsilon }≔ & \{\left(i,j,c,l\right):i\vee j<M^{\epsilon },0\leq l<c\leq i\;\text{or}\;c=i+1,l=i\},\\ \Phi^{\epsilon }≔ & \{\left(i,j,c,c-1\right):i\vee j<M^{\epsilon },0\leq c\leq i\},\\ \hat{\lambda }≔ & \lambda -\epsilon , \end{array}$$ (27)

where *a* ∨ *b* = max{*a*, *b*}.

Next we show that the scaled state variable *S*_*k*_ and *R*_*k*_ converges in probability to the solution of the ODEs in definition 4 given the function *u*. The difficulty in proving proposition 3 arises from the fact that the right sides of the ODEs for *s*_*τ*_ in definition 4 are discontinuous due to interventions so the auxiliary Wormald’s theorem in Appendix B: Wormald’s theorem is not applicable and needs to be adapted.

**Proposition 3**. Consider a sequence of networks with initial conditions (*P*_*n*_)_*n* ≥ 1_ satisfying assumption 1 and let (*G*_*n*_)_*n*≥1_ be the sequence of control policies for the contagion process on the sequence of networks and (*G*_*n*_)_*n*≥1_ satisfy assumption 2 with the function *u* = (*u*^*β*^)_*β*∈Φ^*ϵ*^_, then

$$\begin{array}{cc} \underset{0\leq k\leq n\hat{\lambda }}{\sup}\frac{S_{k}^{\alpha }}{n}-s_{\frac{k}{n}}^{\alpha }= & O(n^{-\frac{1}{4}}),\\ \underset{0\leq k\leq n\hat{\lambda }}{\sup}\frac{{\tilde{R}}_{k}}{n}-\tilde{r}{}_{\frac{k}{n}}= & O(n^{-\frac{1}{4}}), \end{array}$$ (28)

with probability $1-O(n^{\frac{1}{4}}\exp\left(-n^{\frac{1}{4}}\right))$ and *α* ∈ Γ^*ϵ*^, where $s_{\tau }={\left(s_{\tau }^{\alpha }\right)}_{\alpha \in \Gamma^{\in }}$ is the solution for the ODEs in definition 4 with the initial conditions $s_{0}^{i,j,c,l}=p\left(i,j,c\right)1_{\left(l=0\right)}$ and

$$\begin{array}{cc} {\tilde{R}}_{0} & =0,\\ {\tilde{R}}_{k} & =\sum_{\ell =1}^{k}\sum_{\beta \in \Phi^{\epsilon }}1_{\left(W_{\ell }=\beta \right)}u_{\frac{\ell -1}{n}}^{\beta }, \end{array}$$ (29)

and

$$\begin{array}{c} {\tilde{r}}_{\tau }=\int_{0}^{\tau }\sum_{\left(i,j,c,c-1\right)\in \Phi^{\epsilon }}\frac{\left(i-c+1\right)s_{t}^{i,j,c,c-1}}{\lambda -t}u_{t}^{i,j,c,c-1}dt. \end{array}$$ (30)

From proposition 3 we see that given (*P*_*n*_)_*n*≥1_ and (*G*_*n*_)_*n*≥1_ satisfying assumption 1 and assumption 2, respectively, the scaled stochastic process $\frac{S_{k}}{n}$ converges to the deterministic process $s_{\frac{k}{n}}$ for any *k* in $\left[0,n\hat{\lambda }\right]$. This justifies assumption 2 on the control policy because given a control policy *G*_*n*_ depending on the function *u*, we can predict with high probability the scaled stochastic contagion process at any time *k*.

Next we discuss the convergence of the scaled number of defaults $\frac{D_{k}}{n}$ and the process end time $\frac{T_{n}}{n}$. Note in definition 6, definition 7 and proposition 4 it is not required that *i* ∨ *j* < *M*^*ϵ*^

**Definition 6**. Define $D_{k}^{-}$ the number of unrevealed out links from the default set at step *k*.

Recall that *D*_*k*_ denotes the number of defaulted nodes at step *k* which consist of two parts: the nodes that have defaulted initially *n*∑_*i*,*j*_ *P*_*n*_(*i*, *j*, 0) and those that are vulnerable initially and default by step *k*, i.e. $n\sum_{i,j,1\leq c\leq i}P_{n}\left(i,j,c\right)-\sum_{(i,j,c,l)\in \Gamma }S_{k}^{i,j,c,l}$, thus

$$\begin{array}{cc} D_{k}= & n\sum_{i,j}P_{n}\left(i,j,0\right)+n\sum_{i,j,1\leq c\leq i}P_{n}\left(i,j,c\right)-\sum_{(i,j,c,l)\in \Gamma }S_{k}^{i,j,c,l}\\ = & n\sum_{i,j,0\leq c\leq i}P_{n}\left(i,j,c\right)-\sum_{(i,j,c,l)\in \Gamma }S_{k}^{i,j,c,l}. \end{array}$$ (31)

Similarly, among all defaulted nodes at step *k* the nodes with out degree *j* consist of two parts: the nodes that have defaulted initially *n*∑_*i*_ *P*_*n*_(*i*, *j*, 0) and those nodes that are vulnerable initially and default by step *k*, $n\sum_{i,1\leq c\leq i}P_{n}\left(i,j,c\right)-\sum_{i,0\leq l<c\leq i\;\text{or}\;c=i+1,l=i}S_{k}^{i,j,c,l}$, thus

$$\begin{array}{cc} D_{k}^{-}= & \sum_{j}j\left(n\sum_{i}P_{n}\left(i,j,0\right)+n\sum_{i,1\leq c\leq i}P_{n}\left(i,j,c\right)-\sum_{i,0\leq l<c\leq i\;\text{or}\;c=i+1,l=i}S_{k}^{i,j,c,l}\right)-k\\ = & n\sum_{i,j,0\leq c\leq i}jP_{n}\left(i,j,c\right)-\sum_{(i,j,c,l)\in \Gamma }jS_{k}^{i,j,c,l}-k. \end{array}$$ (32)

Correspondingly we make the following definitions to approximate $\frac{D_{k}}{n}$ and $\frac{D_{k}^{-}}{n}$ as *n* → ∞.

**Definition 7**. Define

$$\begin{array}{cc} d_{\tau }= & \sum_{i,j,0\leq c\leq i}p\left(i,j,c\right)-\sum_{(i,j,c,l)\in \Gamma }s_{\tau }^{i,j,c,l},\\ d_{\tau }^{-}= & \sum_{i,j,0\leq c\leq i}jp\left(i,j,c\right)-\sum_{(i,j,c,l)\in \Gamma }js_{\tau }^{i,j,c,l}-\tau . \end{array}$$ (33)

**Proposition 4**. Based on definition 6 and definition 7, it follows that

$$\begin{array}{cc} \underset{0\leq k\leq n\hat{\lambda }}{\sup}|\frac{D_{k}^{-}}{n}-d_{\frac{k}{n}}^{-}| & \leq o_{p}\left(1\right)+2\epsilon ,\\ \underset{0\leq k\leq n\hat{\lambda }}{\sup}|\frac{D_{k}}{n}-d_{\frac{k}{n}}| & \leq o_{p}\left(1\right)+2\epsilon . \end{array}$$ (34)

To summarize the results we have so far, we have shown in proposition 3 and proposition 4 that the state variable *S*_*k*_, the accumulative interventions *R*_*k*_, the number of defaults *D*_*k*_ and the number of unrevealed out-links from the default set $D_{k}^{-}$ after being scaled by *n* all converge to a deterministic process which depends on the solution of ODEs in definition 4. This convergence applies to any *k* before $n\hat{\lambda }$. By definition 6, $T_{n}=\min\left\{0\leq k\leq m:D_{k}^{-}=0\right\}$. Additionally define $\tau_{f}=\inf\left\{0\leq \tau \leq \lambda :d_{\tau }^{-}=0\right\}$. Next we show that when $\frac{T_{n}}{n}$ converges in probability to *τ*_*f*_, then $\frac{R_{T_{n}}}{n}$ and $\frac{D_{T_{n}}}{n}$ also converge in probability to the corresponding deterministic variables, $r_{\tau_{f}}$ and $d_{\tau_{f}}$, which in light of the boundedness of $\frac{R_{T_{n}}}{n}$ and $\frac{D_{T_{n}}}{n}$ further implies convergence in expectations, thus the limits in Eq (9) are well defined.

**Proposition 5**. Consider a sequence of networks with initial conditions (*P*_*n*_)_*n*≥1_ satisfying assumption 1 and let (*G*_*n*_)_*n*≥1_ be the sequence of control policies for the contagion processes on the sequence of networks and (*G*_*n*_)_*n*≥1_ satisfy assumption 2 with the function *u*. If *τ*_*f*_ = λ, or *τ*_*f*_ < λ and $\frac{d}{d\tau }d_{\tau_{f}}^{-}<0$, it follows that as *n* → ∞,

$$\begin{array}{cc} \frac{R_{T_{n}}\left(G_{n},P_{n}\right)}{n}\overset{p}{\to } & r_{\tau_{f}}\left(u,p\right),\\ \frac{D_{T_{n}}\left(G_{n},P_{n}\right)}{n}\overset{p}{\to } & d_{\tau_{f}}\left(u,p\right). \end{array}$$ (35)

where

$$\begin{array}{c} r_{\tau_{f}}=\int_{0}^{\tau_{f}}\sum_{(i,j,c,c-1)\in \Phi }\frac{\left(i-c+1\right)s_{t}^{i,j,c,c-1}}{\lambda -t}u_{t}^{i,j,c,c-1}dt. \end{array}$$ (36)

Further it follows that as *n* → ∞,

$$\begin{array}{cc} E\frac{R_{T_{n}}\left(G_{n},P_{n}\right)}{n}\to & r_{\tau_{f}}\left(u,p\right),\\ E\frac{D_{T_{n}}\left(G_{n},P_{n}\right)}{n}\to & d_{\tau_{f}}\left(u,p\right). \end{array}$$ (37)

Under the conditions in proposition 5, the asymptotic control problem Eq (9) becomes

$$\begin{array}{c} \min_{u\in \Pi }\;K\cdot r_{\tau_{f}}\left(u,p\right)+d_{\tau_{f}}\left(u,p\right). \end{array}$$ (38)

In the following let $u_{\tau }={\left(u_{\tau }^{\beta }\right)}_{\beta \in \Phi }$ and *u* = (*u*_*τ*_)_*τ* ∈ [0, λ]_.

Substituting the expressions of $r_{\tau_{f}}\left(u,p\right)$ and $d_{\tau_{f}}\left(u,p\right)$ in Eqs (36) and (33) respectively into Eq (38) and putting together the system of ordinary differential equations of $s_{\tau }={\left(s_{\tau }^{\alpha }\right)}_{\alpha \in \Gamma }$, i.e. $\frac{d}{d\tau }s_{\tau }=h\left(\tau ,s_{\tau };u_{\tau }\right)$ in definition 4 as well as the condition that determines *τ*_*f*_, i.e. $d_{\tau_{f}}^{-}=0$, we attain the following deterministic optimal control problem.

$$\begin{array}{cccc} \min_{u,\tau_{f}} & \; & K\cdot r_{\tau_{f}}\left(u,p\right) & +d_{\tau_{f}}\left(u,p\right)\\ \text{st} & & \;\frac{d}{d\tau }s_{\tau } & =h(\tau ,s_{\tau };u_{\tau })\\ & \; & s_{0}^{i,j,c,l} & =p\left(i,j,c\right)1_{\left(l=0\right)}\\ & \; & d_{\tau_{f}}^{-} & =0\\ & \; & u_{\tau }^{\beta } & \in \{0,1\}\;\forall \beta \in \Phi \\ & \; & \tau_{f} & \in [0,\lambda ), \end{array}$$ (39)

where $\frac{d}{d\tau }s_{\tau }=h\left(\tau ,s_{\tau };u_{\tau }\right)$ is defined as in definition 4 and

$$\begin{array}{cc} r_{\tau_{f}}\left(u,p\right) & =\int_{0}^{\tau_{f}}\sum_{i,j,1\leq c\leq i}\frac{\left(i-c+1\right)s_{\tau }^{i,j,c,c-1}}{\lambda -\tau }u_{\tau }^{i,j,c,c-1}d\tau ,\\ d_{\tau_{f}}\left(u,p\right) & =\sum_{i,j,0\leq c\leq i}p\left(i,j,c\right)-\sum_{(i,j,c,l)\in \Gamma }s_{\tau_{f}}^{i,j,c,l},\\ d_{\tau_{f}}^{-} & =\sum_{i,j,0\leq c\leq i}jp\left(i,j,c\right)-\sum_{(i,j,c,l)\in \Gamma }js_{\tau_{f}}^{i,j,c,l}-\tau_{f}. \end{array}$$ (40)

Some difficulties arise because Eq (39) is an infinite dimensional optimal control problem. In light of assumption 1, it suffices to solve a finite dimensional problem to approximate the objective function of the infinite dimensional problem. First we define the finite dimensional optimal control problem.

**Definition 8**. (FOCP) For *ϵ* > 0, recall *M*^*ϵ*^ as in definition 5. Define the finite dimensional optimal control problem (FOCP) as Eq (39) with the indexes (*i*, *j*) restricted to *i* ∨ *j* < *M*^*ϵ*^.

**Remark 6**. The restriction of (*i*, *j*) to *i* ∨ *j* < *M*^*ϵ*^ indicates that we use only *p*(*i*, *j*, *c*), *i* ∨ *j* < *M*^*ϵ*^, 0 ≤ *c* ≤ *i* in the calculation. It is equivalent to setting *p*(*i*, *j*, *c*) = 0 for *i* ∨ *j* ≥ *M*^*ϵ*^, 0 ≤ *c* ≤ *i* while keeping *p*(*i*, *j*, *c*) for *i* ∨ *j* < *M*^*ϵ*^, 0 ≤ *c* ≤ *i* unchanged, which implies asymptotically nodes with *i* ∨ *j* ≥ *M*^*ϵ*^ are all invulnerable. By the solution of the ODE in proposition 2, it implies that $s_{\tau }^{\alpha }=0$, for *α* ∈ Γ∖Γ^*ϵ*^. Note we use tilde sign with the variables to indicate the indexes (*i*, *j*) are in the range *i* ∨ *j* < *M*^*ϵ*^, for example, ${\tilde{r}}_{\tau_{f}}$, ${\tilde{d}}_{\tau_{f}}$ and ${\tilde{d}}_{\tau_{f}}^{-}$.

We have the following lemma regarding the objective functions of the infinite and finite dimensional optimal control problems.

**Lemma 1**. Let $\zeta \left(u,\tau_{f},p\right)≔K\;r_{\tau_{f}}\left(u,p\right)+d_{\tau_{f}}\left(u,p\right)$ be the objective function for the infinite dimensional Eq (39) and $\tilde{\zeta }\left(u,\tau_{f},p\right)≔K\;\tilde{r}{}_{\tau_{f}}\left(u,p\right)+{\tilde{d}}_{\tau_{f}}\left(u,p\right)$ for (FOCP). Let $\left(u^{*},\tau_{f}^{*}\right)$ and $\left(\tilde{u},{\tilde{\tau }}_{f}\right)$ be the optimal solutions for the infinite dimensional Eq (39) and (FOCP), respectively, then for the same *p* we have that

$$\begin{array}{c} |\tilde{\zeta }\left(\tilde{u},{\tilde{\tau }}_{f},p\right)-\zeta \left(u^{*},\tau_{f}^{*},p\right)|<\left(K+1\right)\epsilon . \end{array}$$ (41)

By lemma 1 we only need to solve the finite dimensional optimal control problem (FOCP) in definition 8. Because *ϵ* can be arbitrarily small, we can approximate the objective function of the infinite dimensional problem to any precision. Given *p* for (FOCP), the Pontryagin’s maximum principle provides the necessary conditions for the optimal control $\tilde{u}$ and end time ${\tilde{\tau }}_{f}$. We can obtain the optimal asymptotic number of interventions $\tilde{r}{}_{{\tilde{\tau }}_{f}}$ and fraction of final defaults $\tilde{d}{}_{{\tilde{\tau }}_{f}}$, which lead to the main results of our work. In the next section we focus on solving (FOCP) and suppress the tilde sign for the variables for notational convenience.

### Necessary conditions for the optimal control problem

In the following we solve the finite dimensional optimal control problem (FOCP) in definition 8. Throughout this section we understand that the degrees are in the bounded range *i* ∨ *j* < *M*^*ϵ*^ unless specified otherwise. We also suppress the tilde sign for notational convenience. Let *t* = *t*(*τ*) ≔ − ln(λ − *τ*), *t*_0_ ≔ *t*(0) = −ln λ and *t*_*f*_ ≔ −ln(λ − *τ*_*f*_). Note *t*(*τ*) is a strictly increasing function of *τ* and so is the inverse function *τ* = *τ*(*t*). We remark that we assume in the following that *τ* < λ which implies *t*_*f*_ < ∞, but later we can see that the solutions of *s*_*τ*_, *u*_*τ*_ and *w*_*τ*_ do allow *τ* = λ. Then we can reformulate the optimal control problem Eq (39) into an autonomous one, i.e. the differential equations of the system dynamics do not depend on time explicitly. Let $u_{t}={\left(u_{t}^{\beta }\right)}_{\beta \in \Phi^{\in }}$ and $u={\left(u_{t}\right)}_{t\geq t_{0}}$ (note previously *u* = (*u*_*τ*_)_*τ*∈[0,λ]_. Additionally, we allow an arbitrary starting time *t*_0_ here).

$$\begin{array}{cc} & \left(42\right)\\ \begin{array}{cccc} \underset{u,t_{f}}{\min} & \; & K\cdot r_{t_{f}}\left(u,p\right) & +d_{t_{f}}\left(u,p\right)\\ \text{st} & \; & \frac{d}{dt}s_{t} & =h\left(s_{t};u_{t}\right)\\ & \; & s_{t_{0}}^{i,j,c,l} & =p\left(i,j,c\right)1_{\left(l=0\right)}\\ & \; & d_{t_{f}}^{-} & =0\\ & \; & u_{t}^{\beta } & \in \left\{0,1\right\},\;\forall \beta \in \Phi^{\epsilon }\\ & \; & t_{f} & \in \left[0,\infty \right), \end{array} & \left(43\right) \end{array}$$

where $\frac{d}{dt}s_{t}=h\left(s_{t};u_{t}\right)$ denotes the system of differential equations

$$\begin{array}{cc} \frac{ds_{t}^{i,j,c,0}}{dt}= & -is_{t}^{i,j,c,0}\;\text{for}\;1\leq c\leq i,\\ \frac{ds_{t}^{i,j,c,l}}{dt}= & \left(i-l+1\right)s_{t}^{i,j,c,l-1}-\left(i-l\right)s_{t}^{i,j,c,l}\\ & \;\text{for}\;3\leq c\leq i,\;1\leq l\leq c-2,\\ \frac{ds_{t}^{i,j,c,c-1}}{dt}= & \left(i-c+2\right)s_{t}^{i,j,c-1,c-2}u_{t}^{i,j,c-1,c-2}+\left(i-c+2\right)s_{t}^{i,j,c,c-2}\\ & -\left(i-c+1\right)s_{t}^{i,j,c,c-1}\\ & \;\text{for}\;2\leq c\leq i,\\ \frac{ds_{t}^{i,j,i+1,i}}{dt}= & s_{t}^{i,j,i,i-1}u_{t}^{i,j,i,i-1}, \end{array}$$ (44)

and

$$\begin{array}{cc} r_{t_{f}}\left(u,p\right) & =\int_{t_{0}}^{t_{f}}\sum_{i,j,1\leq c\leq i}\left(i-c+1\right)s_{t}^{i,j,c,c-1}u_{t}^{i,j,c,c-1}dt, \end{array}$$ (45)

$$\begin{array}{cc} d_{t_{f}}\left(u,p\right) & =\sum_{i,j,0\leq c\leq i}p\left(i,j,c\right)-\sum_{\left(i,j,c,l\right)\in \Gamma^{\epsilon }}s_{t_{f}}^{i,j,c,l}, \end{array}$$ (46)

$$\begin{array}{cc} d_{t_{f}}^{-} & =\sum_{i,j,0\leq c\leq i}jp\left(i,j,c\right)-\sum_{\left(i,j,c,l\right)\in \Gamma^{\epsilon }}js_{t_{f}}^{i,j,c,l}-\tau_{f}\\ & =\sum_{i,j,0\leq c\leq i}jp\left(i,j,c\right)-\sum_{\left(i,j,c,l\right)\in \Gamma^{\epsilon }}js_{t_{f}}^{i,j,c,l}-\lambda \left(1-e^{t_{0}-t_{f}}\right). \end{array}$$ (47)

Note that Eq (47) follows from $\frac{1}{\lambda }=e^{t_{0}}$ and thus $\tau_{f}=\lambda -e^{-t_{f}}=\lambda \left(1-\frac{1}{\lambda }e^{-t_{f}}\right)=\lambda \left(1-e^{t_{0}-t_{f}}\right)$.

To determine the necessary conditions for the optimal terminal time $t_{f}^{*}$ and optimal control $u_{t}^{*}$ in Eq (42), we need the Extended Pontryagin Maximum Principle in Appendix C: Extended pontryagin maximum principle. Then we put together the objective function Eq (43) and the necessary conditions to construct the optimization problem Eq (86) we will introduce later.

Applying the Extended Pontryagin Maximum Principle to the optimal control problem Eq (42) yields the following necessary conditions of optimality. Note in order to simplify notations, we suppress the apostrophe “*” in the following. In other words, we use *s*_*t*_, *u*_*t*_, *w*_*t*_, *v*, *t*_*f*_ instead of $s_{t}^{*},u_{t}^{*},w_{t}^{*},v^{*},t_{f}^{*}$ to denote the optimal values.

**Proposition 6**. (Necessary conditions of optimality) Let (*s*_*t*_, *u*_*t*_)_*t*∈[*t*_0_, *t*_*f*_]_ be the optimal state and control trajectory of Eq (42) where *t*_*f*_ is the optimal terminal time. Define the Hamiltonian function

$$\begin{array}{cc} H\left(s_{t},u_{t},w_{t}\right)= & \sum_{i,j,1\leq c\leq i}w_{t}^{i,j,c,0}\left(-is_{t}^{i,j,c,0}\right)\\ & +\sum_{i,j,2\leq c\leq i,1\leq l\leq c-1}w_{t}^{i,j,c,l}[\left(i-l+1\right)s_{t}^{i,j,c,l-1}-\left(i-l\right)s_{t}^{i,j,c,l}]\\ & +\sum_{i,j,2\leq c\leq i+1}\left(K+w_{t}^{i,j,c,c-1}\right)\left(i-c+2\right)s_{t}^{i,j,c-1,c-2}u_{t}^{i,j,c-1,c-2}, \end{array}$$ (48)

then there exists a piecewise continuously differentiable vector function $w_{t}={\left(w_{t}^{\alpha }\right)}_{\alpha \in \Gamma^{\in }}\in {\hat{C}}^{1}{\left[t_{0},\infty \right)}^{|\Gamma^{\in }|}$ and a scalar v∈R such that the following conditions hold:

1. The optimal control *u*_*t*_ satisfies that ∀*t* ∈ [*t*_0_, *t*_*f*_], 1 ≤ *c* ≤ *i*, if $s_{t}^{i,j,c,c-1}>0$,
  $$\begin{array}{cc} u_{t}^{i,j,c,c-1} & =\{\begin{array}{cc} 0 & \;\text{if}\;w_{t}^{i,j,c+1,c}>-K\\ 1 & \;\text{if}\;w_{t}^{i,j,c+1,c}<-K\\ 0\;\text{or}\;1 & \;\text{if}\;w_{t}^{i,j,c+1,c}=-K, \end{array} \end{array}$$ (49)
  if $s_{t}^{i,j,c,c-1}=0$,
  $$\begin{array}{cc} u_{t}^{i,j,c,c-1} & =0\;\text{or}\;1. \end{array}$$ (50)
2. For 2 ≤ *c* ≤ *i*, 0 ≤ *l* ≤ *c* − 2,
  $$\begin{array}{c} \frac{d}{dt}w_{t}^{i,j,c,l}=\left(i-l\right)\left(w_{t}^{i,j,c,l}-w_{t}^{i,j,c,l+1}\right), \end{array}$$ (51)
  and for 1 ≤ *c* ≤ *i*,
  $$\begin{array}{c} \frac{d}{dt}w_{t}^{i,j,c,c-1}=\left(i-c+1\right)\left(w_{t}^{i,j,c,c-1}-\left(K+w_{t}^{i,j,c+1,c}\right)u_{t}^{i,j,c,c-1}\right), \end{array}$$ (52)
  and
  $$\begin{array}{c} \frac{d}{dt}w_{t}^{i,j,i+1,i}=0. \end{array}$$ (53)
  We denote the set of ordinary differential equations for *w*_*t*_ as $\frac{d}{dt}w_{t}=h^{\prime }\left(w_{t};u_{t}\right)$.
3. $H(s_{t},u_{t},w_{t})$ is a constant for *t* ∈ [*t*_0_, *t*_*f*_].
4. Transversal conditions
  $$\begin{array}{cc} w_{t_{f}}^{i,j,c,l} & =vj-1\;\forall \left(i,j,c,l\right)\in \Gamma^{\epsilon }, \end{array}$$ (54)
  $$\begin{array}{cc} H(s_{t_{f}},u_{t_{f}},w_{t_{f}}) & =-ve^{-t_{f}}, \end{array}$$ (55)
  $$\begin{array}{cc} d_{t_{f}}^{-} & =\sum_{i,j,0\leq c\leq i}jp\left(i,j,c\right)-\sum_{\left(i,j,c,l\right)\in \Gamma^{\epsilon }}js_{t_{f}}^{i,j,c,l}-\lambda \left(1-e^{t_{0}-t_{f}}\right)\\ & =0. \end{array}$$ (56)

**Remark 7**. (Singular control policy) Observe that if the coefficient of $u_{t}^{i,j,c,c-1}$ in the Hamiltonian function $H(s_{t},u_{t},w_{t})$ Eq (48), i.e. $\left(i-c+1\right)\left(K+w_{t}^{i,j,c+1,c}\right)s_{t}^{i,j,c,c-1}$ vanishes, $u_{t}^{i,j,c,c-1}=0$ or 1 both satisfy conditions of the Extended Pontryagin Maximum Principle in Appendix C: Extended pontryagin maximum principle i.e. minimizing $H(s_{t},u_{t},w_{t})$. In other words, the Extended Pontryagin Maximum Principle cannot determine the optimal control $u_{t}^{i,j,c,c-1}$ in this case. Moreover, since *i* − *c* + 1 > 0, so if $\left(K+w_{t}^{i,j,c+1,c}\right)s_{t}^{i,j,c,c-1}=0$ can be sustained over some interval (*θ*_1_, *θ*_2_) ⊂ [*t*_0_, *t*_*f*_], then $u_{t}^{i,j,c,c-1}$ can be 0 or 1 at any time on (*θ*_1_, *θ*_2_) and switch arbitrarily often between 0 and 1. In the terminology of optimal control theory, the control *u*_*t*_ is “singular” on (*θ*_1_, *θ*_2_) and the corresponding portion of the state trajectory *s*_*t*_ on (*θ*_1_, *θ*_2_) is called a singular arc. Further note that $\left(K+w_{t}^{i,j,c+1,c}\right)s_{t}^{i,j,c,c-1}=0$, *t* ∈ (*θ*_1_, *θ*_2_) implies two cases: $s_{t}^{i,j,c,c-1}=0$ or $s_{t}^{i,j,c,c-1}>0$, $w_{t}^{i,j,c+1,c}=-K$, *t* ∈ (*θ*_1_, *θ*_2_). We can show that in the first case any feasible control function $u_{t}^{i,j,c,c-1}$ will not affect other entries of *s*_*t*_ and the second case only occurs when *c* = *i* and (*θ*_1_, *θ*_2_) = (*t*_0_, *t*_*f*_).

### Solutions of the necessary conditions

Throughout this section we understand that the degrees are in the bounded range *i* ∨ *j* < *M*^*ϵ*^ unless specified otherwise. A well known fact in the control theorists community is that solving for the optimal (*s*_*t*_, *u*_*t*_, *w*_*t*_) from the necessary conditions presented in proposition 6 is difficult, especially analytically. In what follows, we solve for the optimal (*s*_*t*_, *u*_*t*_, *w*_*t*_) in three steps.

First, solve the the two-point boundary value problem (TPBVP) consisting of the differential equations for *s*_*t*_ in Eq (42) and *w*_*t*_ in condition (2) of proposition 6 where for *s*_*t*_ the boundary values are given at *t* = *t*_0_ and for *w*_*t*_ at *t* = *t*_*f*_ as follows.

$$\begin{array}{l} \begin{array}{ccc} \frac{d}{dt}s_{t} & =h(s_{t};u_{t}), & \\ s_{t_{0}}^{i,j,c,l} & =p(i,j,c)1_{\left(l=0\right)} & \forall (i,j,c,l)\in \Gamma^{\epsilon },\\ \frac{d}{dt}w_{t} & =h^{\prime }(w_{t};u_{t}), & \\ w_{t_{f}}^{i,j,c,l} & =vj-1\;\forall (i,j,c,l)\in \Gamma^{\epsilon }, & \end{array} \end{array}$$ (57)

and additionally the optimal control policy *u*_*t*_ satisfies Eq (50) in condition 1 of proposition 6. We solve for (*s*_*t*_, *u*_*t*_, *w*_*t*_) in terms of the auxiliary variables (*v*, *t*_*f*_, *t*_*s*_) and in the following we call these expressions in terms of (*v*, *t*_*f*_, *t*_*s*_) as the solutions of (*s*_*t*_, *u*_*t*_, *w*_*t*_).

Second, because the optimal (*s*_*t*_, *u*_*t*_, *w*_*t*_) satisfies the two equations in the necessary conditions of proposition 6, i.e. the Hamiltonian function Eq (55) at *t* = *t*_*f*_ and the terminal condition Eq (56), while minimizing the objective function Eq (43), we have the following optimization problem for (*s*_*t*_, *u*_*t*_, *w*_*t*_):

$$\begin{array}{lll} & & \left(58\right)\\ \underset{s_{t},u_{t},w_{t}}{\text{min}} & K\cdot it_{t_{f}}\left(u,p\right)+d_{t_{f}}\left(u,p\right) & \left(59\right)\\ \text{st} & H\left(s_{t_{f}},u_{t_{f}},w_{t_{f}}\right)=-ve^{-t_{f}} & \left(60\right)\\ & d_{t_{f}}^{-}=0, & \left(61\right) \end{array}$$

where

$$\begin{array}{cc} r_{t_{f}}\left(u,p\right) & =\int_{t_{0}}^{t_{f}}\sum_{i,j,1\leq c\leq i}\left(i-c+1\right)s_{t}^{i,j,c,c-1}u_{t}^{i,j,c,c-1}dt,\\ d_{t_{f}}\left(u,p\right) & =\sum_{i,j,0\leq c\leq i}p\left(i,j,c\right)-\sum_{\left(i,j,c,l\right)\in \Gamma^{\epsilon }}s_{t_{f}}^{i,j,c,l},\\ d_{t_{f}}^{-} & =\sum_{i,j,0\leq c\leq i}jp\left(i,j,c\right)-\sum_{\left(i,j,c,l\right)\in \Gamma^{\epsilon }}js_{t_{f}}^{i,j,c,l}-\lambda \left(1-e^{t_{0}-t_{f}}\right). \end{array}$$ (62)

After substituting (*s*_*t*_, *u*_*t*_, *w*_*t*_) expressed in terms of (*v*, *t*_*f*_, *t*_*s*_) into the optimization problem (58), we are able to solve the optimal (*v*, *t*_*f*_, *t*_*s*_) based on which we can calculate the optimal (*s*_*t*_, *u*_*t*_, *w*_*t*_).

Third, we change the variables from (*v*, *t*_*f*_, *t*_*s*_) to (*v*, *y*, *z*) for further simplification and attain the optimization problem Eq (86) later.

Now we carry out the first step. The main difficulty of solving problem Eq (57) comes from the fact that *w*_*t*_ and *s*_*t*_ depend on *u*_*t*_ which depends on *w*_*t*_ and *s*_*t*_ recursively through Eq (50). To disentangle the recursive dependence, the idea is to analyze the properties of *s*_*t*_ based on which we can either derive the properties of *w*_*t*_ or the explicit solutions of *w*_*t*_ depending on signs of *vj* − 1 + *K*. Then by Eq (50) we attain the optimal control policy *u*_*t*_ which leads to the solution of *s*_*t*_.

It turns out that we only need $w_{t_{f}}$ as well as *u*_*t*_ and *s*_*t*_ in (58). From problem Eq (57) we know that $w_{t_{f}}^{\beta }=vj-1$ for *β* ∈ Γ^*ϵ*^. For *u*_*t*_ and *s*_*t*_, we give out their solutions in the following directly due to the limited space of the paper. The solutions of *u*_*t*_ and *s*_*t*_ can be verified by substituting into problem Eq (57).

**Lemma 2**. The optimal control policy *u*_*t*_ in terms of the variables (*v*, *t*_*f*_, *t*_*s*_) is given as below.

For 1 ≤ *c* ≤ *i* except *c* = *i* and *vj* − 1 = −*K*, ∀*t* ∈ [*t*_0_, *t*_*f*_],

$$\begin{array}{c} u_{t}^{i,j,c,c-1}=1_{\left(t\geq t^{i,j,c}\right)}, \end{array}$$ (63)

where

$$\begin{array}{c} t^{i,j,c}=\{\begin{array}{cc} t_{f} & \;\text{if}\;vj-1\geq -K\\ t_{f}+\ln(1+\frac{K+vj-1}{(i-c)K}) & \;\text{if}\;vj-1<-K\;\text{and}\;1\leq c<i+\frac{K+vj-1}{K(1-e^{t_{0}-t_{f}})}\\ t_{0} & \text{otherwise}, \end{array} \end{array}$$ (64)

If *vj* − 1 = −*K*, ∀*t* ∈ [*t*_0_, *t*_*f*_],

$$\begin{array}{c} u_{t}^{i,j,i,i-1}=1_{\left(t\geq t_{s}\right)}\;\text{for}\;\text{some}\;t_{s}\in \left[t_{0},t_{f}\right]. \end{array}$$ (65)

The following is the solution for *s*_*t*_.

**Lemma 3**. Letting *p*(*i*, *j*, *i* + 1) = 0, under the optimal control policy in lemma 2, we have the following solutions of *s*_*t*_ for the two-point boundary value problem (TPBVP).

1. For 2 ≤ *c* ≤ *i*, 0 ≤ *l* ≤ *c* − 2 and *c* = 1, *l* = 0,
  sti,j,c,l=p(i,j,c)(il)(et0-t)i-l(1-et0-t)l. (66)
2. If *vj* − 1 < −*K*, consider *t* ∈ [*t*^*i*, *j*, *h*^, *t*^*i*, *j*, *h*−1^), for some 1 ≤ *h* ≤ *i* where
  $$\begin{array}{c} t^{i,j,h}=\{\begin{array}{cc} t_{f} & \text{if}\;h=0\\ t_{f}+\ln\left(1+\frac{K+vj-1}{(i-h)K}\right) & \text{if}\;1\leq h<i+\frac{K+vj-1}{K(1-e^{t_{0}-t_{f}})}\\ t_{0} & \text{otherwise}. \end{array} \end{array}$$ (67)
  If 1 ≤ *c* < *h*,
  sti,j,c,c-1=p(i,j,c)(ic-1)(et0-t)i-c+1(1-et0-t)c-1. (68)
  If *h* ≤ *c* ≤ *i* + 1,
  sti,j,c,c-1=(ic-1)(et0-t)i-c+1∑m=hcp(i,j,m)∑n=0m-1(c-1n)(1-et0-ti,j,m)n(et0-ti,j,m-et0-t)c-1-n. (69)
3. If *vj* − 1 > −*K*, for 1 ≤ *c* ≤ *i* + 1, *t* ∈ [*t*_0_, *t*_*f*_],
  sti,j,c,c-1=p(i,j,c)(ic-1)(et0-t)i-c+1(1-et0-t)c-1. (70)
4. If *vj* − 1 = −*K*, for 1 ≤ *c* ≤ *i*, *t* ∈ [*t*_0_, *t*_*f*_],
  sti,j,c,c-1=p(i,j,c)(ic-1)(et0-t)i-c+1(1-et0-t)c-1, (71)
  and
  $$\begin{array}{c} s_{t}^{i,j,i+1,i}=p\left(i,j,i\right)1_{\left\{t_{s}\leq t\right)}\left[{\left(1-e^{t_{0}-t}\right)}^{i}-{\left(1-e^{t_{0}-t_{s}}\right)}^{i}\right], \end{array}$$ (72)
  where *t*_*s*_ ∈ [*t*_0_, *t*_*f*_].

Since Eqs (56) and (55) require the state variable value particularly at *t* = *t*_*f*_, we can apply lemma 3 at *t* = *t*_*f*_ to attain $s_{t_{f}}$. Next we proceed to the second step, i.e. we substitute $\left(s_{t},u_{t},w_{t_{f}}\right)$ in terms of (*v*, *t*_*f*_, *t*_*s*_) into the optimization program (58) which leads to the following results.

**Proposition 7**. Based on the solutions of optimal *s*_*t*_ in lemma 3 (particularly $s_{t_{f}}$), *u*_*t*_ in lemma 2 and $w_{t_{f}}^{\alpha }=vj-1$, ∀*α* ∈ Γ^*ϵ*^, letting

$$\begin{array}{c} t^{i,j,c}=\{\begin{array}{cc} t_{f} & \text{if}\;K+vj-1\geq 0\;\text{or}\;c=0\\ t_{f}+\ln\left(1+\frac{K+vj-1}{(i-c)K}\right) & \text{if}\;K+vj-1<0\;\text{and}\;1\leq c<i+\frac{K+vj-1}{K(1-e^{t_{0}-t_{f}})},\\ t_{0} & \text{otherwise}, \end{array} \end{array}$$ (73)

the Hamiltonian equation Eq (60) at *t* = *t*_*f*_ is equivalent to

∑jmax{-K,vj-1}∑ii∑c=1ip(i,j,c)∑m=ci(i-1m-1)(et0-tf)i-m+1∑n=0c-1(m-1n)(1-et0-ti,j,c)n(et0-ti,j,c-et0-tf)m-1-n=vλet0-tf. (74)

The terminal condition Eq (61) is equivalent to

∑i∑jj{∑c=0ip(i,j,c)∑n=ci(in)(1-et0-ti,j,c)n(et0-ti,j,c)i-n-1(vj-1=-K)p(i,j,i)[(1-et0-tf)i-(1-et0-ts)i]}=λ(1-et0-tf). (75)

And the objective function Eq (59) becomes

K·rtf(u,p)+dtf(u,p)=K∑i∑j{∑c=1ip(i,j,c)∑m=ci∑n=0c-1(m-c+1)(im)(mn)(et0-tf)i-m(1-et0-ti,j,c)n(et0-ti,j,c-et0-tf)m-n+1(vj-1=-K)p(i,j,i)[(1-et0-tf)i-(1-et0-ts)i]}+∑j∑i{∑c=0ip(i,j,c)∑n=ci(in)(1-et0-ti,j,c)n(et0-ti,j,c)i-n-1(vj-1=-K)p(i,j,i)[(1-et0-tf)i-(1-et0-ts)i]}. (76)

For the third step, we further simplify the expressions in proposition 7. Define

$$\begin{array}{cc} y & ≔1-e^{t_{0}-t_{f}}=\frac{\tau_{f}}{\lambda },\\ z & ≔1-e^{t_{0}-t_{s}},\\ x^{i,j,c,c-1} & ≔1-e^{t_{0}-t^{i,j,c}}\\ & =\{\begin{array}{cc} y & \text{if}\;K+vj-1\geq 0\;\text{or}\;c=0\\ 1-\left(1-y\right)\frac{(i-c)K}{(i-c+1)K+vj-1} & \text{if}\;K+vj-1<0\;\text{and}\;1\leq c<i+\frac{K+vj-1}{Ky}\\ 0 & \text{otherwise}, \end{array} \end{array}$$ (77)

where the first equality follows from *t*_0_ = −ln λ and *t*_*f*_ = −ln(λ − *τ*_*f*_). Because *t*_0_ ≤ *t*_*s*_ ≤ *t*_*f*_ and the function 1 − *e*^*t*_0_−*t*^ is increasing in *t*, 0 ≤ *z* ≤ *y* ≤ 1. As a result, we can substitute the new variables (*y*, *v*, *z*) into the objective function Eq (59), the Hamiltonian equation Eq (60) and the terminal condition Eq (61). Moreover, we add the definition of *x*^*i*,*j*,*c*,*c*−1^ and 0 ≤ *z* ≤ *y* ≤ 1. Then we obtain a new optimization problem defined as Eq (86) in section Main results. After solving Eq (86) for (y*, v*, z*), we are able to calculate $u_{t}^{*}$ and $s_{t}^{*}$ (or $u_{\tau }^{*}$ and $s_{\tau }^{*}$ after changing the time index) in order to present theorem 2 and theorem 3.

### Main results

#### Contagion process with no interventions

We first present the theorem when no interventions are provided in the contagion process. For *ϵ* > 0, recall *M*^*ϵ*^ is defined as in definition 5 and note that all indexes (*i*, *j*) are in the range *i* ∨ *j* < *M*^*ϵ*^ in what follows.

**Definition 9**. (*I* function) Define a function *I*: [0, 1] → [0, 1] as

$$\begin{array}{c} I(y)≔\frac{1}{\lambda }\sum_{i\vee j<M^{\epsilon }}j\sum_{c=0}^{i}p\left(i,j,c\right)P\left(\text{Bin}\left(i,y\right)\geq c\right) \end{array}$$ (78)

where Bin(*i*, *y*) denotes a binomial random variable with *i* trials and the probability of occurrence *y*, so P(Bin(i,y)≥c)=∑m=ci(im)ym(1-y)i-m. *I*(*y*) is constructed to represent the asymptotic scaled total out degree from the default set at scaled time *y* and attains its form Eq (78) from the solution of a set of differential equations.

Since $I\left(0\right)=\frac{1}{\lambda }\sum_{i\vee j<M^{\in }}jp\left(i,j,0\right)\geq 0$, and from the definition of λ,

$$\begin{array}{c} I\left(1\right)=\frac{1}{\lambda }\sum_{i\vee j<M^{\epsilon }}j\sum_{c=0}^{i}p\left(i,j,c\right)\leq 1, \end{array}$$ (79)

and *I*(*y*) is continuous and increasing, it has at least one fixed point in [0, 1]. Further define

$$\begin{array}{c} J\left(y\right)≔\sum_{i\vee j<M^{\epsilon }}\sum_{c=0}^{i}p\left(i,j,c\right)P\left(\text{Bin}\left(i,y\right)\geq c\right). \end{array}$$ (80)

**Theorem 1**. (Extends from theorem 3.8 of [24]) Consider a sequence of networks with initial conditions (*P*_*n*_)_*n*≥1_ satisfying assumption 1 where *p* = (*p*(*i*, *j*, *c*))_*i*,*j*,0≤*c*≤*i*_ such that *p*(*i*, *j*, *c*) = 0 for *i* ∨ *j* ≥ *M*^*ϵ*^, 0 ≤ *c* ≤ *i* and no interventions are implemented, let y* ∈ [0, 1] be the smallest fixed point of *I*.

1. If y* = 1, then asymptotically almost all nodes default during the contagion process, i.e.
  $$\begin{array}{c} \frac{D_{T_{n}}}{n}\overset{p}{\to }1. \end{array}$$ (81)
2. If y* < 1 and it is a stable fixed point, i.e. *I*′(y*) < 1, then asymptotically the fraction of final defaulted nodes
  $$\begin{array}{c} \frac{D_{T_{n}}}{n}\overset{p}{\to }J\left(y^{*}\right). \end{array}$$ (82)
  Particularly, if *I*(0) = 0 and *I*′(0) < 1, then
  $$\begin{array}{c} \frac{D_{T_{n}}}{n}\overset{p}{\to }\sum_{i\vee j<M^{\epsilon }}p\left(i,j,0\right). \end{array}$$ (83)

**Remark 8**. Theorem 1 states that the stopping time of the default contagion process is fully governed by the smallest fixed point of the function *I*(*y*) and the asymptotic fraction of defaulted nodes at the end of the process can be 1, 0 or a fractional number, representing almost all nodes default, almost no nodes default or a partial number of nodes default, respectively.

#### Contagion process with interventions

We present the theorems for the contagion process with interventions as the result of solving the finite dimensional optimal control problem (FOCP) in definition 8. For *ϵ* > 0, recall *M*^*ϵ*^ is defined as in definition 5. By lemma 1, the optimal objective function value of (FOCP) is within (*K* + 1)*ϵ* distance to the optimal objective function value of the infinite dimensional Eq (39). That is why we solved (FOCP) and present the results below with (*i*, *j*) in the range *i* ∨ *j* < *M*^*ϵ*^ in the following. From remark 6, for a given vector *p* = (*p*(*i*, *j*, *c*))_0≤*i*,0≤*j*,0≤*c*≤*i*_, the restriction of (*i*, *j*) to *i* ∨ *j* < *M*^*ϵ*^ indicates that we use only *p*(*i*, *j*, *c*), *i* ∨ *j* < *M*^*ϵ*^, 0 ≤ *c* ≤ *i* in the calculation. It is equivalent to setting *p*(*i*, *j*, *c*) = 0, *i* ∨ *j* ≥ *M*^*ϵ*^, 0 ≤ *c* ≤ *i* while keeping *p*(*i*, *j*, *c*), *i* ∨ *j* < *M*^*ϵ*^, 0 ≤ *c* ≤ *i* unchanged, which implies asymptotically nodes with *i* ∨ *j* ≥ *M*^*ϵ*^ are all invulnerable (Note all *p*(*i*, *j*, *c*) need not to sum up to one).

First we define the optimization problem Eq (86) based on which we present theorem 2 and theorem 3.

**Definition 10**. ($\tilde{I}$ and $\tilde{J}$ function) Let *x* = (*x*^*β*^)_*β*∈Φ^*ϵ*^_ where *x*^*β*^ = *x*^*β*^(*y*, *v*) and *p* = (*p*(*i*, *j*, *c*))_*i*,*j*,0≤*c*≤*i*_. We define the functions $\tilde{I}\left(y,v,z\right)$ and $\tilde{J}\left(y,v,z\right)$ as

$$\begin{array}{cc} \tilde{I}\left(y,v,z\right) & =\frac{1}{\lambda }\sum_{i\vee j<M^{\epsilon }}j\\ & [\sum_{c=0}^{i}p\left(i,j,c\right)P\left(\text{Bin}\left(i,x^{i,j,c,c-1}\right)\geq c\right)-1_{\left(vj-1=-K\right)}p\left(i,j,i\right)\left(y^{i}-z^{i}\right)], \end{array}$$ (84)

$$\begin{array}{cc} \tilde{J}\left(y,v,z\right) & =\sum_{i\vee j<M^{\epsilon }}\\ & [\sum_{c=0}^{i}p\left(i,j,c\right)P\left(\text{Bin}\left(i,x^{i,j,c,c-1}\right)\geq c\right)-1_{\left(vj-1=-K\right)}p\left(i,j,i\right)\left(y^{i}-z^{i}\right)]. \end{array}$$ (85)

Note we may write $\tilde{I}\left(y;v,z\right)$ and $\tilde{J}\left(y;v,z\right)$ to indicate that we treat *y* as the variable and *v*, *z* as the fixed parameters. To present the main results, we define the following optimization problem.

**Definition 11**. Define the following optimization problem.

$$\begin{array}{lll} & & \left(86\right)\\ \underset{y,v,z}{\min} & K\cdot \tilde{r}\left(y,v,z\right)+\tilde{J}\left(y,v,z\right) & \left(87\right)\\ \text{st} & \left(1-y\right)\tilde{H}\left(y,v\right)=\lambda v\left(1-y\right) & \left(88\right)\\ & \tilde{I}\left(y,v,z\right)=y & \left(89\right)\\ & x^{i,j,c,c-1}=\{\begin{array}{cc} y & \text{if}\;K+vj-1\geq 0\;\text{or}\;c=0\\ 1-\left(1-y\right)\frac{\left(i-c\right)K}{\left(i-c+1\right)K+vj-1} & \text{if}\;K+vj-1<0\\ & \;\text{and}\;1\leq c<i+\frac{K+vj-1}{Ky}\\ 0 & \text{otherwise} \end{array} & \\ & \forall \left(i,j,c,c-1\right)\in \Phi^{\epsilon } & \left(90\right)\\ & \begin{array}{c} 0\leq z\leq y\leq 1\\ y,v,z\in \mathbb{R}, \end{array} & \left(91\right) \end{array}$$

where

$$\begin{array}{cc} \tilde{r}\left(y,v,z\right) & =\sum_{i\vee j<M^{\epsilon }}\{\sum_{c=1}^{i}p\left(i,j,c\right)\\ & \sum_{m=c}^{i}\sum_{n=0}^{c-1}\left(m-c+1\right)P\left(\text{Multin}\left(i,x^{i,j,c,c-1},y-x^{i,j,c,c-1},1-y\right)=\left(n,m-n,i-m\right)\right)\\ & +1_{\left(vj-1=-K\right)}p\left(i,j,i\right)\left(y^{i}-z^{i}\right)\},\\ \tilde{H}\left(y,v\right) & =\sum_{i\vee j<M^{\epsilon }}\max\left\{-K,vj-1\right\}i\sum_{c=1}^{i}p\left(i,j,c\right)\\ & [P\left(\text{Bin}\left(i-1,y\right)\geq c-1\right)-P\left(\text{Bin}\left(i-1,x^{i,j,c,c-1}\right)\geq c\right)], \end{array}$$ (92)

where Bin(*i*, *y*) denotes a binomial random variable in *i* trials with the probability of occurrence *y*, so P(Bin(i,y)≥c)=∑m=ci(im)ym(1-y)i-m and Multin(*i*, *x*, *y*, 1 − *x* − *y*) = (*a*, *b*, *i* − *a* − *b*) denotes a multinomial distribution in *i* trials with the probabilities of occurrence of each of three types being *x*, *y* and 1 − *x* − *y*, and turns out to have *a*, *b* and *i* − *a* − *b* occurrences of each type, respectively, so P(Multin(i,x,y,1-x-y)=(a,b,i-a-b))=(ia,b,i-a-b)xayb(1-x-y)i-a-b.

**Remark 9**. A feasible solution (*y*, *v*, *z*) has its own meanings for the optimal control problem Eq (39) on the deterministic process (*s*_*τ*_)_*τ*∈[0,λ]_: $y=\frac{\tau_{f}}{\lambda }$ is the scaled end time of the process; *v* is an intervention indicator in that we should intervene on nodes with out degree *j* satisfying *vj* − 1 ≤ −*K* and *v* also determines the starting time of the intervention; *z* specifies the starting time of the intervention for nodes in state (*i*, *j*, *i*, *i* − 1) when *vj* − 1 = −*K*. Moreover, the auxiliary variables (*x*^*i*,*j*,*c*,*c*−1^)_(*i*,*j*,*c*,*c*−1)∈Φ^*ϵ*^_ specifies the starting time of interventions for nodes with different (*i*, *j*) values.

Then we are ready to present the next main theorem about the optimal control policy.

**Theorem 2**. For the asymptotic control problem Eq (9), then

1. Consider a sequence of networks with initial conditions (*P*_*n*_)_*n*≥1_ satisfying assumption 1 where *p* = (*p*(*i*, *j*, *c*))_*i*,*j*,0≤*c*≤*i*_ such that *p*(*i*, *j*, *c*) = 0 for *i* ∨ *j* ≥ *M*^*ϵ*^, 0 ≤ *c* ≤ *i*.
2. Let (*G*_*n*_)_*n*≥1_ be the sequence of control policies for the contagion process on the sequence of networks and (*G*_*n*_)_*n*≥1_ satisfy assumption 2.
3. Let (y*, v*, z*) be the optimal solution for the optimization problem Eq (86).

If y* = 1, or y* ∈ [0, 1) and it is a stable fixed point of $\tilde{I}\left(y;v^{*},z^{*}\right)$, i.e. $\frac{d}{dy}\tilde{I}\left(y^{*};v^{*},z^{*}\right)<1$, the optimal control policy $G_{n}^{*}=\left(g_{1}^{(n)*},\ldots ,g_{m}^{(n)*}\right)$ satisfies that for 0 ≤ *k* ≤ *m* − 1,

$$\begin{array}{c} g_{k+1}^{(n)*}\left(s,w\right)=\{\begin{array}{cc} 1_{\left(k\geq n\lambda {\left(x^{*}\right)}^{i,j,c,c-1}\right)} & \text{if}\;w=\left(i,j,c,c-1\right)\in \Phi^{\epsilon }\;\text{except}\;\\ & c=i\;\text{and}\;v^{*}j-1=-K\\ 1_{\left(k\geq n\lambda z^{*}\right)} & \text{if}\;w=\left(i,j,i,i-1\right)\;\text{and}\;v^{*}j-1=-K\\ 0 & \text{otherwise}, \end{array} \end{array}$$ (93)

where

$$\begin{array}{cc} & {\left(x^{*}\right)}^{i,j,c,c-1}=\{\begin{array}{cc} y^{*} & \text{if}\;K+v^{*}j-1\geq 0\;\text{or}\;c=0\\ 1-\left(1-y^{*}\right)\frac{(i-c)K}{\left(i-c+1\right)K+v^{*}j-1} & \text{if}\;K+v^{*}j-1<0\;\text{and}\;\\ & 1\leq c<i+\frac{K+v^{*}j-1}{Ky^{*}}\\ 0 & \text{otherwise}, \end{array} \end{array}$$ (94)

for (*i*, *j*, *c*, *c* − 1)∈Φ^*ϵ*^.

The next theorem states conclusions for the asymptotic fraction of final defaulted nodes under the optimal policy satisfying theorem 2.

**Theorem 3**. For the asymptotic control problem Eq 9, then

1. Consider a sequence of networks with initial conditions (*P*_*n*_)_*n*≥1_ satisfying assumption 1 where *p* = (*p*(*i*, *j*, *c*))_*i*,*j*,0≤*c*≤*i*_ such that *p*(*i*, *j*, *c*) = 0 for *i* ∨ *j* ≥ *M*^*ϵ*^, 0 ≤ *c* ≤ *i*.
2. Let (*G*_*n*_)_*n*≥1_ be the sequence of control policies for the contagion process on the sequence of networks and (*G*_*n*_)_*n*≥1_ satisfy assumption 2.
3. Let (y*, v*, z*) be the optimal solution for the optimization problem Eq (86).

Then under the optimal control policy $G_{n}^{*}$ as in theorem 2, we have the following conclusions for the asymptotic fraction of final defaulted nodes.

1. If y* = 1, then asymptotically almost all nodes default during the default contagion process, i.e.
  $$\begin{array}{c} \frac{D_{T_{n}}}{n}\overset{p}{\to }1. \end{array}$$ (95)
2. If y* ∈ [0, 1) and it is a stable fixed point of $\tilde{I}\left(y;v^{*},z^{*}\right)$, i.e. $\frac{d}{dy}\tilde{I}\left(y^{*};v^{*},z^{*}\right)<1$, then asymptotically the fraction of final defaulted nodes
  $$\begin{array}{c} \frac{D_{T_{n}}}{n}\overset{p}{\to }\tilde{J}\left(y^{*},v^{*},z^{*}\right), \end{array}$$ (96)
  where *x*^*i*,*j*,*c*,*c*−1^ in $\tilde{J}$ is
  $$\begin{array}{cc} & x^{i,j,c,c-1}=\{\begin{array}{cc} y^{*} & \text{if}\;K+v^{*}j-1\geq 0\;\text{or}\;c=0\\ 1-\left(1-y^{*}\right)\frac{(i-c)K}{\left(i-c+1\right)K+v^{*}j-1} & \text{if}\;K+v^{*}j-1<0\\ & \;\text{and}\;1\leq c<i+\frac{K+v^{*}j-1}{Ky^{*}}\\ 0 & \text{otherwise}, \end{array} \end{array}$$ (97)
  for (*i*, *j*, *c*, *c* − 1) ∈ Φ^*ϵ*^. Particularly, if y* = 0 and $\frac{d}{dy}\tilde{I}\left(0;v^{*},z^{*}\right)<1$, then
  $$\begin{array}{c} \frac{D_{T_{n}}}{n}\overset{p}{\to }\sum_{i\vee j<M^{\epsilon }}p\left(i,j,0\right), \end{array}$$ (98)
  i.e. the final defaulted nodes only consist of the initially defaulted nodes.

In theorem 3 the first case indicates that the network is highly vulnerable and interventions are costly, then the regulator rather lets the whole network default without implementing any interventions, while in the second case interventions are less expensive or the contagion effect is not as high, it is better for the regulator to implement interventions to counteract the contagion process.

#### Discussion and summary

The key to solve Eq (86) depends on solving the two equations Eqs (88) and (89). First we claim that the optimal v* for Eq (86) must be nonpositive.

**Lemma 4**. For Eq (86), the optimal v* ≤ 0.

Here we give an algorithm to solve Eq (86) numerically.

**Algorithm 1**. Solving Eq (86) numerically.

1. Assume *v* = 0,
  - if *K* = 1, then solve Eqs (88) and (89) by e.g. Newton’s method, for *y* and *z*;
  - if *K* ≠ 1, then Eq (86) does not depend on *z*, so solve for *y* and let *z* ≤ *y* arbitrary.
2. Assume *v* < 0,
  - if *K* = 1, then Eq (86) does not depend on *z*, so solve for *y* and *v* such that *v* < 0 and let *z* ≤ *y* arbitrary;
  - if *K* ≠ 1,
  - let *y* = *z* and solve Eqs (88) and (89) for *y* and *v* such that 0 ≤ *y* ≤ 1 and *v* < 0;
  - if additionally *K* > 1, let $v=\frac{1-K}{j}$ for *j* > 0 and solve Eqs (88) and (89) for *y* and *z* such that 0 ≤ *z* ≤ *y* ≤ 1 for each *j* ∈ {1, …, *M*^*ϵ*^}.
3. Choose among all the solutions above (if any) the one that minimizes the objective function Eq (87).

**Fig 5:**
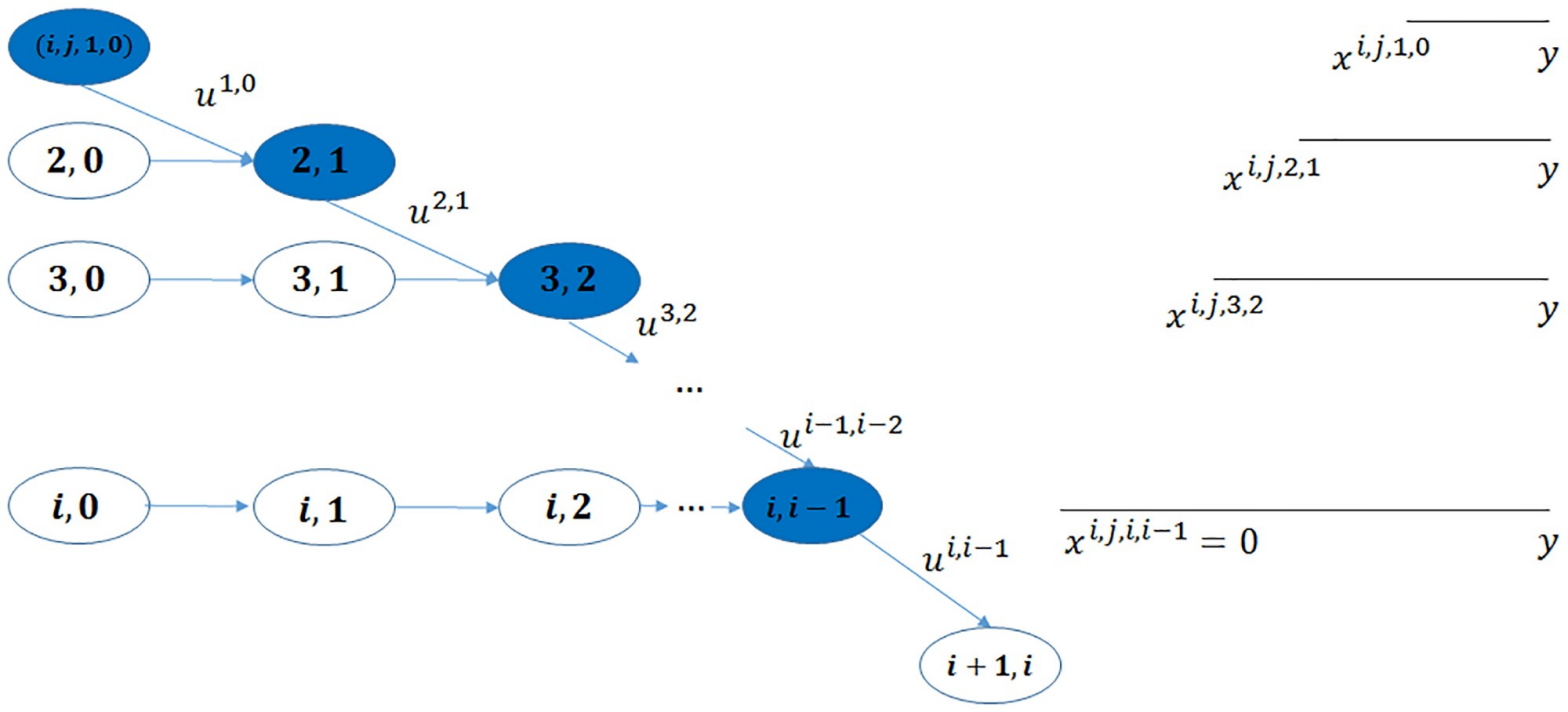
Optimal intervention policy summary where the states indicating one unit of equity remaining are colored in blue.

Recall that a node is in state (*i*, *j*, *c*, *l*) if it has in and out degree pair (*i*, *j*), the sum of initial equity and accumulative interventions *c* (called total buffer) and *l* revealed in-links. Similar to [24], we call the in-links to a node that has only one unit of equity remaining (“distance to default” equal to one) as “contagious” links. So a node in state (*i*, *j*, 1, 0) has *i* contagious links and a node in state (*i*, *j*, 2, 1) has *i* − 1 contagious links and so on, as shown in Fig 5 the states associated with contagious links are colored in blue. The insights from the optimal interventions policy are summarized as follows.

1. It is never optimal to intervene on a node if it is not selected or has at least two units of remaining equity when selected. Thus the optimal control policy described in theorem 2 only specifies the optimal intervention decision on a node that, when selected, has one unit of equity remaining, i.e. *l* = *c* − 1. In other words, the use of interventions is to counteract the effects of contagious links.
2. The optimal control policy depends strongly on *K*, the relative cost of interventions. At a higher *K* value, interventions are costly and the regulator rather lets the contagion to evolve without any interventions. At a lower *K* value, the regulator implements more and more interventions, even a “complete” intervention strategy, that is, intervening on every selected node having the “distance to default” of one since the beginning of the process.
3. The optimal control policy is “monotonic” concerning the number of out degree of a node. There exists a cutoff value of the out degree such that it is only optimal to intervene on a node with out degree larger than this cutoff value and not otherwise, regardless of its in degree, total buffer and revealed in-links. For nodes with out degree equal to the cutoff value, we have the singular control case that only those in state (*i*, *j*, *i*, *i* − 1) needs interventions and the starting time of interventions is determined by the variable *z* from the optimization problem Eq (86).
4. For nodes that are candidates to receive interventions, the starting time of interventions (depends on the variable *x*^*i*,*j*,*c*,*c*−1^) is “monotonic” in terms of the total buffer. The higher the total buffer is, the earlier we should begin to intervene as illustrated in Fig 5 that *x*^*i*,*j*,*c*,*c*−1^ is decreasing in *c*. Moreover, the starting time is also “monotonic” in terms of the in and out degree. For the same out degree, the smaller the in degree is, the earlier the intervention should begin. If *v* < 0, the larger the out degree is, the earlier the intervention should begin. The economic meaning is that we should focus on systematically important nodes as well as nodes that are close to invulnerability and thus easier to save. In other words, we achieve our objective by protecting the nodes that would incur large impact on the network after defaulting and by bringing easy-to-save nodes into invulnerable states.
5. Once we have begun intervening on a node we should keep implementing interventions on it every time it is selected. In other words, we do not allow nodes that have received interventions to default. This is an interesting result. In the partial information setting, [3] consider the interventions on banks that record more write-downs later and default as the “wasted government money” and mention the tradeoff associated with intervention: potentially wasted money versus less capital depletion. But our finding is that there is no wasted money under the optimal policy and the tradeoff is the high intervention cost versus less magnitude of defaults.

Indeed, following the optimal policy, we are able to achieve earlier termination time of the contagion process and smaller fraction of final defaulted nodes. We can quantify the improvement by comparing $\tilde{I}$ and $\tilde{J}$ in theorem 3 with *I* and *J* defined in theorem 1, respectively. Note in the following we suppress the apostrophe “*” and the indexes (*i*, *j*) are in the range *i* ∨ *j* < *M*^*ϵ*^.

1. $\tilde{I}\left(y,v,z\right)$ plays the same role as *I*(*y*) in theorem 1, which represents the asymptotic scaled total out degree from the default set at scaled time *y*. Since
  $$\begin{array}{cc} \; & I\left(y\right)-\tilde{I}\left(y,v,z\right)\\ = & \frac{1}{\lambda }\sum_{i\vee j<M^{\epsilon }}j\{\sum_{c=0}^{i}p\left(i,j,c\right)\left[P\left(\text{Bin}\left(i,y\right)\geq c\right)-P\left(\text{Bin}\left(i,x^{i,j,c,c-1}\right)\geq c\right)\right]\\ & +1_{\left(vj-1=-K\right)}p\left(i,j,i\right)\left(y^{i}-z^{i}\right)\}, \end{array}$$ (99)
  and note that
  $$\begin{array}{c} x^{i,j,c,c-1}\leq y\;\text{for}\;\left(i,j,c,c-1\right)\in \Phi^{\epsilon }, \end{array}$$ (100)
  thus
  $$\begin{array}{c} P\left(\text{Bin}\left(i,y\right)\geq c\right)-P\left(\text{Bin}\left(i,x^{i,j,c,c-1}\right)\geq c\right)\geq 0. \end{array}$$ (101)
  Then for the same initial conditions *p* = (*p*(*i*, *j*, *c*))_*i*∨*j*<*M*^*ϵ*^,0≤*c*≤*i*_, the smallest fixed point of $\tilde{I}\left(y;v^{*},z^{*}\right)$ is no greater than that of *I*(*y*), which implies that the default contagion process under optimal interventions terminates no later than under no interventions.
2. Similarly $\tilde{J}\left(y,v,z\right)$ plays the same role as *J*(*y*) in theorem 1, which represents the asymptotic fraction of final defaulted nodes under the optimal control policy. The difference
  $$\begin{array}{cc} \; & J\left(y\right)-\tilde{J}\left(y,v,z\right)\\ = & \sum_{i\vee j<M^{\epsilon }}\{\sum_{c=0}^{i}p\left(i,j,c\right)\left[P\left(\text{Bin}\left(i,y\right)\geq c\right)-P\left(\text{Bin}\left(i,x^{i,j,c,c-1}\right)\geq c\right)\right]\\ & +1_{\left(vj-1=-K\right)}p\left(i,j,i\right)\left(y^{i}-z^{i}\right)\}\geq 0 \end{array}$$ (102)
  quantifies the fraction of nodes that are prevented from default because of the optimal control policy.

## Numerical experiments

### Introduction

While the theoretical framework described before allows heterogeneous networks with degree sequences and initial equity levels drawn from arbitrary distributions, we present a relatively simplified case in numerical experiments for illustration purpose, in which the degree sequences and initial equity levels satisfy specific distributions. Consider a sequence of networks with the number of nodes *n* growing to infinity, whose in and out degrees are between 1 and 10, and each node’s in degree equal to its own out degree, i.e. *d*^−^(*v*) = *d*^+^(*v*), *v* ∈ [*n*], respectively, so we call either the in or out degree as the degree of the node. This allows us to combine two indexes *i* and *j* into one index *i*, so the state of a node becomes (*i*, *c*, *l*) and the empirical probability *P*_*n*_ and the limiting probability *p* of the degree and initial equity become *P*_*n*_(*i*, *c*) and *p*(*i*, *c*) respectively. Additionally we assume the initial equity levels between 1 and 10. In sum, we consider the degree and initial equity level pair in the set $\Gamma^{{}^{\prime }}≔\{\left(i,c\right)\in N_{0}^{2}:1\leq i\leq 10,0\leq c\leq 10\}$.

Next we decide on the limiting probability *p*. Note that Γ′ contains three initial types of nodes: defaulted (with *c* = 0), vulnerable (with *c* ≤ *i*) and invulnerable (with *c* > *i*). In this numerical experiment, we set the total fraction of initial defaults as *ξ* and assume the fraction of initial defaults is the same across all degrees, i.e. $p\left(i,0\right)=\frac{\xi }{10}$ for *i* ∈ [1, 10]. For the initially liquid nodes, the joint probability of the degree and initial equity conditional on being liquid is constructed through a binormal copula with correlation *ρ* and two marginal probabilities. The marginal probabilities of the degree and initial equity are assumed to follow the Zipf’s law, i.e.

$$\begin{array}{cc} P(\text{deg}=i)= & \frac{i^{-(1+a_{1})}}{\sum_{l=1}^{10}l^{-(1+a_{1})}},\\ P(\text{initial}\;\text{equity}=c)= & \frac{c^{-(1+a_{2})}}{\sum_{l=1}^{10}l^{-(1+a_{2})}}, \end{array}$$ (103)

where *a*_1_, *a*_2_ > 0. The Zipf’s law is a form of the power law with Pareto tails, which is observed for the distribution of the degrees and equity levels of the financial networks in many empirical studies, see e.g. [19, 29].

In a network of size *n* with the joint probability *P*_*n*_(*i*, *c*) of the degree and initial equity, a contagion process under interventions occurs as described in section Dynamics. Recall that we only need to consider intervening on a node that, when selected, has only one unit of equity left, i.e. a node with “distance to default” equal to one. Here we consider two types of intervention policies, the optimal policy and the alternative policy: intervening on nodes with degree between 8 and 10 and “distance to default” equal to one from the beginning of the process. The alternative policy implies interventions on nodes with high degrees and close to default, representing the usual policy employed by the central bank or government in a real financial crisis.

Our objective is to verify the convergence in probability of $\frac{R_{T_{n}}}{n}$ and $\frac{D_{T_{n}}}{n}$ as well as the convergence of the scaled termination time $\frac{T_{n}}{m}$ as stated in proposition 5. Moreover, we shall study the convergence rate of the standard deviation and IQR (interquartile range) to examine if the asymptotic variables provide good approximations under realistic *n* values.

Under the optimal policy in the form given in theorem 2, the limits for $\frac{R_{T_{n}}}{n}$, $\frac{D_{T_{n}}}{n}$ and $\frac{T_{n}}{m}$ as *n* → ∞ are $\tilde{r}\left(y^{*},v^{*},z^{*}\right)$, $\tilde{J}\left(y^{*},v^{*},z^{*}\right)$ and y*, respectively in Eq (86) where (y*, v*, z*) is the optimal solution. On the other hand, the alternative policy is that for 0 ≤ *k* ≤ *m* − 1,

$$\begin{array}{c} g_{k+1}^{(n),\text{alt}}\left(s,w\right)=\{\begin{array}{cc} 1_{\left(k\geq n\lambda x_{\text{alt}}^{i,c,c-1}\right)} & \text{if}\;w=\left(i,c,c-1\right)\in \Phi^{\prime }\\ 0 & \text{otherwise}, \end{array} \end{array}$$ (104)

where for (*i*, *c*, *c* − 1)∈Φ′,

$$\begin{array}{c} x_{\text{alt}}^{i,c,c-1}=\{\begin{array}{cc} 0 & \;\text{if}\;i\in \{8,9,10\}\\ y & \text{otherwise}, \end{array} \end{array}$$ (105)

and *y* is the solution of $\frac{1}{\lambda }\sum_{i=0}^{10}i\sum_{c=0}^{i}p\left(i,c\right)P\left(\text{Bin}\left(i,x_{\text{alt}}^{i,c,c-1}\right)\geq c\right)=y$. Then the limits for $\frac{R_{T_{n}}}{n}$, $\frac{D_{T_{n}}}{n}$ and $\frac{T_{n}}{m}$ as *n* → ∞ can be calculated as:

$$\begin{array}{cc} \frac{R_{T_{n}}}{n}\overset{p}{\to } & \sum_{i=0}^{10}\sum_{c=1}^{i}p\left(i,c\right)\\ & \sum_{m=c}^{i}\sum_{n=0}^{c-1}\left(m-c+1\right)P(\text{Multin}\left(i,x_{\text{alt}}^{i,c,c-1},y-x_{\text{alt}}^{i,c,c-1},1-y\right)\\ & =(n,m-n,i-m)),\\ \frac{D_{T_{n}}}{n}\overset{p}{\to } & \sum_{i=0}^{10}\sum_{c=0}^{i}p\left(i,c\right)P\left(\text{Bin}\left(i,x_{\text{alt}}^{i,c,c-1}\right)\geq c\right),\\ \frac{T_{n}}{m}\overset{p}{\to } & y, \end{array}$$ (106)

where P(Bin(i,y)≥c)=∑m=ci(im)ym(1-y)i-m and P(Multin(i,x,y,1-x-y)=(a,b,i-a-b))=(ia,b,i-a-b)xayb(1-x-y)i-a-b.

### Simulation

#### The set up

We have the following setup.

1. A sequence of six networks with increasing number of nodes *n* ∈ {5^4^, 6^4^, …, 10^4^} and there are 100 runs for each network under either intervention policy.
2. To determine the asymptotic fraction *p*(⋅, ⋅) of the degree and initial equity pair (*i*, *c*) where (*i, c*) ∈ Γ′, we set the following parameters.
  - The fraction of initial defaults *ξ* = 0.5, indicating half of the nodes have defaulted. As stated before, we assume in this numerical experiment that the fraction of initial defaults is the same across all degrees, thus $p\left(i,0\right)=\frac{\xi }{10}$ for *i* ∈ [1, 10].
  - The probability of the degree and initial equity for liquid nodes *p*(*i*, *e*), *i* ∈ {1, …, 10}, *e* ∈ {1, …, 10} is determined by a binormal coupula with the exponents of the marginal probabilities of the degree and initial equity (*a*_1_, *a*_2_) = (0.8, 0.7) and the correlation coefficient *ρ* = 0.9. Note that a smaller *a*_1_ indicates larger fraction of nodes with higher degrees, thus higher connectivity and a smaller *a*_2_ indicates larger fraction of nodes with higher initial equities, and *ρ* implies how likely that higher degree nodes have higher initial equities.
3. After determining the asymptotic fraction *p*(⋅, ⋅), we construct a sequence of empirical fractions *P*_*n*_(⋅, ⋅) for each network that converge to *p*(⋅, ⋅) by
  $$\begin{array}{c} P_{n}\left(i,c\right)=\frac{\left[np(i,c)\right]}{n}\;\left(i,c\right)\in \Gamma^{{}^{\prime }}, \end{array}$$ (107)
  where [⋅] is the round function. In other words, the number of nodes with degree *i* and initial equity *c* are [*np*(*i*, *c*)] for a network of *n* nodes.
4. We consider two intervention policies described as before.
5. The relative cost for the interventions *K* = 0.5.

In the following we suppress *T*_*n*_ in the subscripts and write $\frac{R}{n}$, $\frac{D}{n}$ and $\frac{T}{m}$ in stead of $\frac{R_{T_{n}}}{n}$, $\frac{D_{T_{n}}}{n}$ and $\frac{T_{n}}{m}$ respectively.

#### Theoretical results

The theoretical limits of $\frac{R}{n}$, $\frac{D}{n}$ and $\frac{T}{m}$ under the optimal and alternative policies are summarized in Table 1.

**Table 1:** Theoretical limits of $\frac{R}{n}$, $\frac{D}{n}$ and $\frac{T}{m}$ under the optimal and alternative policies.

Policies	$\frac{R}{n}$	$\frac{D}{n}$	$K\frac{R}{n}+\frac{D}{n}$	$\frac{T}{m}$
Optimal	0.306	0.503	0.657	0.728
Alternative	0.019	0.821	0.830	0.866

We first verify that the objective function $K\frac{R}{n}+\frac{D}{n}$ in Eq (86) is indeed less under the optimal policy. Moreover, compared with the alternative policy, the optimal policy intervens more but results in smaller fraction of final defaulted nodes and ends the contagion process earlier.

#### Simulation results

**Fig 6:**
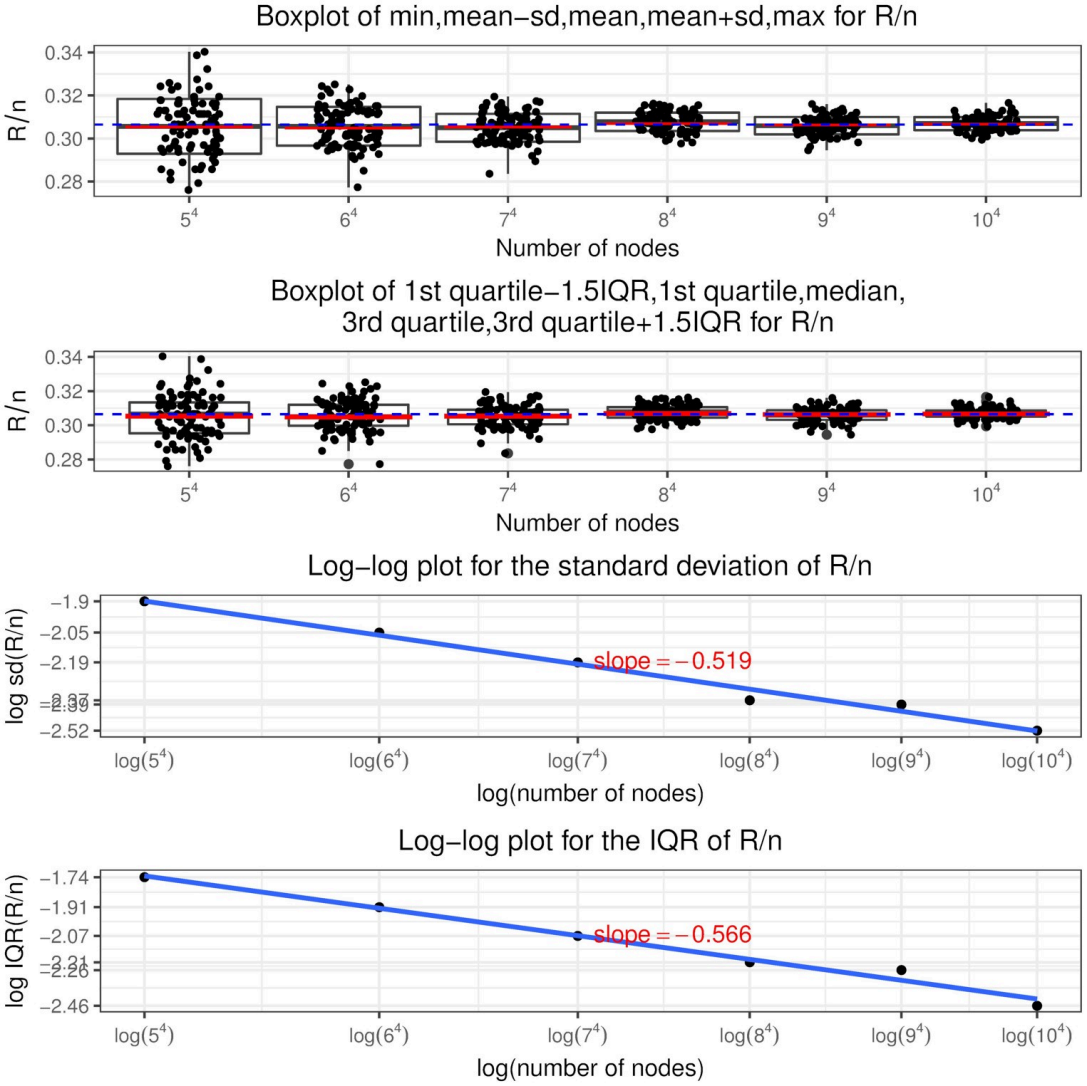
The boxplot and log-log plot of standard deviation and IQR for *R*/*n* under optimal policy.

**Fig 7:**
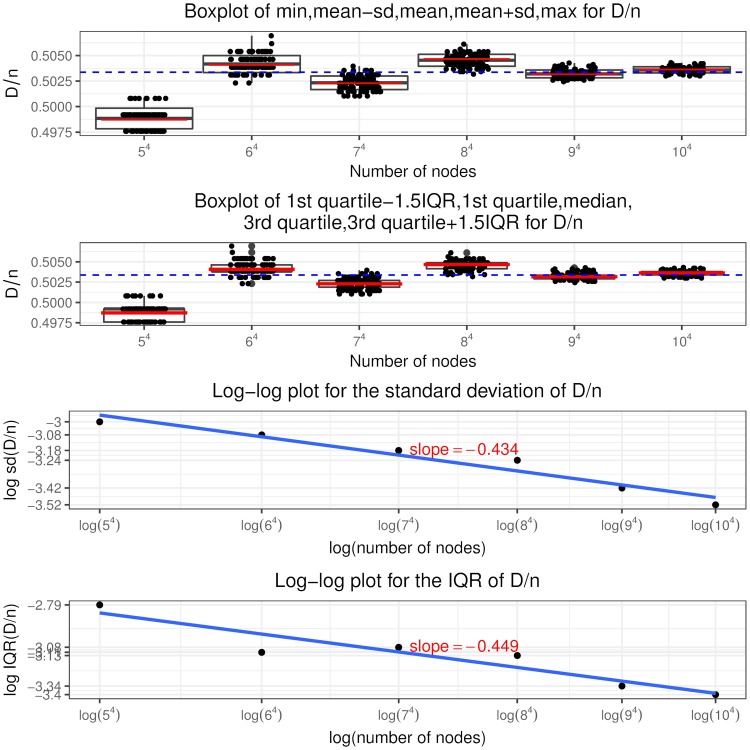
The boxplot and log-log plot of standard deviation and IQR for *D*/*n* under optimal policy.

**Fig 8:**
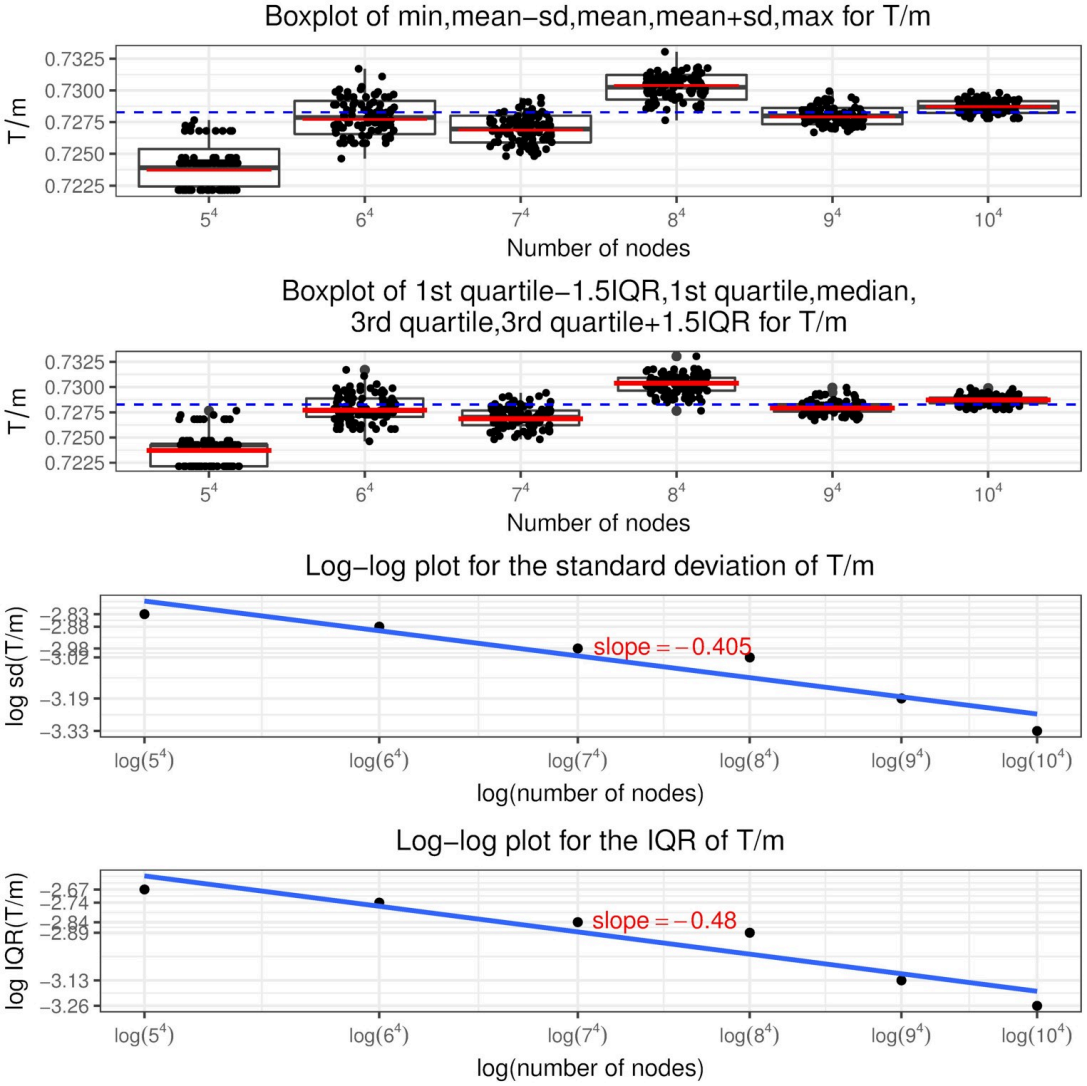
The boxplot and log-log plot of standard deviation and IQR for *T*/*m* under optimal policy.

**Fig 9:**
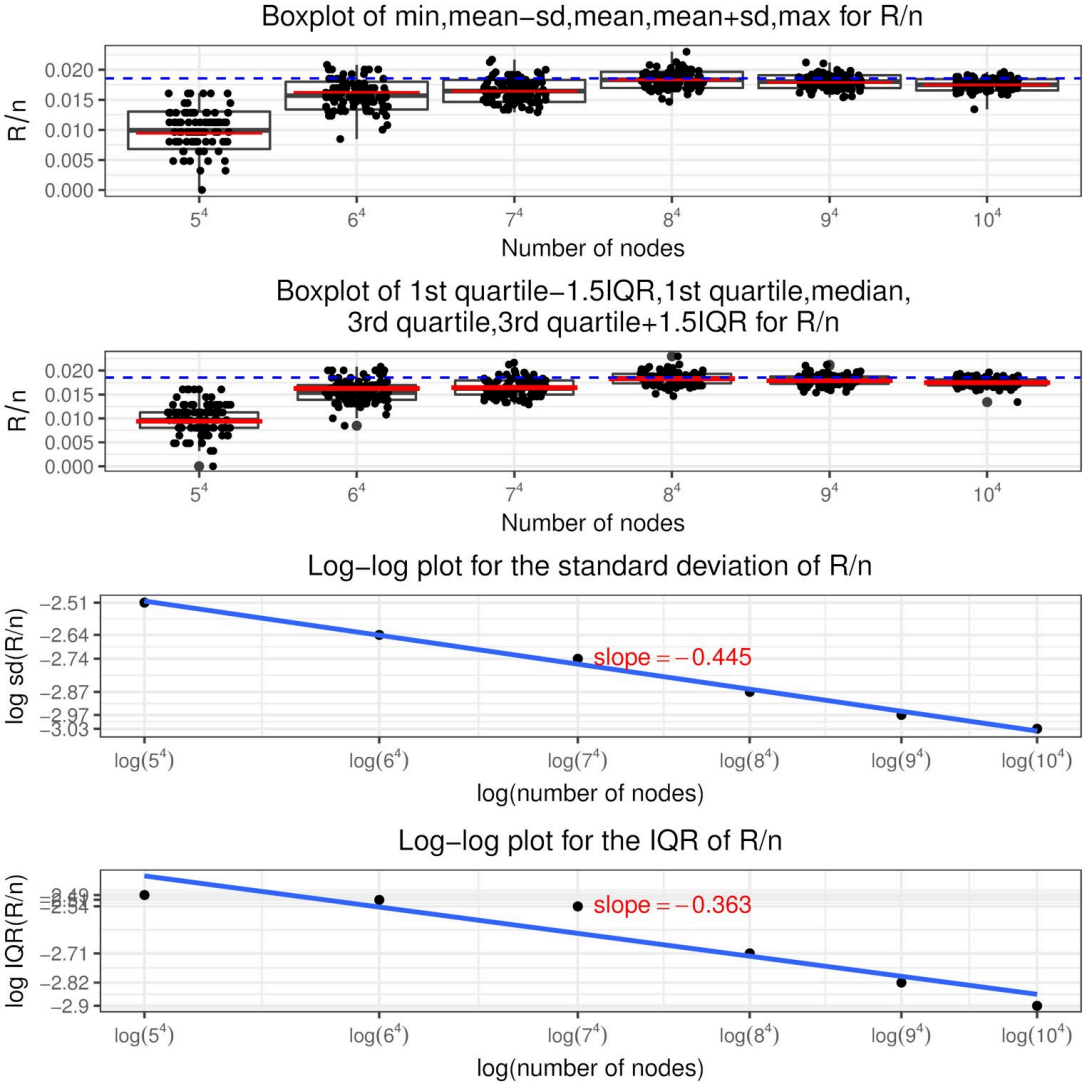
The boxplot and log-log plot of standard deviation and IQR for *R*/*n* under alternative policy.

**Fig 10:**
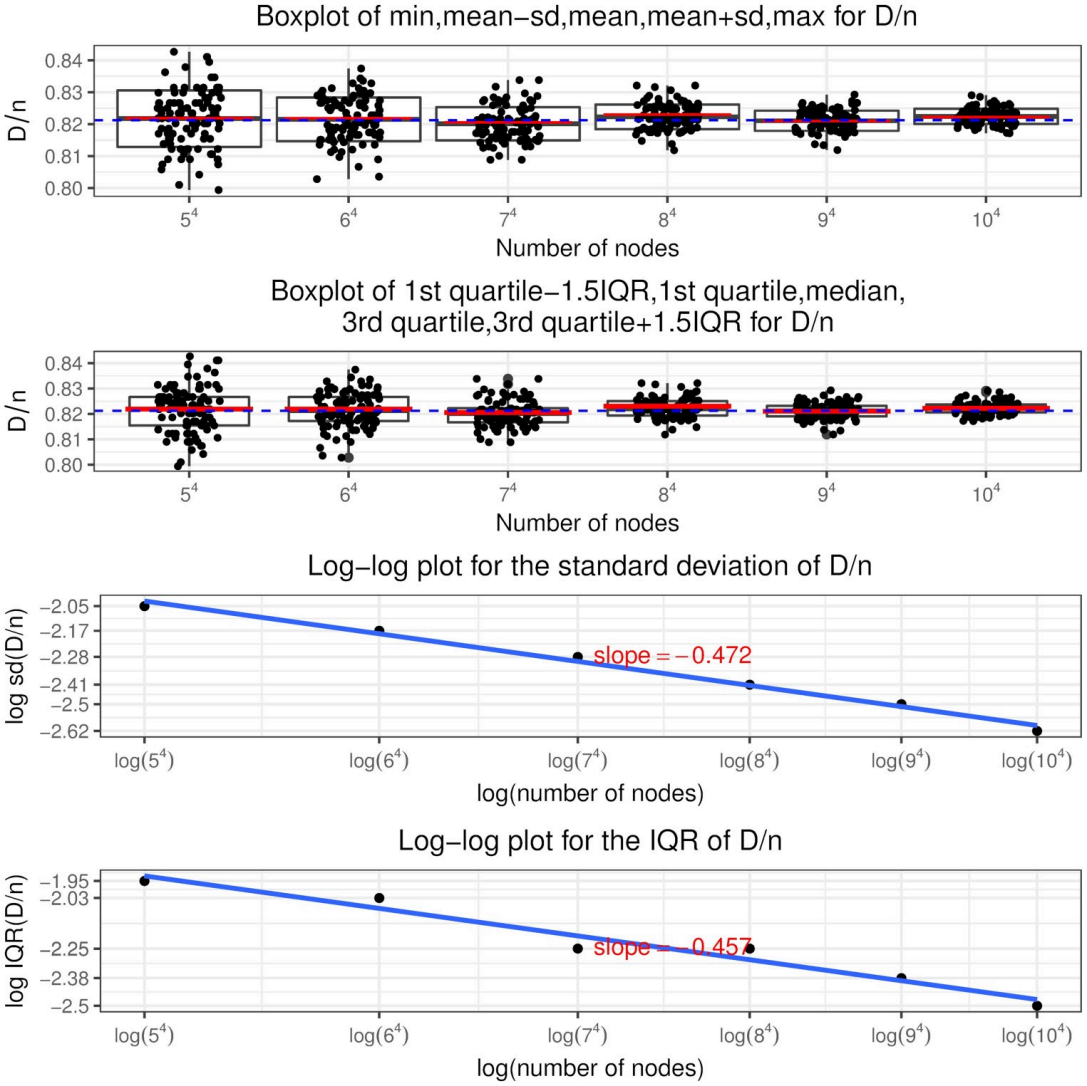
The boxplot and log-log plot of standard deviation and IQR for *D*/*n* under alternative policy.

**Fig 11:**
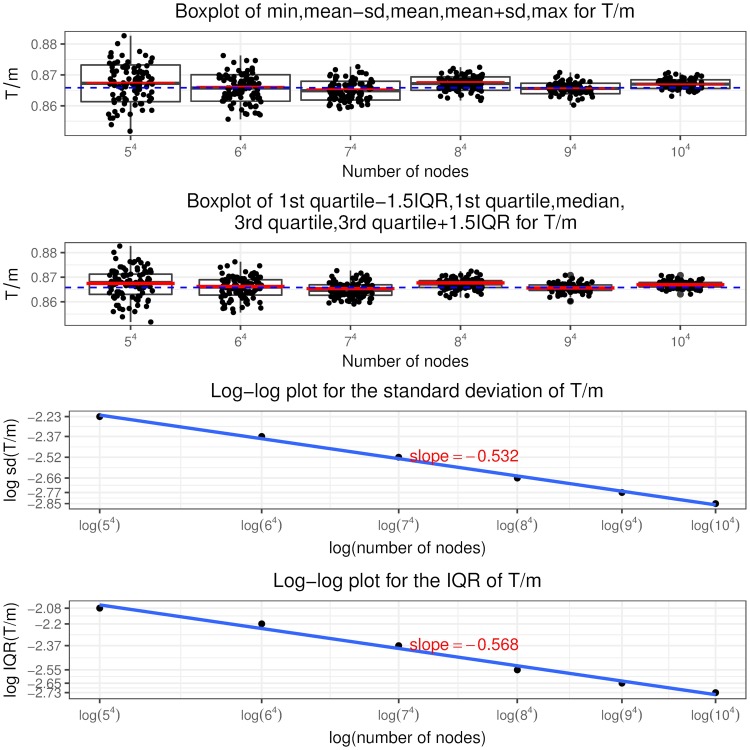
The boxplot and log-log plot of standard deviation and IQR for *T*/*m* under alternative policy.

We show the plots for $\frac{R}{n}$, $\frac{D}{n}$ and $\frac{T}{m}$ under the optimal and alternative policies as shown in Figs 6–11.

1. Under either policy and for each variable, there are four plots in each figure. The first two plots are two boxplots. The above boxplot visualizes five summary statistics (min, mean−standard deviation, mean, mean+ standard deviation, max) while the bottom boxplot uses another set of summary statistics (1st quartile−1.5IQR, 1st quartile, median, 3rd quartile, 3rd quartile+ 1.5IQR) and the data outside the range are treated as outliers, where IQR stands for interquartile range, i.e. the difference between the third and the first quartiles.
2. The blue dashed horizontal line in each plot indicates the theoretical limits of $\frac{R}{n}$, $\frac{D}{n}$ and $\frac{T}{m}$ based on *p*(⋅, ⋅) and the red solid line in each box indicates the theoretical limits of those values calculated with *P*_*n*_(⋅, ⋅) for each *n*. We calculate the theoretical values in both ways because for small *n*, *P*_*n*_(⋅, ⋅) determined by Eq (107) has a relatively large rounding error and thus deviates a bit from *p*(⋅, ⋅) but calculating using *P*_*n*_(⋅, ⋅) instead of *p*(⋅, ⋅) can effectively remove the deviations in the inputs to the model. However, given *p*(⋅, ⋅), *P*_*n*_(⋅, ⋅) is different for different *n* values, thus the theoretical values of a variable calculated with *P*_*n*_(⋅, ⋅) are also different for different *n*’s.
3. The black dots in the boxplots indicates the results of 100 runs and they are jittered by a random amount left and right to avoid overplotting. From the black dots we can see the distributions of the results. Note that the black dots in the above and bottom boxplots show the same results for each *n*. They look different because they are jittered left and right by a different random amount.
4. The last two plots in every figure shows the log-log plot of the standard deviation and IQR of $\frac{R}{n}$, $\frac{D}{n}$ and $\frac{T}{m}$ against *n* and a fitted straight line with the slope.

From the simulation results, we make the following conclusions.

1. From the boxplots of $\frac{R}{n}$, $\frac{D}{n}$ and $\frac{T}{m}$ under both intervention policies, we observe that the mean or median converge to the calculated theoretical value with shrinking standard deviation or IQR. Because the theoretical value is a constant given the joint probability of degree and initial equity *p*(⋅, ⋅), convergence of mean to the theoretical value with variance converging to zero is equivalent to convergence in probability, this observation provides evidence for the convergences in probability of $\frac{R}{n}$, $\frac{D}{n}$ and $\frac{T}{m}$ to their theoretical values, thus proving proposition 5 and theorem 3.
2. Be comparing the blue dashed line and the red solid line we see that the mean or median is closer to the red solid line, i.e. the theoretical value calculated with *P*_*n*_(⋅, ⋅) instead of *p*(⋅, ⋅). This reflects the rounding error caused by Eq (107) in the inputs into the calculation. By using the more accurate fraction we observe that the closeness of the mean or median to the theoretical value does not vary in *n* although the results of different runs are more and more concentrated around the mean or median as *n* grows.
3. The log-log plots of the standard deviation and IQR of each variable with the fitted straight lines further show that both of them decrease with power law tails, i.e. in the form of *z* = *Cx*^−*a*^ where *C* is a constant and *a* > 0 is the exponent. The absolute value of the slope of the straight line serves as the exponent. It is interesting to observe that the exponents for the standard deviation and IQR of different variables are in the range 0.4 ∼ 0.5 under both intervention policies. This implies that the dispersions of all variables converge to zero at roughly the same rate under both policies.

#### Conclusion

From the simulation part we can make the following conclusions.

1. The convergences of $\frac{R}{n}$, $\frac{D}{n}$ and $\frac{T}{m}$ to their theoretical values (stated in proposition 5) are supported by the simulation results. It is worth noting that the closeness of the mean or median to the theoretical value does not vary for different *n* after the rounding error in the initial fractions are removed, but the dispersion of the variable shrinks as *n* grows.
2. The dispersion of each variable decreases following a power law. The exponents are close to each other under both intervention policies and for all variables, indicating a uniform convergence rate of the dispersions of all the variables under both policies.

## Appendix A: Proofs

### Proof of proposition 1

We give a proof in words similar to the proof of proposition 3.4 in [2] for a different objective function of optimizing the value of the financial system at the end of the process under some budget constraint. We observe that the objective function *J*_*n*_ depends on the set of defaulted nodes only through its cardinality. Any node will affect the states of other nodes only after it defaults because the set of unrevealed out links of the default set determining the contagion process grows only after a node defaults. And it is possible for a default to occur only when a node has one unit of equity (distance to default equal to one) at the time of being selected. Before that time, the equity only decreases by one every time it is selected. Moreover, there is always a chance to intervene on a node before it defaults. However, if we intervene on a node that is not selected at the current step or has more than one units of remaining equity when selected, it is possible that the node may not be selected in the following steps before the process ends in which case we implemented redundant interventions without reducing the number of defaults.

### Proof of proposition 2

*Proof*. Assume *u*_*τ*_ = *b* ≔ (*b*^*β*^)_*β*∈Φ_ a constant vector for *τ* ∈ [*τ*_1_, *τ*_2_) ⊆ [0, λ), 0 ≤ *τ*_1_ < *τ*_2_ < ∞ and *b*^*β*^ ∈ {0, 1}. Note that the ODEs are “separable” in that $s_{\tau }^{i,j,c,l}$ only depends on the entries of *s*_*τ*_ with the same (*i*, *j*), so we can only focus on the system of ODEs with the same (*i*, *j*). For the same (*i*, *j*), define Γ_*i*,*j*_ ≔ {(*c*, *l*):0 ≤ *l* < *c* ≤ *i* or *c* = *i* + 1, *l* = *i*} and suppress (*i*, *j*) in the superscripts in definition 4, then we obtain the system of ODEs for *τ* ∈ [*τ*_1_, *τ*_2_) with the initial condition $s_{\tau_{1}}=s_{1}≔{\left(s_{1}^{c,l}\right)}_{\left(c,l\right)\in \Gamma_{i,j}}$.

Letting *t* = −ln(λ − *τ*), *t*_1_ = −ln(λ − *τ*_1_) and *t*_2_ = −ln(λ − *τ*_2_), we have an autonomous system of ODEs for *t* ∈ [*t*_1_, *t*_2_) that

$$\begin{array}{cc} \frac{ds_{t}^{c,0}}{dt}= & -is_{t}^{c,0}\;\text{for}\;1\leq c\leq i,\\ \frac{ds_{t}^{c,l}}{dt}= & \left(i-l+1\right)s_{t}^{c,l-1}-\left(i-l\right)s_{t}^{c,l}\\ & \;\text{for}\;3\leq c\leq i,\;1\leq l\leq c-2,\\ \frac{ds_{t}^{c,c-1}}{dt}= & \left(i-c+2\right)s_{t}^{c-1,c-2}b^{c-1,c-2}\\ & +\left(i-c+2\right)s_{t}^{c,c-2}-\left(i-c+1\right)s_{t}^{c,c-1}\\ & \;\text{for}\;2\leq c\leq i,\\ \frac{ds_{t}^{i+1,i}}{dt}= & s_{t}^{i,i-1}b^{i,i-1}, \end{array}$$ (108)

with the initial condition $s_{t_{1}}=s_{1}$.

By induction, we can prove the solution *s*_*t*_ on [*t*_1_, *t*_2_) is

stc,l=e(i-l)(t1-t)∑r=0ls1c,r(i-rl-r)(1-et1-t)l-rfor2≤c≤i,0≤l≤c-2,stc,c-1=e(i-c+1)(t1-t)∑r=0c-1∑q=r+1c∏k=qc-1bk,k-1s1q,r(i-rc-1-r)(1-et1-t)c-1-rfor1≤c≤i,sti+1,i=s1i+1,i+∑r=0i-1∑q=r+1i∏k=qibk,k-1s1q,r(1-et1-t)i-r, (109)

where $\prod_{k=c}^{c-1}b^{k,k-1}≔1$.

By changing the variable *t* to *τ* by *t* = −ln(λ − *τ*), Eqs (15), (16) and (17) follow. Let the initial condition be $s_{\tau_{1}}^{i,j,c,l}=p\left(i,j,c\right)1_{\left(l=0\right)}$ for (*i*, *j*, *c*, *l*) ∈ Γ at *τ*_1_ = 0, then Eq (18) follows from Eq (15).

### Proof of proposition 3

*Proof*. For the following proof we need to adapt the Wormald’s theorem in Appendix B: Wormald’s theorem. For notational convenience we suppress the tilde sign for $\tilde{R}$, $\tilde{r}$. Since $\frac{m}{n}\to \lambda$ as *n* → ∞, for the given *ϵ* and $\hat{\lambda }=\lambda -\in$, we can find $n_{0}\in N$, such that $0<\hat{\lambda }<\frac{m}{n}<\lambda +0.1$ for *n* ≥ *n*_0_. Let *z* = (*z*^*α*^)_*α*∈Γ^*ϵ*^_ and

$$\begin{array}{c} U=\{\left(\tau ,z,r\right)\in R^{|\Gamma^{\epsilon }|+2}:\;-\epsilon <\tau <\hat{\lambda },\;-\epsilon <z^{\alpha }<1.1\;,-\epsilon <r<\lambda +0.1\}, \end{array}$$ (110)

then *U* contains the closure of

$$\begin{array}{c} \{\left(0,z,0\right):\;P\left(S_{0}^{\alpha }=z^{\alpha }n,\;\forall \alpha \in \Gamma^{\epsilon },R_{0}=0\right)\neq 0\;\text{for}\;\text{some}\;n\}. \end{array}$$ (111)

Define the stopping time $T_{U}=\min\left\{1\leq k\leq m:\left(\frac{k}{n},\frac{S_{k}}{n},\frac{R_{k}}{n}\right)\notin U\right\}$.

By definition 1 and definition of *R*_*k*_, $0\leq S_{k}^{\alpha }\leq n$, *α* ∈ Γ^*ϵ*^ and 0 ≤ *R*_*k*_ ≤ (λ + 0.1)*n* hold ∀*k* ≥ 0 and *n* ≥ *n*_0_. Recall that $S_{k}={\left(S_{k}^{\alpha }\right)}_{\alpha \in \Gamma^{\in }}$ and $\frac{S_{k}}{n}={\left(\frac{S_{k}^{\alpha }}{n}\right)}_{\alpha \in \Gamma^{\in }}$. The following conditions hold:

1. For 0 ≤ *k* < *T*_*U*_ and *α* ∈ Γ^*ϵ*^,
  $$\begin{array}{cc} \left|S_{k+1}^{\alpha }-S_{k}^{\alpha }\right| & \leq 1,\\ |R_{k+1}-R_{k}| & \leq 1, \end{array}$$ (112)
  i.e. *ρ*_1_ = 1.
2. There exists *ρ*_2_ = *O*(*n*^−1^) such that for 0 ≤ *k* < *T*_*U*_ and *α* ∈ Γ^*ϵ*^,
  $$\begin{array}{cc} \left|E(S_{k+1}^{\alpha }-S_{k}^{\alpha }|F_{k})-h^{\alpha }(\frac{k}{n},\frac{S_{k}}{n})\right| & \leq \rho_{2},\\ \left|E(R_{k+1}-R_{k}|F_{k})-h_{0}(\frac{k}{n},\frac{S_{k}}{n})\right| & \leq \rho_{2}, \end{array}$$ (113)
  where *h* = (*h*^*α*^)_*α*∈Γ^*ϵ*^_ and *h*_0_ are
  $$\begin{array}{cc} h^{i,j,c,l}(t,z) & =\{\begin{array}{cc} -\frac{iz^{i,j,c,0}}{\lambda -t} & \text{if}\;1\leq c\leq i,l=0\\ \frac{\left(i-l+1\right)z^{i,j,c,l-1}}{\lambda -t}-\frac{\left(i-l\right)z^{i,j,c,l}}{\lambda -t} & \text{if}\;3\leq c\leq i,1\leq l\leq c-2\\ \frac{\left(i-c+2\right)z^{i,j,c-1,c-2}}{\lambda -t}u_{t}^{i,j,c-1,c-2}\\ +\frac{\left(i-c+2\right)z^{i,j,c,c-2}}{\lambda -t}-\frac{\left(i-c+1\right)z^{i,j,c,c-1}}{\lambda -t} & \text{if}\;2\leq c\leq i\\ \frac{z^{i,j,i,i-1}}{\lambda -t}u_{t}^{i,j,i,i-1} & \text{if}\;c=i,l=i-1, \end{array}\\ h_{0}\left(t,z\right) & =\sum_{\left(i,j,c,c-1\right)\in \Phi^{\epsilon }}\frac{\left(i-c+1\right)z^{i,j,c,c-1}}{\lambda -t}u_{t}^{i,j,c,c-1}. \end{array}$$ (114)
  In particular, Eq (113) follows from
  $$\begin{array}{cc} \; & |\sum_{\left(i,j,c,c-1\right)\in \Phi^{\epsilon }}\frac{\left(i-c+1\right)S_{k}^{i,j,c,c-1}}{m-k}u_{\frac{k}{n}}^{i,j,c,c-1}\\ & -\sum_{\left(i,j,c,c-1\right)\in \Phi^{\epsilon }}\frac{\left(i-c+1\right)\frac{S_{k}^{i,j,c,c-1}}{n}}{\lambda -\frac{k}{n}}u_{\frac{k}{n}}^{i,j,c,c-1}|\\ \leq & \sum_{\left(i,j,c,c-1\right)\in \Phi^{\epsilon }}|\frac{\left(i-c+1\right)\frac{S_{k}^{i,j,c,c-1}}{n}}{\frac{m}{n}-\frac{k}{n}}u_{\frac{k}{n}}^{i,j,c,c-1}-\frac{\left(i-c+1\right)\frac{S_{k}^{i,j,c,c-1}}{n}}{\lambda -\frac{k}{n}}u_{\frac{k}{n}}^{i,j,c,c-1}|\\ = & O(n^{-1}). \end{array}$$ (115)

However, for *β* ∈ Φ^*ϵ*^, *h*^*β*^ and *h*_0_ are not Lipschitz continuous because $u_{\tau }^{\beta }$ can have step changes on [0, λ). So we need to adapt the proof. Note that $u_{\tau }^{\beta }$ is piecewise constant {0, 1} valued function thus *h*^*β*^(*τ*, *s*) and *h*_0_(*τ*, *s*) are Lipschitz continuous in each interval where $\tilde{u}={\left(u^{\beta }\right)}_{\beta \in \Phi^{\in }}$ is a constant vector and then we can apply the Wormald’s theorem in Appendix B: Wormald’s theorem. In the following define *s*_*τ*_(*τ*′, *x*) as the solution of the ODEs,

$$\begin{array}{c} \frac{d}{d\tau }s_{\tau }=h\left(\tau ,s_{\tau }\right), \end{array}$$ (116)

with initial condition at *τ*′, *s*_*τ*′_ = *x* ≔ (*x*^*α*^)_*α*∈Γ^*ϵ*^_.

In what follows define the points where any component of ${\tilde{u}}_{\tau }$ has a step change. $\tau_{l}≔\inf\left\{\tau >\tau_{l-1}:u_{\tau }^{\beta }\;\text{has}\;\text{a}\;\text{step}\;\text{change}\;\text{for}\;\text{some}\;\beta \in \Phi^{\in }\right\}\wedge \hat{\lambda }$ for *l* ≥ 1 and *τ*_0_ ≔ 0. Also let *k*_*l*_ = ⌈*nτ*_*l*_⌉, where ⌈⋅⌉ is the ceiling function. As a result, *k*_*l*_ − 1 < *nτ*_*l*_ ≤ *k*_*l*_. Recall the initial condition $s_{0}={\left(s_{0}^{\alpha }\right)}_{\alpha \in \Gamma^{\in }}$ with $s_{0}^{i,j,c,l}=p\left(i,j,c\right)1_{\left(l=0\right)}$. Because every *u*^*β*^ for *β* ∈ Φ^*ϵ*^ has only a finite number of step changes on [0, λ) and Φ^*ϵ*^ is a finite set, there are in total a finite number of step changes for all the component functions of $\tilde{u}$ on [0, λ).

Then by the Wormald’s theorem, let $\rho =n^{-\frac{1}{4}}$, it follows that

$$\begin{array}{c} \underset{0\leq k\leq k_{1}-1}{\sup}\frac{S_{k}^{\alpha }}{n}-s_{\frac{k}{n}}^{\alpha }\left(0,s_{0}\right)=O\left(n^{-\frac{1}{4}}\right) \end{array}$$ (117)

with probability $1-O(n^{\frac{1}{4}}\exp\left(-n^{\frac{1}{4}}\right))$, ∀*α* ∈ Γ^*ϵ*^. Note that we will write “with probability $1-O(n^{\frac{1}{4}}\exp\left(-n^{\frac{1}{4}}\right))$” as whp hereinafter.

In particular, we have that

$$\begin{array}{c} \frac{S_{k_{1}-1}^{\alpha }}{n}-s_{\frac{k_{1}-1}{n}}^{\alpha }\left(0,s_{0}\right)=O\left(n^{-\frac{1}{4}}\right)\;\;\text{whp.} \end{array}$$ (118)

Additionally by the Wormald’s theorem again we have that

$$\begin{array}{c} \underset{k_{1}\leq k\leq k_{2}-1}{\sup}\frac{S_{k}^{\alpha }}{n}-s_{\frac{k}{n}}^{\alpha }\left(\frac{k_{1}}{n},\frac{S_{k_{1}}}{n}\right)=O\left(n^{-\frac{1}{4}}\right)\;\;\text{whp.} \end{array}$$ (119)

Note that

$$\begin{array}{c} |\frac{S_{k_{1}}^{\alpha }}{n}-\frac{S_{k_{1}-1}^{\alpha }}{n}|\leq \frac{1}{n}\;\forall \alpha \in \Gamma^{\epsilon }, \end{array}$$ (120)

and by the Lipschitz continuity of $s_{\tau }^{\alpha }\left(0,s_{0}\right)$ on $\left(0,\tau_{1}^{-}\right)$,

$$\begin{array}{c} s_{\frac{k_{1}-1}{n}}^{\alpha }\left(0,s_{0}\right)-s_{\tau_{1}}^{\alpha }\left(0,s_{0}\right)=O\left(n^{-1}\right). \end{array}$$ (121)

So by Eqs (118), (120) and (121), we have

$$\begin{array}{cc} |\frac{S_{k_{1}}^{\alpha }}{n}-s_{\tau_{1}}^{\alpha }\left(0,s_{0}\right)|\leq & \left|\frac{S_{k_{1}}^{\alpha }}{n}-\frac{S_{k_{1}-1}^{\alpha }}{n}|+|\frac{S_{k_{1}-1}^{\alpha }}{n}-s_{\frac{k_{1}-1}{n}}^{\alpha }\left(0,s_{0}\right)\right|\\ & +|s_{\frac{k_{1}-1}{n}}^{\alpha }\left(0,s_{0}\right)-s_{\tau_{1}}^{\alpha }\left(0,s_{0}\right)|\\ = & n^{-1}+O\left(n^{-\frac{1}{4}}\right)+O\left(n^{-1}\right)\;\;\text{whp.} \end{array}$$ (122)

Thus we have that

$$\begin{array}{c} ∥\frac{S_{k_{1}}}{n}-s_{\tau_{1}}\left(0,s_{0}\right)∥=O\left(n^{-\frac{1}{4}}\right)+O\left(n^{-1}\right)\;\;\text{whp.} \end{array}$$ (123)

where ‖*η*‖ is the norm for $\eta \in R^{|\Gamma^{\in }|}$. We do not specify the norm because all norms in $R^{l}$ are equivalent, l∈N. From proposition 2 we see that the partial derivatives of $s_{\tau }^{\alpha }\left(\tau^{\prime },x\right)$ with respect to the initial time *τ*′ and every entry of *x* are continuous in *τ*′ and every entry of *x* respectively, and are bounded for any *τ* in a subinterval of $\left[0,\hat{\lambda }\right)$ on which $\tilde{u}$ is a constant vector function, i.e.

$$\begin{array}{c} ∥\frac{\partial s_{\tau }^{\alpha }\left(\tau^{\prime },x\right)}{\partial (\tau^{\prime },x)}∥\leq M_{1}<\infty \end{array}$$ (124)

where *M*_1_ is a constant. Recall that $|\frac{k_{1}}{n}-\tau_{1}|<n^{-1}$, so by Eqs (123) and (124), it follows from the fundamentals of calculus (e.g. theorem 9.19 and 9.21 in [31]) that

$$\begin{array}{cc} \; & s_{\tau }^{\alpha }\left(\frac{k_{1}}{n},\frac{S_{k_{1}}}{n}\right)-s_{\tau }^{\alpha }\left(\tau_{1},s_{\tau_{1}}\left(0,s_{0}\right)\right)\\ = & s_{\tau }^{\alpha }\left(\frac{k_{1}}{n},\frac{S_{k_{1}}}{n}\right)-s_{\tau }^{\alpha }\left(0,s_{0}\right)\\ = & O\left(n^{-\frac{1}{4}}\right)+O\left(n^{-1}\right)\;\;\text{whp}, \end{array}$$ (125)

for *τ* ∈ (*τ*_1_, *τ*_2_). So it follows from Eq (119) that ∀*α* ∈ Γ^*ϵ*^,

$$\begin{array}{c} \underset{k_{1}\leq k\leq k_{2}-1}{\sup}\frac{S_{k}^{\alpha }}{n}-s_{\frac{k}{n}}^{\alpha }\left(0,s_{0}\right)=O\left(n^{-\frac{1}{4}}\right)\;\;\text{whp.} \end{array}$$ (126)

Similarly for *R*_*k*_, define *r*_*τ*_(*τ*′, *x*, *y*) as the solution of

$$\begin{array}{c} \frac{d}{d\tau }r_{\tau }=h_{0}\left(\tau ,s_{\tau }\right), \end{array}$$ (127)

with the initial condition at *τ*′, (*s*_*τ*′_, *r*_*τ*′_) = (*x*, *y*). Applying the Wormald’s theorem for *R*_*k*_ and *r*_*τ*_ gives that,

$$\begin{array}{cc} \underset{0\leq k\leq k_{1}-1}{\sup}\frac{R_{k}}{n}-r_{\frac{k}{n}}\left(0,s_{0},0\right) & =O(n^{-\frac{1}{4}})\;\;\text{whp},\\ \underset{k_{1}\leq k\leq k_{2}-1}{\sup}\frac{R_{k}}{n}-r_{\frac{k}{n}}\left(\frac{k_{1}}{n},\frac{S_{k_{1}}}{n},\frac{R_{k_{1}}}{n}\right) & =O(n^{-\frac{1}{4}})\;\;\text{whp}. \end{array}$$ (128)

In particular,

$$\begin{array}{c} \frac{R_{k_{1}-1}}{n}-r_{\frac{k_{1}-1}{n}}\left(0,s_{0},0\right)=O\left(n^{-\frac{1}{4}}\right)\;\;\text{whp}, \end{array}$$ (129)

Further note that

$$\begin{array}{c} |\frac{R_{k_{1}}}{n}-\frac{R_{k_{1}-1}}{n}|\leq \frac{1}{n}\;\forall \alpha \in \Gamma^{\epsilon }, \end{array}$$ (130)

and by the Lipschitz continuity of *r*_*τ*_(0, *s*_0_, 0) on $\left(0,\tau_{1}^{-}\right)$,

$$\begin{array}{c} r_{\frac{k_{1}-1}{n}}\left(0,s_{0},0\right)-r_{\tau_{1}}\left(0,s_{0},0\right)=O\left(n^{-1}\right). \end{array}$$ (131)

So by Eqs (129), (130) and (131) we have

$$\begin{array}{cc} |\frac{R_{k_{1}}}{n}-r_{\tau_{1}}\left(0,s_{0},0\right)|\leq & \left|\frac{R_{k_{1}}}{n}-\frac{R_{k_{1}-1}}{n}|+|\frac{R_{k_{1}-1}}{n}-r_{\frac{k_{1}-1}{n}}\left(0,s_{0},0\right)\right|\\ & +|r_{\frac{k_{1}-1}{n}}\left(0,s_{0},0\right)-r_{\tau_{1}}\left(0,s_{0},0\right)|\\ = & n^{-1}+O\left(n^{-\frac{1}{4}}\right)+O\left(n^{-1}\right)\;\;\text{whp.} \end{array}$$ (132)

Here we apply the fact we shall prove later that the partial derivatives of *r*_*τ*_(*τ*′, *x*, *y*) with respect to the initial time *τ*′ and every entry of *x* and *y* are continuous in *τ*′, every entry of *x* and *y* respectively, and are bounded for any *τ* in a subinterval of $\left[0,\hat{\lambda }\right)$ on which $\tilde{u}$ is a constant vector function, i.e.

$$\begin{array}{c} ∥\frac{\partial r_{\tau }\left(\tau^{\prime },x,y\right)}{\partial (\tau^{\prime },x,y)}∥\leq M_{2}<\infty \end{array}$$ (133)

for some constant *M*_2_. Recall that $|\frac{k_{1}}{n}-\tau_{1}|<n^{-1}$, so by Eqs (123), (132) and (133), it follows from the fundamentals of calculus that

$$\begin{array}{cc} \; & r_{\tau }\left(\frac{k_{1}}{n},\frac{S_{k_{1}}}{n},\frac{R_{k_{1}}}{n}\right)-r_{\tau }\left(\tau_{1},s_{\tau_{1}}\left(0,s_{0}\right),r_{\tau_{1}}\left(0,s_{0},0\right)\right)\\ = & r_{\tau }\left(\frac{k_{1}}{n},\frac{S_{k_{1}}}{n},\frac{R_{k_{1}}}{n}\right)-r_{\tau }\left(0,s_{0},0\right)\\ = & O\left(n^{-\frac{1}{4}}\right)+O\left(n^{-1}\right)\;\;\text{whp}, \end{array}$$ (134)

for *τ* ∈ (*τ*_1_, *τ*_2_). So it follows from Eq (128) that

$$\begin{array}{c} \underset{k_{1}\leq k\leq k_{2}-1}{\sup}\frac{R_{k}}{n}-r_{\frac{k}{n}}\left(0,s_{0},0\right)=O\left(n^{-\frac{1}{4}}\right)\;\;\text{whp.} \end{array}$$ (135)

We can repeat the above procedure every time any $u_{\tau }^{\beta }$ has a step change, *β* ∈ Φ^*ϵ*^ and there are only a finite number of step changes in [0, λ). Because $s_{\tau }^{\alpha }\leq 1$ and *r*_*τ*_ ≤ λ, $d_{\infty }((s_{\tau },r_{\tau }),\partial U)\geq 0.1\geq Cn^{-\frac{1}{4}}$, for a sufficiently large constant *C*. Thus the supremum of *τ* that (*s*_*τ*_, *r*_*τ*_) can be extended to the boundary of *U* is $\hat{\lambda }$, i.e. in Eq (177) of the Wormald’s theorem in Appendix B: Wormald’s theorem,

$$\begin{array}{cc} \sigma & =\sup\{\tau \geq 0:\;d_{\infty }((s_{\tau },r_{\tau }),\partial U)\geq Cn^{-\frac{1}{4}}\}\\ & =\hat{\lambda }. \end{array}$$ (136)

So it follows that

$$\begin{array}{cc} \underset{0\leq k\leq n\hat{\lambda }}{\sup}\frac{S_{k}^{\alpha }}{n}-s_{\frac{k}{n}}^{\alpha }\left(0,s_{0}\right) & =O(n^{-\frac{1}{4}})\;\;\text{whp},\\ \underset{0\leq k\leq n\hat{\lambda }}{\sup}\frac{R_{k}}{n}-r_{\frac{k}{n}}\left(0,s_{0},0\right) & =O(n^{-\frac{1}{4}})\;\;\text{whp.} \end{array}$$ (137)

At last we prove the claim that the partial derivatives of *r*_*τ*_(*τ*′, *x*, *y*) with respect to the initial time *τ*′ and every entry of *x* and *y* are all continuous and bounded as in Eq (133) for any *τ* in a subinterval of $\left[0,\hat{\lambda }\right)$ on which $\tilde{u}$ is a constant vector function *b* = (*b*^*β*^)_*β*∈Φ^*ϵ*^_. Note first that *r*_*τ*_ with initial condition $\bar{s}=\left(s_{\tau^{\prime }},r_{\tau^{\prime }}\right)$ at *τ* = *τ*′ in a subinterval of $\left[0,\hat{\lambda }\right)$ on which $\tilde{u}=b$ satisfies that

$$\begin{array}{c} r_{\tau }=r_{\tau^{\prime }}+\int_{\tau^{\prime }}^{\tau }\sum_{\left(i,j,c,c-1\right)\in \Phi^{\epsilon }}\frac{\left(i-c+1\right)b^{i,j,c,c-1}}{\lambda -y}s_{y}^{i,j,c,c-1}\left(\tau^{\prime },s_{\tau^{\prime }}\right)dy. \end{array}$$ (138)

We shall prove the boundedness by showing the boundedness of $∥\frac{\partial r_{\tau }}{\partial \bar{s}}∥$ and $|\frac{\partial r_{\tau }}{\partial \tau^{\prime }}|$, seperately. First we take the derivatives of *r*_*τ*_ with respect to the initial condition $\bar{s}$ and obtain

$$\begin{array}{c} \frac{\partial r_{\tau }}{\partial \bar{s}}=e^{\text{last}}+\int_{\tau^{\prime }}^{\tau }\sum_{\left(i,j,c,c-1\right)\in \Phi^{\epsilon }}\frac{\left(i-c+1\right)b^{i,j,c,c-1}}{\lambda -y}\frac{\partial s_{y}^{i,j,c,c-1}\left(\tau^{\prime },s_{\tau^{\prime }}\right)}{\partial \bar{s}}dy, \end{array}$$ (139)

where *e*^last^ is a vector of zeros except an entry of one at the last. The continuity of every entry in $\frac{\partial r_{\tau }}{\partial \bar{s}}$ is obvious. For boundedness,

$$\begin{array}{c} ∥\frac{\partial r_{\tau }}{\partial \bar{s}}∥\leq 1+\int_{\tau^{\prime }}^{\tau }\sum_{\left(i,j,c,c-1\right)\in \Phi^{\epsilon }}\frac{\left(i-c+1\right)b^{i,j,c,c-1}}{\lambda -y}∥\frac{\partial s_{y}^{i,j,c,c-1}\left(\tau^{\prime },s_{\tau^{\prime }}\right)}{\partial \bar{s}}∥dy. \end{array}$$ (140)

By Eq (124), $∥\frac{\partial s_{y}^{i,j,c,c-1}}{\partial \bar{s}}∥<M_{1}$, so $∥\frac{\partial r_{\tau }}{\partial \bar{s}}∥$ is bounded. Next we take the derivative of *r*_*τ*_ with respect to the initial time *τ*′ by the Leibniz integral rule and obtain that

$$\begin{array}{cc} \frac{\partial r_{\tau }}{\partial \tau^{\prime }}= & -\sum_{\left(i,j,c,c-1\right)\in \Phi^{\epsilon }}\frac{\left(i-c+1\right)b^{i,j,c,c-1}}{\lambda -\tau^{\prime }}s_{\tau^{\prime }}^{i,j,c,c-1}\left(\tau^{\prime },s_{\tau^{\prime }}\right)\\ & +\int_{\tau^{\prime }}^{\tau }\sum_{\left(i,j,c,c-1\right)\in \Phi^{\epsilon }}\frac{\left(i-c+1\right)b^{i,j,c,c-1}}{\lambda -y}\frac{\partial s_{y}^{i,j,c,c-1}\left(\tau^{\prime },s_{\tau^{\prime }}\right)}{\partial \tau^{\prime }}dy, \end{array}$$ (141)

where $s_{\tau^{\prime }}^{i,j,c,c-1}\left(\tau^{\prime },s_{\tau^{\prime }}\right)=s_{\tau^{\prime }}^{i,j,c,c-1}$. The continuity of $\frac{\partial r_{\tau }}{\partial \tau^{\prime }}$ follows. By Eq (124), $|\frac{\partial s_{y}^{i,j,c,c-1}\left(\tau^{\prime },s_{\tau^{\prime }}\right)}{\partial \tau^{\prime }}|$ is bounded, so $|\frac{\partial r_{\tau }}{\partial \tau^{\prime }}|$ is bounded. We have proved Eq (133).

### Proof of proposition 4

*Proof*. For some [*τ*_1_, *τ*_2_) ⊆ [0, λ) on which *u*_*τ*_ is a constant vector function, by remark 5 we have for every fixed (*i*, *j*) pair and Γ_*i*,*j*_ = {(*c*, *l*):0 ≤ *l* < *c* ≤ *i* or *c* = *i* + 1, *l* = *i*} that

$$\begin{array}{c} \sum_{\left(c,l\right)\in \Gamma_{i,j}}s_{\tau }^{i,j,c,l}\leq \sum_{1\leq c\leq i}p\left(i,j,c\right), \end{array}$$ (142)

and thus it follows from Eq (23) that

$$\begin{array}{cc} 0\leq & \sum_{i\vee j\geq M^{\epsilon }}\sum_{0\leq c\leq i}jp\left(i,j,c\right)-\sum_{\left(i,j,c,l\right)\in \Gamma \backslash \Gamma^{\epsilon }}js_{\tau }^{i,j,c,l}\\ \leq & \sum_{i\vee j\geq M^{\epsilon }}\sum_{0\leq c\leq i}jp\left(i,j,c\right)<\epsilon . \end{array}$$ (143)

Similarly because by the definition of $S_{k}^{i,j,c,l}$ for 1 ≤ *k* ≤ *m*, for fixed (*i*, *j*) pair, (*i*, *j*, *c*, *l*) ∈ Γ,

$$\begin{array}{c} 0\leq \sum_{c,l}\frac{S_{k}^{i,j,c,l}}{n}\leq \sum_{1\leq c\leq i}P_{n}\left(i,j,c\right), \end{array}$$ (144)

thus it follows from Eq (25) that

$$\begin{array}{cc} 0\leq & \sum_{i\vee j\geq M^{\epsilon }}\sum_{0\leq c\leq i}jP_{n}\left(i,j,c\right)-\sum_{\left(i,j,c,l\right)\in \Gamma \backslash \Gamma^{\epsilon }}j\frac{S_{k}^{i,j,c,l}}{n}\\ \leq & \sum_{i\vee j\geq M^{\epsilon }}\sum_{0\leq c\leq i}jP_{n}\left(i,j,c\right)<\epsilon . \end{array}$$ (145)

For any *k* where $0\leq k\leq \hat{\lambda }$, by proposition 3, it follows that

$$\begin{array}{cc} |\frac{D_{k}^{-}}{n}-d_{\frac{k}{n}}^{-}|< & |\sum_{i\vee j<M^{\epsilon },0\leq c\leq i}jP_{n}\left(i,j,c\right)-\sum_{\left(i,j,c,l\right)\in \Gamma^{\epsilon }}j\frac{S_{k}^{i,j,c,l}}{n}\\ & -(\sum_{i\vee j<M^{\epsilon },0\leq c\leq i}jp\left(i,j,c\right)-\sum_{\left(i,j,c,l\right)\in \Gamma^{\epsilon }}js_{\frac{k}{n}}^{i,j,c,l})|+2\epsilon \\ = & |\sum_{i\vee j<M^{\epsilon },0\leq c\leq i}j(P_{n}\left(i,j,c\right)-p\left(i,j,c\right))\\ & -\sum_{\left(i,j,c,l\right)\in \Gamma^{\epsilon }}j(\frac{S_{k}^{i,j,c,l}}{n}-s_{\frac{k}{n}}^{i,j,c,l})|+2\epsilon \\ \leq & \sum_{i\vee j<M^{\epsilon },0\leq c\leq i}j|P_{n}\left(i,j,c\right)-p\left(i,j,c\right)|\\ & +\sum_{\left(i,j,c,l\right)\in \Gamma^{\epsilon }}j|\frac{S_{k}^{i,j,c,l}}{n}-s_{\frac{k}{n}}^{i,j,c,l}|+2\epsilon \\ \leq & M^{\epsilon }\left|\Gamma^{\epsilon }\right|(o\left(1\right)+o_{p}\left(1\right))+2\epsilon \\ = & o_{p}\left(1\right)+2\epsilon , \end{array}$$ (146)

and similarly,

$$\begin{array}{cc} |\frac{D_{k}}{n}-d_{\frac{k}{n}}|< & |\sum_{i\vee j<M^{\epsilon },0\leq c\leq i}P_{n}\left(i,j,c\right)-\sum_{\left(i,j,c,l\right)\in \Gamma^{\epsilon }}\frac{S_{k}^{i,j,c,l}}{n}\\ & -(\sum_{i\vee j<M^{\epsilon },0\leq c\leq i}p\left(i,j,c\right)-\sum_{\left(i,j,c,l\right)\in \Gamma^{\epsilon }}s_{\frac{k}{n}}^{i,j,c,l})|+2\epsilon \\ = & |\sum_{i\vee j<M^{\epsilon },0\leq c\leq i}(P_{n}\left(i,j,c\right)-p\left(i,j,c\right))\\ & -\sum_{\left(i,j,c,l\right)\in \Gamma^{\epsilon }}(\frac{S_{k}^{i,j,c,l}}{n}-s_{\frac{k}{n}}^{i,j,c,l})|+2\epsilon \\ \leq & \sum_{i\vee j<M^{\epsilon },0\leq c\leq i}|P_{n}\left(i,j,c\right)-p\left(i,j,c\right)|\\ & +\sum_{\left(i,j,c,l\right)\in \Gamma^{\epsilon }}|\frac{S_{k}^{i,j,c,l}}{n}-s_{\frac{k}{n}}^{i,j,c,l}|+2\epsilon \\ \leq & |\Gamma^{\epsilon }|(o\left(1\right)+o_{p}\left(1\right))+2\epsilon \\ = & o_{p}\left(1\right)+2\epsilon . \end{array}$$ (147)

### Proof of proposition 5

*Proof*. By Eq (25) for *n* large enough and 1 ≤ *k* ≤ *m*, we have

$$\begin{array}{cc} \; & \frac{1}{n}\sum_{\ell =1}^{k}\sum_{i\vee j\geq M^{\epsilon }}\sum_{1\leq c\leq i}1_{\left(W_{\ell }=\left(i,j,c,c-1\right)\right)}u_{\frac{\ell -1}{n}}^{i,j,c,c-1}\\ \leq & \frac{1}{n}\sum_{i\vee j\geq M^{\epsilon }}\sum_{1\leq c\leq i}i\;nP_{n}\left(i,j,c\right)\\ \leq & \sum_{i\vee j\geq M^{\epsilon },c}iP_{n}\left(i,j,c\right)<\epsilon . \end{array}$$ (148)

The first inequality holds because the number of times nodes with states in the range *i* ∨ *j* ≥ *M*^*ϵ*^, 1 ≤ *c* ≤ *i* are selected during the process is bounded above by their total in degree. Similarly by Eq (23), for $\tau \leq \hat{\lambda }$,

$$\begin{array}{cc} \; & \int_{0}^{\tau }\sum_{i\vee j\geq M^{\epsilon },1\leq c\leq i}\frac{\left(i-c+1\right)s_{t}^{i,j,c,c-1}}{\lambda -t}u_{t}^{i,j,c,c-1}dt\\ \leq & \int_{0}^{\tau }\sum_{i\vee j\geq M^{\epsilon },1\leq c\leq i}\frac{ip(i,j,c)}{\lambda -t}dt\\ \leq & \epsilon \int_{0}^{\tau }\frac{1}{\lambda -t}dt\\ = & \epsilon \ln\frac{\lambda }{\lambda -\tau }\\ \leq & \epsilon \ln\frac{\lambda }{\epsilon }=O\left(\epsilon \right). \end{array}$$ (149)

For any *k* where $0\leq k\leq n\hat{\lambda }$, by proposition 3 it follows that

$$\begin{array}{cc} |\frac{R_{k}}{n}-r_{\frac{k}{n}}|\leq & |\frac{{\tilde{R}}_{k}}{n}+\frac{1}{n}\sum_{\ell =1}^{k}\sum_{i\vee j\geq M^{\epsilon }}\sum_{1\leq c\leq i}1_{\left(W_{\ell }=\left(i,j,c,c-1\right)\right)}u_{\frac{\ell -1}{n}}^{i,j,c,c-1}\\ & -({\tilde{r}}_{\frac{k}{n}}+\int_{0}^{\frac{k}{n}}\sum_{i\vee j\geq M^{\epsilon },1\leq c\leq i}\frac{\left(i-c+1\right)s_{t}^{i,j,c,c-1}}{\lambda -t}u_{t}^{i,j,c,c-1}dt)|\\ \leq & |\frac{{\tilde{R}}_{k}}{n}-{\tilde{r}}_{\frac{k}{n}}|+O\left(\epsilon \right)\\ \leq & o_{p}\left(1\right)+O\left(\epsilon \right), \end{array}$$ (150)

thus we have that

$$\begin{array}{c} \underset{0\leq k\leq n\hat{\lambda }}{\sup}|\frac{R_{k}}{n}-r_{\frac{k}{n}}|=o_{p}\left(1\right)+O\left(\epsilon \right). \end{array}$$ (151)

If *τ*_*f*_ = λ, it implies that $d_{\tau }^{-}>0$ for $\tau \in (0,\hat{\lambda })$, then it follows from proposition 4 that $\frac{T_{n}}{n}=\hat{\lambda }+O\left(\epsilon \right)+o_{p}\left(1\right)$. Then because at each step there is at most one more node defaulting, $\frac{D_{T_{n}}}{n}=\frac{D_{\left\lfloor n\hat{\lambda }\right\rfloor }}{n}+O\left(\epsilon \right)+o_{p}\left(1\right)$ and from proposition 4 again, $\frac{D_{\left[n\hat{\lambda }\right]}}{n}=d_{\hat{\lambda }}+O\left(\epsilon \right)+o_{p}\left(1\right)$. ⌊⋅⌋ denotes the floor function. Further by the continuity of *d*_*τ*_ on [0, λ], $\frac{D_{T_{n}}}{n}=d_{\lambda }+O\left(\epsilon \right)+o_{p}\left(1\right)$. Similarly, by Eq (151) and the continuity of *r*_*τ*_ on [0, λ], we have that $\frac{R_{T_{n}}}{n}=r_{\lambda }+O\left(\epsilon \right)+o_{p}\left(1\right)$.

If *τ*_*f*_ < λ and $\frac{d}{d\tau }d_{\tau_{f}}^{-}<0$, by definition 4, *s*_*τ*_ is continuous and thus by Eq (33) $d_{\tau }^{-}$ is also continuous. So there exists some *τ*′ > 0 such that $d_{\tau }^{-}<0$ for *τ* ∈ (*τ*_*f*_, *τ*_*f*_ + *τ*′) by the continuity of $d_{\tau }^{-}$. Since *ϵ* is arbitrary, let *ϵ* be small enough such that $\inf_{\tau \in (\tau_{f},\tau_{f}+\tau^{\prime })}d_{\tau }^{-}<-2\epsilon$ and $\hat{\tau }$ be the first time $d_{\tau }^{-}$ reaches the minimum. Because $d_{\hat{\tau }}^{-}<-2\epsilon$, then by proposition 4 $\frac{D_{\left\lfloor n\hat{\tau }\right\rfloor }^{-}}{n}<0$ with high probability, so it holds that $\frac{T_{n}}{n}=\tau_{f}+O\left(\epsilon \right)+o_{p}\left(1\right)$. Again by the continuity of *d*_*τ*_ and *r*_*τ*_ on [0, λ], proposition 4 and Eq (151), $\frac{D_{T_{n}}}{n}=d_{\tau_{f}}+O\left(\epsilon \right)+o_{p}\left(1\right)$ and $\frac{R_{T_{n}}}{n}=r_{\tau_{f}}+O\left(\epsilon \right)+o_{p}\left(1\right)$.

In both cases we conclude that Eq (35) holds by tending *ϵ* → 0.

To prove Eq (37), since $R_{T_{n}}\leq m\leq \left(\lambda +0.1\right)n$ for large *n* and $D_{T_{n}}\leq n$, $\frac{R_{T_{n}}\left(G_{n},P_{n}\right)}{n}$ and $\frac{D_{T_{n}}\left(G_{n},P_{n}\right)}{n}$ are bounded and thus uniformly integrable. For a sequence of uniformly integrable random variables, convergence in probability implies convergence in expectation. Therefore Eq (37) holds.

### Proof of lemma 1

*Proof*. Solve (FOCP) for the optimal $\left(\tilde{u},{\tilde{\tau }}_{f}\right)$. Note that ${\tilde{u}}^{\beta }=0$ for *β* ∈ Φ∖Φ^*ϵ*^. If there exists some *p*(*i*, *j*, *c*)>0, *i* ∨ *j* ≥ *M*^*ϵ*^, 0 ≤ *c* ≤ *i*, then by remark 5, at ${\tilde{\tau }}_{f}$ by summing over (*i*, *j*) pairs satisfying *i* ∨ *j* ≥ *M*^*ϵ*^ we can show that

$$\begin{array}{c} \sum_{i\vee j\geq M^{\epsilon },0\leq c\leq i}p\left(i,j,c\right)-\sum_{\left(i,j,c,l\right)\in \Gamma \backslash \Gamma^{\epsilon }}s_{{\tilde{\tau }}_{f}}^{i,j,c,l}\geq 0, \end{array}$$ (152)

and by the definition of ${\tilde{\tau }}_{f}$ that

$$\begin{array}{cc} d_{{\tilde{\tau }}_{f}}^{-}= & \sum_{i\vee j\geq M^{\epsilon },0\leq c\leq i}jp\left(i,j,c\right)-\sum_{\left(i,j,c,l\right)\in \Gamma \backslash \Gamma^{\epsilon }}js_{{\tilde{\tau }}_{f}}^{i,j,c,l}\\ & +\sum_{i\vee j<M^{\epsilon },0\leq c\leq i}jp\left(i,j,c\right)-\sum_{\left(i,j,c,l\right)\in \Gamma^{\epsilon }}js_{{\tilde{\tau }}_{f}}^{i,j,c,l}-{\tilde{\tau }}_{f}\\ = & \sum_{i\vee j\geq M^{\epsilon },0\leq c\leq i}jp\left(i,j,c\right)-\sum_{\left(i,j,c,l\right)\in \Gamma \backslash \Gamma^{\epsilon }}js_{{\tilde{\tau }}_{f}}^{i,j,c,l}\geq 0. \end{array}$$ (153)

Now we construct a function *u* as $u_{\tau }={\tilde{u}}_{\tau }$, $\tau \leq {\tilde{\tau }}_{f}$ and $u_{\tau }^{\beta }=1$, ${\tilde{\tau }}_{f}<\tau$, *β* ∈ Φ. Note that under *u*, there are always interventions after ${\tilde{\tau }}_{f}$, thus by remark 5, for a fixed (*i*, *j*) pair with the set Γ_*i*,*j*_ = {(*c*, *l*):0 ≤ *l* < *c* ≤ *i* or *c* = *i* + 1, *l* = *i*}, $\sum_{\left(c,l\right)\in \Gamma_{i,j}}s_{\tau }^{i,j,c,l}$ will not change, i.e. $\sum_{\left(c,l\right)\in \Gamma_{i,j}}s_{\tau }^{i,j,c,l}=\sum_{\left(c,l\right)\in \Gamma_{i,j}}s_{{\tilde{\tau }}_{f}}^{i,j,c,l}$ for $\tau >{\tilde{\tau }}_{f}$. Let *τ*_*f*_ be the solution of $d_{\tau_{f}}^{-}=0$ under *u*, then it follows that

$$\begin{array}{cc} d_{\tau_{f}}-{\tilde{d}}_{{\tilde{\tau }}_{f}}= & \sum_{i,j,0\leq c\leq i}p\left(i,j,c\right)-\sum_{(i,j,c,l)\in \Gamma }s_{\tau_{f}}^{i,j,c,l}\\ & -(\sum_{i\vee j<M^{\epsilon },0\leq c\leq i}p\left(i,j,c\right)-\sum_{\left(i,j,c,l\right)\in \Gamma^{\epsilon }}s_{{\tilde{\tau }}_{f}}^{i,j,c,l})\\ = & \sum_{i\vee j\geq M^{\epsilon },0\leq c\leq i}p\left(i,j,c\right)-\left(\sum_{(i,j,c,l)\in \Gamma }s_{{\tilde{\tau }}_{f}}^{i,j,c,l}-\sum_{\left(i,j,c,l\right)\in \Gamma^{\epsilon }}s_{{\tilde{\tau }}_{f}}^{i,j,c,l}\right)\\ = & \sum_{i\vee j\geq M^{\epsilon },0\leq c\leq i}p\left(i,j,c\right)-\sum_{\left(i,j,c,l\right)\in \Gamma \backslash \Gamma^{\epsilon }}s_{{\tilde{\tau }}_{f}}^{i,j,c,l}\\ \leq & \sum_{i\vee j\geq M^{\epsilon },0\leq c\leq i}p\left(i,j,c\right)<\epsilon , \end{array}$$ (154)

and similarly

$$\begin{array}{cc} r_{\tau_{f}}-{\tilde{r}}_{{\tilde{\tau }}_{f}} & =\sum_{i\vee j\geq M^{\epsilon },0\leq c\leq i}jp\left(i,j,c\right)-\sum_{\left(i,j,c,l\right)\in \Gamma \backslash \Gamma^{\epsilon }}js_{{\tilde{\tau }}_{f}}^{i,j,c,l}\\ & \leq \sum_{i\vee j\geq M^{\epsilon },0\leq c\leq i}jp\left(i,j,c\right)<\epsilon . \end{array}$$ (155)

By the definition of *ζ* and $\tilde{\zeta }$,

$$\begin{array}{c} \zeta \left(u,\tau_{f},p\right)\leq \tilde{\zeta }\left(\tilde{u},{\tilde{\tau }}_{f},p\right)+\left(K+1\right)\epsilon . \end{array}$$ (156)

Recall $\left(u^{*},\tau_{f}^{*}\right)$ is the optimal solution for the infinite dimensional Eq (39). By remark 6, (FOCP) assumes that the high degree nodes are invulnerable and because the $\left(\tilde{u},{\tilde{\tau }}_{f}\right)$ is the optimal solution for (FOCP), it provides the lower bound for the optimal objective function of the infinite dimensional Eq (39), i.e.

$$\begin{array}{c} \tilde{\zeta }\left(\tilde{u},{\tilde{\tau }}_{f},p\right)\leq \zeta \left(u^{*},\tau_{f}^{*},p\right). \end{array}$$ (157)

Let the objective function be *ζ*(*u*, *τ*_*f*_, *p*) under *u*, then by the optimality of $\left(u^{*},\tau_{f}^{*}\right)$, we have that

$$\begin{array}{c} \zeta \left(u^{*},\tau_{f}^{*},p\right)\leq \zeta \left(u,\tau_{f},p\right). \end{array}$$ (158)

In sum, we have that

$$\begin{array}{c} \tilde{\zeta }\left(\tilde{u},{\tilde{\tau }}_{f},p\right)\leq \zeta \left(u^{*},\tau_{f}^{*},p\right)\leq \zeta \left(u,\tau_{f},p\right)\leq \tilde{\zeta }\left(\tilde{u},{\tilde{\tau }}_{f},p\right)+\left(K+1\right)\epsilon . \end{array}$$ (159)

Thus the conclusion follows.

### Proof of proposition 6

*Proof*. We apply the Extended Pontryagin Maximum Principle (EPMP) in Appendix C: Extended pontryagin maximum principle. First we present the correspondence of a notation A in EPMP and B in our application in the form *A* → *B*.

$$\begin{array}{cc} t\to & t,\\ t_{0}\to & t_{0},\\ t_{f}\to & t_{f},\\ {\left(x_{t}^{i}\right)}_{i\in \{1,\ldots ,n_{x}\}}\to & {\left(s_{t}^{\alpha }\right)}_{\alpha \in \Gamma^{\epsilon }},\\ {\left(u_{t}^{i}\right)}_{i\in \{1,\ldots ,n_{u}\}}\to & {\left(u_{t}^{\beta }\right)}_{\beta \in \Phi^{\epsilon }},\\ U\to & \{0,1\},\\ \overset{˚}{\lambda }\to & \overset{˚}{w},\\ {\left(\lambda_{t}^{i}\right)}_{i\in \{1,\ldots ,n_{x}\}}\to & {\left(w_{t}^{\alpha }\right)}_{\alpha \in \Gamma^{\epsilon }},\\ \ell (t,x_{t},u_{t})\to & K\sum_{i,j,1\leq c\leq i}\left(i-c+1\right)s_{t}^{i,j,c,c-1}u_{t}^{i,j,c,c-1},\\ \phi (t_{f},x_{t_{f}})\to & d_{t_{f}},\\ \psi_{k}\left(t_{f},x_{t_{f}}\right)=0,\;k=1,\ldots ,n_{\psi }\to & d_{t_{f}}^{-}=0. \end{array}$$ (160)

Let $\left(\overset{˚}{w},w_{t}\right)$ be the adjoint variables then $\overset{˚}{w}=1$, since otherwise the necessary conditions of optimality becomes independent of the cost functional in Eq (42). The Hamiltonian function Eq (48) is a direct result of Eq (183). Note that *n*_*ψ*_ = 1 and

$$\begin{array}{c} \psi \left(t,s\right)=\sum_{i,j,0\leq c\leq i}jp\left(i,j,c\right)-\sum_{\left(i,j,c,l\right)\in \Gamma^{\epsilon }}js^{i,j,c,l}-\lambda \left(1-e^{t_{0}-t}\right). \end{array}$$ (161)

Taking partial derivative yields $\frac{\partial }{\partial s}\psi \left(t_{f},s_{t_{f}}\right)=\left(j,j,\ldots ,j\right)$ which has rank 1.

Since the Hamiltonian function is affine in the control variable *u*_*t*_, by condition (1) of EPMP, we attain that, for 1 ≤ *c* ≤ *i*,

$$\begin{array}{c} u_{t}^{i,j,c,c-1}=\{\begin{array}{cc} 0 & \;\text{if}\;\left(K+w_{t}^{i,j,c+1,c}\right)s_{t}^{i,j,c,c-1}>0\\ 1 & \;\text{if}\;\left(K+w_{t}^{i,j,c+1,c}\right)s_{t}^{i,j,c,c-1}<0\\ 0\;\text{or}\;1 & \text{if}\;\left(K+w_{t}^{i,j,c+1,c}\right)s_{t}^{i,j,c,c-1}=0. \end{array} \end{array}$$ (162)

By distinguishing the two cases $s_{t}^{i,j,c,c-1}>0$ and $s_{t}^{i,j,c,c-1}=0$, we have the equivalent form in Eq (50).

Taking partial derivative of H with regard to *s* yields the differential equations of *w*_*t*_ in condition (2). Note that H is autonomous, then according to condition (3) of EPMP, $H(s_{t},u_{t},w_{t})$ is a constant for *t* ∈ [*t*_0_, *t*_*f*_], which is condition (3).

Then define

$$\begin{array}{cc} \Psi (t,s)≔ & \sum_{i,j,0\leq c\leq i}p\left(i,j,c\right)-\sum_{\left(i,j,c,l\right)\in \Gamma^{\epsilon }}s^{i,j,c,l}\\ & +v(\sum_{i,j,0\leq c\leq i}jp\left(i,j,c\right)-\sum_{\left(i,j,c,l\right)\in \Gamma^{\epsilon }}js_{t_{f}}^{i,j,c,l}-\lambda \left(1-e^{t_{0}-t_{f}}\right)) \end{array}$$ (163)

and taking partial derivatives with respect to *s* and *t* respectively by condition (4) of EPMP together with the terminal condition Eq (47) leads to condition (4).

### Proof of theorem 1

*Proof*. For the contagion process without intervention, we relate our model to the auxiliary model used in the proof of theorem 3.8 in [24].

Recall that in Dynamics we are given a set of nodes [*n*] and the degree sequence (*d*^−^(*v*), *d*^+^(*v*))_*v*∈[*n*]_ as well as the initial equity levels (*e*(*v*))_*v*∈[*n*]_ and the network is constructed sequentially by matching any out half-link from the default set to a uniformly chosen unconnected in half-link at every step. For each node *v* we assign each in half-link a number ranging in {1,…,*d*^−^(*v*)}. Let ∑^*v*^ be the set of all permutations of the in half-links of node *v* ∈ [*n*], then a permutation π ∈ ∑^*v*^ specifies the order in which the in half-links are connected.

Because every in half-link of *v* represents one unit of loan, *v* will default after *e*(*v*) of its in half-links have been connected (or *e*(*v*) of its in links have been revealed) for every permutation *π* ∈ *Σ*^*v*^. So the default threshold *θ*(*v*, *π*) for node *v* if the order in which the in half-links are connected is specified by *π* is *θ*(*v*, *π*) = *e*(*v*), ∀*π* ∈ *Σ*^*v*^. Further our assumption 1 is equivalent to the assumption 4.1 and 4.2 in [24]. Moreover, under no intervention, the random graph generated in Dynamics conforms to the model defined in definition 5.4 in [24] with in and out degree sequences (*d*^−^(*v*), *d*^+^(*v*))_*v*∈[*n*]_ and default thresholds (*e*(*v*))_*v*∈[*n*]_ So by theorem 3.8 in [24] we achieve the conclusions of theorem 1.

### Proof of theorem 2

*Proof*. To simplify the notations we suppress the apostrophe “*”. In lemma 2 we have presented the optimal control policy (*u*_*t*_)_*t*∈[*t*_0_,*t*_*f*_]_ in terms of *t*,*t*_0_,*t*_*f*_,*t*_*s*_,*t*^*i*,*j*,*c*^. Recall that in Eq (77) we have the following relations,

$$\begin{array}{cc} y= & 1-e^{t_{0}-t_{f}},\\ z= & 1-e^{t_{0}-t_{s}},\\ x^{i,j,c,c-1}= & 1-e^{t_{0}-t^{i,j,c}}\\ = & \left\{ \begin{array}{cc} y & \text{if}\;K+vj-1\geq 0\;\text{or}\;c=0\\ 1-\left(1-y\right)\frac{(i-c)K}{(i-c+1)K+vj-1} & \text{if}\;K+vj-1<0\\ & \;\text{and}\;1\leq c<i+\frac{K+vj-1}{Ky}\\ 0 & \text{otherwise}, \end{array}\right. \end{array}$$ (164)

as well as *t* = −ln(λ − *τ*), *t*_0_ = −ln λ, so we can change the variable from *t* to *τ*. Particularly we apply mapping *f*(*t*) = 1 − *e*^*t*_0_−*t*^ which is strictly increasing in *t*, then we have

$$\begin{array}{cc} f(t) & =\frac{\tau }{\lambda },\\ f(t_{0}) & =0,\\ f(t^{i,j,c}) & =x^{i,j,c,c-1},\\ f(t_{s}) & =z,\\ f(t_{f}) & =y. \end{array}$$ (165)

We replace each variable *t*, *t*_0_, *t*_*f*_, *t*_*s*_, *t*^*i*,*j*,*c*^ in lemma 2 with its corresponding variable in Eq (165) resulting in the expressions for $u_{\tau }^{i,j,c,c-1}$. At last by assumption 2 on the relations between the control policy $G_{n}=\left(g_{1}^{\left(n\right)},\ldots ,g_{m}^{\left(n\right)}\right)$ and the function *u*, we have the conclusion in theorem 2.

### Proof of theorem 3

*Proof*. In proposition 7 we have obtained the expressions for $d_{t_{f}}^{-}$ and $d_{t_{f}}$ with *i* ∨ *j* < *M*^*ϵ*^ in terms of (*v*, *t*_*f*_, *t*_*s*_), after change of variables to (*v*, *y*, *z*) with $y=\frac{\tau_{f}}{\lambda }$ we have the following expressions for $d_{\tau_{f}}^{-}$ and $d_{\tau_{f}}$ with their relations to $\tilde{I}\left(y;v,z\right)$ and $\tilde{J}\left(y;v,z\right)$ in definition 10.

$$\begin{array}{cc} d_{\tau_{f}}^{-}= & \sum_{i\vee j<M^{\epsilon }}j[\sum_{c=0}^{i}p\left(i,j,c\right)P\left(\text{Bin}\left(i,x^{i,j,c,c-1}\right)\geq c\right)\\ & -1_{\left(vj-1=-K\right)}p\left(i,j,i\right)({\left(\frac{\tau_{f}}{\lambda }\right)}^{i}-z^{i})]-\tau_{f}\\ = & \lambda (\tilde{I}(\frac{\tau_{f}}{\lambda };v,z)-\frac{\tau_{f}}{\lambda }),\\ d_{\tau_{f}}= & \sum_{i\vee j<M^{\epsilon }}[\sum_{c=0}^{i}p\left(i,j,c\right)P\left(\text{Bin}\left(i,x^{i,j,c,c-1}\right)\geq c\right)\\ & -1_{\left(vj-1=-K\right)}p\left(i,j,i)({\left(\frac{\tau_{f}}{\lambda }\right)}^{i}-z^{i})\right]\\ = & \tilde{J}(\frac{\tau_{f}}{\lambda };v,z). \end{array}$$ (166)

Suppose (y*, v*, z*) is an optimal solution for the optimization problem Eq (86) and note that y* is the smallest fixed point of $\tilde{I}\left(y;v^{*},z^{*}\right)$ and $y^{*}=\frac{\tau_{f}^{*}}{\lambda }$.

1. If y* = 1, then $\tau_{f}^{*}=\lambda$. By the definition of $d_{\tau_{f}}^{-}$, it can only occur when $\sum_{i\vee j<M^{\in }}j\sum_{c=0}^{i}p\left(i,j,c\right)=\lambda$ and $z^{*}=\frac{\tau_{f}^{*}}{\lambda }=1$, thus we have $d_{\tau_{f}^{*}}=d_{\lambda }=1$, then by proposition 5,
  $$\begin{array}{c} \frac{D_{T_{n}}}{n}\overset{p}{\to }1, \end{array}$$ (167)
  which proves (1) of theorem 3.
2. If y* < 1 and ${\tilde{I}}^{\prime }\left(y^{*};v^{*},z^{*}\right)<1$, then $\tau_{f}^{*}<\lambda$ and $\frac{d}{d\tau }d_{\tau_{f}^{*}}^{-}={\tilde{I}}^{\prime }\left(\frac{\tau_{f}^{*}}{\lambda };v^{*},z^{*}\right)-1<0$. Again it follows from proposition 5,
  $$\begin{array}{c} \frac{D_{T_{n}}}{n}\overset{p}{\to }d_{\tau_{f}^{*}}=\tilde{J}\left(y^{*};v^{*},z^{*}\right). \end{array}$$ (168)
  which proves (2) of theorem 3. This concludes the proof of theorem 3.

It is important to note that the two cases in theorem 3 corresponds to $\tau_{f}^{*}=\lambda$, and $\tau_{f}^{*}<\lambda$, $\frac{d}{d\tau }d_{\tau_{f}^{*}}^{-}<0$, respectively. By proposition 5 they guarantees that the limits of $E\frac{R_{T_{n}}\left(G_{n},P_{n}\right)}{n}$ and $E\frac{D_{T_{n}}\left(G_{n},P_{n}\right)}{n}$ in Eq 9 as *n* → ∞ are well defined, which are $r_{\tau_{f}}$ and $d_{\tau_{f}}$, respectively.

### Proof of lemma 4

*Proof*. In the following we suppress “*”. Note first that if *v* > 0, *x*^*i*,*j*,*c*,*c*−1^ is increasing in *j*. This implies that *x*^*i*,*j*_1_,*c*,*c*−1^ < *x*^*i*,*j*_2_,*c*,*c*−1^ for the two states in Φ^*ϵ*^, (*i*, *j*_1_, *c*, *c* − 1) and (*i*, *j*_2_, *c*, *c* − 1) where *j*_1_ < *j*_2_. By theorem 2, this further implies that at some step *k* such that *nλx*^*i*,*j*_1_,*c*,*c*−1^ ≤ *k* ≤ *nλx*^*i*,*j*_2_,*c*,*c*−1^, we should intervene on a node in state (*i*, *j*_1_, *c*, *c* − 1) when it is selected at *k* but not on a node in state (*i*, *j*_2_, *c*, *c* − 1). But this control policy is not optimal because both nodes are the same except the out degree and the node in state (*i*, *j*_2_, *c*, *c* − 1) is systematically more important.

## Appendix B: Wormald’s theorem

The following is from [30]. Let *a* ≥ 2 be a fixed integer and ${\left({\left(Y_{t}^{l}\right)}_{1\leq l\leq a}\right)}_{{}_{t\geq 0}}$ denote a sequence of real valued random variables indexed by *n* with its natural filtration ${\left(F_{t}\right)}_{t\geq 0}$. Assume that there is a constant *C*_0_ > 0 such that $|Y_{t}^{l}|\leq C_{0}n$ for ∀*n*, *t* ≥ 0 and 1 ≤ *l* ≤ *a*. Let $f_{l}:\;R^{a+1}\to R$ be functions and $U\subseteq R^{a+1}$ be some bounded connected open set containing the closure of

$$\begin{array}{c} \{\left(0,z^{1},\ldots ,z^{a}\right):\;P\left(Y_{0}^{l}=z^{l}n,\;1\leq l\leq a\right)\neq 0\;\text{for}\;\text{some}\;n\}. \end{array}$$ (169)

Define the stopping time $T_{U}=\inf\{t\geq 1:\;(\frac{t}{n},\frac{Y_{t}^{1}}{n},\ldots ,\frac{Y_{t}^{a}}{n})\notin U\}$. Assume the following three conditions are satisfied:

1. (Boundedness) For some function *ρ*_1_ = *ρ*_1_(*n*)≥1 and ∀*t* < *T*_*U*_ and 1 ≤ *l* ≤ *a*,
  $$\begin{array}{c} |Y_{t+1}^{l}-Y_{t}^{l}|\leq \rho_{1}. \end{array}$$ (170)
2. (Trend) For some function *ρ*_2_ = *ρ*_2_(*n*) = *o*(1) and ∀*t* < *T*_*U*_ and 1 ≤ *l* ≤ *a*,
  $$\begin{array}{c} |E(Y_{t+1}^{l}-Y_{t}^{l}|F_{t})-f_{l}\left(\frac{t}{n},\frac{Y_{t}^{1}}{n},\ldots ,\frac{Y_{t}^{a}}{n}\right)|\leq \rho_{2}. \end{array}$$ (171)
3. (Lipschitz continuity) The functions (*f*_*l*_)_1≤*l* ≤ *a*_ are continuous and satisfies a Lipschitz condition on
  $$\begin{array}{c} U\cap \{\left(t,z^{1},\ldots ,z^{a}\right):t\geq 0\} \end{array}$$ (172)
  with the same Lipschitz constant for each *l*.

Then the following holds:

1. For $(0,{\hat{z}}^{1},\ldots ,{\hat{z}}^{a})\in U$ the system of differential equations
  $$\begin{array}{c} \frac{dz^{l}}{ds}=f_{l}\left(s,z^{1},\ldots ,z^{a}\right),\;1\leq l\leq a \end{array}$$ (173)
  has a unique solution in *U* for $z^{l}:R\to R$ passing through
  $$\begin{array}{c} z_{0}^{l}={\hat{z}}^{l},\;1\leq l\leq a \end{array}$$ (174)
  and which extends to points arbitrarily close to the boundary of *U*.
2. Let *ρ* > *ρ*_2_ and *ρ* = *o*(1). For a sufficiently large constant C, with probability $1-O(\frac{\rho_{1}}{\rho }\exp(-\frac{n\rho^{3}}{\rho_{1}^{3}}))$, it holds that
  $$\begin{array}{c} \underset{0\leq t\leq n\sigma }{\sup}(\frac{Y_{t}^{l}}{n}-z_{\frac{t}{n}}^{l})=O\left(\rho \right) \end{array}$$ (175)
  where $z_{s}^{l}$ is the solution in (1) with
  $$\begin{array}{c} z_{0}^{l}=\frac{Y_{0}^{l}}{n} \end{array}$$ (176)
  and
  $$\begin{array}{c} \sigma =\sigma \left(n\right)=\sup\{s\geq 0:\;d_{\infty }(({\left(z_{s}^{l}\right)}_{1\leq l\leq a}),\partial U)\geq C\rho \}, \end{array}$$ (177)
  where *d*_∞_(*u*, *v*) = max_1≤*i* ≤ *j*_|*u*_*i*_−*v*_*i*_| for $u=\left(u_{1},\ldots ,u_{j}\right)\in R^{j}$ and $v=\left(v_{1},\ldots ,v_{j}\right)\in R^{j}$.

## Appendix C: Extended pontryagin maximum principle

The following is from [32]. Consider the optimal control problem to minimize the cost functional including a terminal term

$$\begin{array}{c} J(u,t_{f})≔\int_{t_{0}}^{t_{f}}\ell \left(t,x_{t},u_{t}\right)dt+\phi \left(t_{f},x_{t_{f}}\right), \end{array}$$ (178)

with fixed initial time *t*_0_ and free terminal time *t*_*f*_, subject to the dynamical system

$$\begin{array}{c} {\dot{x}}_{t}=f\left(t,x_{t},u_{t}\right);\;x_{t_{0}}=x_{0}, \end{array}$$ (179)

where the vector function $x\in \hat{C^{1}}{\left[t_{0},T\right]}^{n_{x}}$ represents the state variables characterizing the behavior of the system at any time instant *t*, and some general terminal constraints

$$\begin{array}{c} \psi_{k}\left(t_{f},x_{t_{f}}\right)=0,\;k=1,\ldots ,n_{\psi }. \end{array}$$ (180)

The admissible controls shall be taken in the class of piecewise continuous functions

$$\begin{array}{c} u\in U[t_{0},T]≔\left\{u\in \hat{C}{\left[t_{0},T\right]}^{n_{u}}:u_{t}\in U\;\text{for}\;t_{0}\leq t\leq t_{f}\right\}, \end{array}$$ (181)

with *t*_*f*_ ∈ [*t*_0_, *T*], where *T* > *t*_0_ and the nonempty, possibly closed and nonconvex set *U* denotes the control region.

Suppose ℓ and *f* are continuous and have continuous first partial derivatives with respect to (*t*, *x*, *u*) on $\left[t_{0},T\right]\times R^{n_{x}}\times R^{n_{u}}$, and also *ϕ* and $\psi ≔{\left(\psi_{k}\right)}_{k=1,\ldots ,n_{\psi }}$ are continuous and have continuous first partial derivatives with respect to (*t*, *x*) on $\left[t_{0},T\right]\times R^{n_{x}}$. Suppose that the terminal constraints Eq (180) satisfy the constraint qualification

$$\begin{array}{c} \text{rank}(\frac{\partial \psi }{\partial x}\left(t_{f}^{*},x_{t_{f}^{*}}^{*}\right))=n_{\psi } \end{array}$$ (182)

where $\frac{\partial \psi }{\partial x}\left(t_{f}^{*},x_{t_{f}^{*}}^{*}\right)$ denotes the Jacobian matrix of the partial derivatives of components of *ψ* with respect to *x* evaluated at $\left(t_{f}^{*},x_{t_{f}^{*}}^{*}\right)$. Define the Hamiltonian function

$$\begin{array}{c} H\left(t,x,u,\overset{˚}{\lambda },\lambda \right)=\overset{˚}{\lambda }\ell \left(t,x,u\right)+\lambda^{T}f\left(t,x,u\right). \end{array}$$ (183)

Let $\left(u^{*},t_{f}^{*}\right)\in \hat{C}{\left[t_{0},T\right]}^{n_{u}}\times \left[t_{0},T\right)$ denote a minimizer for the problem, and $x^{*}\in \hat{C^{1}}\left[t_{0},T\right]$ the optimal state, then there exists a *n*_*x*_ dimensional piecewise continuously differentiable vector function $\lambda_{t}^{*}$ and ${\overset{˚}{\lambda }}^{*}\in \left\{0,1\right\}$ ($\left({\overset{˚}{\lambda }}^{*},\lambda_{t}^{*}\right)$ are called adjoint variables) and a Lagrange multiplier vector $v^{*}\in R^{n_{\psi }}$ such that $({\overset{˚}{\lambda }}^{*},\lambda_{t}^{*})\neq 0$ for every $t\in [t_{0},t_{f}^{*}]$ and the following conditions hold:

1. The function $H(t,x_{t}^{*},w,{\overset{˚}{\lambda }}^{*},\lambda_{t}^{*})$ attains its minimum on *U* at $w=u_{t}^{*}$ for every $t\in [t_{0},t_{f}^{*}]$, i.e.
  $$\begin{array}{c} H\left(t,x_{t}^{*},w,{\overset{˚}{\lambda }}^{*},\lambda_{t}^{*}\right)\geq H\left(t,x_{t}^{*},u_{t}^{*},{\overset{˚}{\lambda }}^{*},\lambda_{t}^{*}\right),\;\forall w\in U. \end{array}$$ (184)
2. $\left(x_{t}^{*},u_{t}^{*},{\overset{˚}{\lambda }}^{*},\lambda_{t}^{*}\right)$ verifies the equations
  $$\begin{array}{cc} \frac{d}{dt}x_{t}^{*} & =f(t,x_{t}^{*},u_{t}^{*}),\\ \frac{d}{dt}\lambda_{t}^{*} & =-\frac{\partial }{\partial x}H\left(t,x_{t}^{*},u_{t}^{*},{\overset{˚}{\lambda }}^{*},\lambda_{t}^{*}\right) \end{array}$$ (185)
  at each instant *t* of continuity of u* and ${\overset{˚}{\lambda }}^{*}\in \left\{0,1\right\}$.
3. $H\left(t,x_{t}^{*},u_{t}^{*},{\overset{˚}{\lambda }}^{*},\lambda_{t}^{*}\right)=H\left(t_{f}^{*},x_{t_{f}^{*}}^{*},u_{t_{f}^{*}}^{*},{\overset{˚}{\lambda }}^{*},\lambda_{t_{f}^{*}}^{*}\right)-\int_{t}^{t_{f}^{*}}\frac{\partial }{\partial t}H\left(\tau ,x_{\tau }^{*},u_{\tau }^{*},\overset{˚}{\lambda },\lambda_{\tau }^{*}\right)d\tau$. Therefore, if $\frac{\partial }{\partial t}H=0$, i.e. H is autonomous, then H is a constant over time.
4. (Transversal condition) Define $\Psi \left(t,x\right)≔{\overset{˚}{\lambda }}^{*}\phi \left(t,x\right)+v^{*T}\psi \left(t,x\right)$, then
  $$\begin{array}{cc} \lambda_{t_{f}^{*}}^{*}= & \frac{\partial }{\partial x}\Psi \left(t_{f}^{*},x_{t_{f}^{*}}^{*}\right),\\ H(t_{f}^{*},x_{t_{f}^{*}}^{*},u_{t_{f}^{*}}^{*},{\overset{˚}{\lambda }}_{t_{f}^{*}}^{*},\lambda_{t_{f}^{*}}^{*})= & -\frac{\partial }{\partial t}\Psi \left(t_{f}^{*},x_{t_{f}^{*}}^{*}\right) \end{array}$$ (186)
  together with the terminal condition Eq (180) at $t=t_{f}^{*}$, i.e. $\psi_{k}\left(t_{f}^{*},x_{t_{f}^{*}}^{*}\right)=0$ for *k* = 1, …, *n*_*ψ*_.
5. The optimal control u* may or may not be continuous; in the latter case we have a corner point. In particular, the conditions that must hold at any corner point $\theta \in [t_{0},t_{f}^{*}]$ are
  $$\begin{array}{cc} x_{\theta^{-}}^{*}= & x_{\theta^{+}}^{*},\\ \lambda_{\theta^{-}}^{*}= & \lambda_{\theta^{+}}^{*},\\ H(\theta^{-},x_{\theta }^{*},u_{\theta^{-}}^{*},{\overset{˚}{\lambda }}^{*},\lambda_{\theta }^{*})= & H(\theta^{+},x_{\theta }^{*},u_{\theta^{+}}^{*},{\overset{˚}{\lambda }}^{*},\lambda_{\theta }^{*}). \end{array}$$ (187)

*Proof*. See theorem 3.33 and theorem 3.34 in [32].

## Appendix D: Preliminary list of notations

$D_{k}$: the default set at step *k*;

$D_{k}=|D_{k}|$;

$D_{T_{n}}$: the number of defaulted nodes by the end of the process *T*_*n*_;

$D_{k}^{-}$: the number of unrevealed out links from the default set at step *k*;

$E_{n}$: the set of links in a random network;

*Gr*_*n*_: a graph on *n* nodes;

*G*_*n*,*m*_: the set of networks on *n* nodes with at most *m* directed links;

${\left(G_{k}\right)}_{0\leq k\leq m}$: the filtration on the default contagion process;

$G_{n}=\left(g_{1}^{\left(n\right)},\ldots ,g_{m}^{\left(n\right)}\right)$: a control policy for a network of size *n*;

*I*(*y*),*J*(*y*), $\tilde{I}\left(y,v,z\right)$, $\tilde{J}\left(y,v,z\right)$: special functions defined for theorem 1, theorem 2 and theorem 3;

*K*: the “cost” of an intervention relative to a defaulted node;

*M*^*ϵ*^: an integer defined based on *ϵ*;

$N_{0}≔\{0,1,2,\ldots \}$;

N≔{1,2,…}, the set of positive integers;

P: probability measure on the set *G*_*n*,*m*_;

*P*_*n*_(*i*, *j*, *c*): the empirical probability of the degrees and initial equity levels and *P*_*n*_ = (*P*_*n*_(*i*, *j*, *c*))_*i*,*j*,0≤*c*≤*i*_;

*Q*_*k*_: the set of hidden out links from the default set at step *k*;

*R*_*k*_: the accumulative number of interventions by step *k*;

$R_{T_{n}}$: the accumulative number of interventions by the end of the process *T*_*n*_;

$S_{k}^{i,j,c,l}$: the number of nodes that are vulnerable initially and in state (*i*, *j*, *c*, *l*) at step *k* and $S_{k}≔{\left(S_{k}^{\alpha }\right)}_{\alpha \in \Gamma }$;

*T*_*n*_: the contagion process end time;

U: the set of all ${\left(G_{k}\right)}_{0\leq k\leq m}$ adapted process *μ*;

(*V*_*k*_, *W*_*k*_): a pair of random variables denoting the link from node *V*_*k*_ to node *W*_*k*_ revealed at step *k*; By abuse of notation, *W*_*k*_ denotes the state of the node selected at *k* when there is no confusion;

*b* ≔ (*b*^*β*^)_*β*∈Φ_: a vector of {0, 1} constants;

$c_{k}^{v}$: the sum of initial equity and accumulative interventions of node *v* at step *k*;

*d*^−^(*v*),*d*^+^(*v*): the in and out degree of a node *v*;

$d_{\tau }^{-}$: the asymptotic number of unrevealed out links of the default set at time *τ*;

*d*_*τ*_: the asymptotic fraction of defaults at time *τ*;

*e*(*v*): the initial equity of a node;

$g_{k}^{\left(n\right)}$: the control function at step *k* for a network of size *n*;

*h*: the set of ODEs of *s*_*τ*_ and *s*_*t*_;

*h*′: the set of ODEs of *w*_*t*_;

$l_{k}^{v}$: the number of revealed in links of node *v* at step *k*;

*m* = *m*(*n*): the total in or out degree of a network of size *n*, maybe index variable as well;

*n*: the number of nodes, may be index variable as well;

[*n*] = {1, …, *n*};

$u_{\tau }^{i,j,c,c-1}$: a piecewise constant function; $u_{\tau }={\left(u_{\tau }^{\beta }\right)}_{\beta \in \Phi }$;

*r*_*τ*_: the asymptotic scaled number of interventions by time *τ*;

*v*: the Lagrange multiplier;

(*v*_*k*_, *w*_*k*_): the link from node *v*_*k*_ to node *w*_*k*_ revealed at step *k*;

$w_{t}^{i,j,c,l}$: the adjoint variable;

*ϵ*: a positive real;

λ: the asymptotic mean of in (out) degree;

$\hat{\lambda }≔\lambda -\in$;

*μ*_*k*_: intervention at step *k* and *μ* = (*μ*_*k*_)_1≤*k*≤*m*_;

*μ*_*n*_: in Introduction denotes an intervention sequence for a network of *n* nodes;

*τ*_*f*_: the end time in of the asymptotic process;

‖‖: any norm of $R^{l}$ for some l∈N;

⌈⋅⌉: the ceiling function;

[⋅]: the round function;

$\tilde{}$: the corresponding variable resulted by the constraint *i* ∨ *j* < *M*^*ϵ*^, e.g. ${\tilde{R}}_{k}$, $\tilde{u}$, ${\tilde{\tau }}_{f}$, ${\tilde{r}}_{\tau }$, ${\tilde{d}}_{\tau }$;

Bin(*i*, *y*): a binomial random variable in *i* trials with the probability of occurrence *y*;

Multin(*i*, *x*, *y*, 1 − *x* − *y*) = (*a*, *b*, *i* − *a* − *b*): a multinomial distribution in *i* trials with the probabilities of occurrence of each of three types being *x*, *y* and 1 − *x* − *y*, and turns out to have *a*, *b* and *i* − *a* − *b* occurrences of each type.

Important sets:

$$\begin{array}{cc} \Phi ≔ & \{(i,j,c,c-1):\;0\leq i,0\leq j,1\leq c\leq i\},\\ \Phi^{\epsilon }≔ & \{\left(i,j,c,c-1\right):i\vee j<M^{\epsilon },1\leq c\leq i\}.\\ \Gamma^{+}≔ & \{(i,j,c,l):0\leq i,0\leq j,0\leq c,0\leq l\leq i\},\\ \Gamma ≔ & \{(i,j,c,l):0\leq i,0\leq j,0\leq l<c\leq i\;\text{or}\;c=i+1,l=i\},\\ \Gamma^{\epsilon }≔ & \{\left(i,j,c,l\right):i\vee j<M^{\epsilon },0\leq l<c\leq i\;\text{or}\;c=i+1,l=i\},\\ \Gamma_{i,j}≔ & \{(c,l):0\leq l<c\leq i\;\text{or}\;c=i+1,l=i\}. \end{array}$$ (188)

## Supporting information

S1 DatasetDataset generated from the numerical experiments and based on which Figs 6–11 are presented.The spreadsheet contains two tables which includes respectively, under the optimal and alternative intervention policies the scaled number of interventions $\frac{R_{T_{n}}}{n}$, the scaled number of defaults $\frac{D_{T_{n}}}{n}$, the scaled process end time $\frac{T_{n}}{m}$ and the objective function value for the number of nodes *n* = 5^4^, 6^4^, …, 10^4^ and there are 100 runs for each *n*. The dataset is publicly available via https://figshare.com/articles/Simulation_result/7477562 with DOI 10.6084/m9.figshare.7477562.(XLSX)
